# Correction: Mediation role of low birth weight on the factors associated with newborn mortality and the moderation role of institutional delivery in the association of low birth weight with newborn mortality in a resource-poor setting

**DOI:** 10.1136/bmjopen-2020-046322corr1

**Published:** 2022-06-23

Kananura RM. Mediation role of low birth weight on the factors associated with newborn mortality and the moderation role of institutional delivery in the association of low birth weight with newborn mortality in a resource-poor setting. *BMJ Open* 2021;11:e046322. doi: 10.1136/bmjopen-2020-046322

This article was previously published with an error.

There was a mistake in calculating the indirect effect of the Perinatal Mortality (PM) factors that are mediated through Low Birth Weight (LBW).

Based on tables 1 and 2 results, the indirect effects are calculated as:

(1) $$indirect\;effect_{i}=\beta_{lbw\;i}*\beta_{lbw}x_{lbw}$$

Total effects are calculated as:

(2) $$total\;effect=\frac{\beta_{lbw\;i}*\beta_{lbw}x_{lbw}}{\beta_{lbwi}*\beta_{lbw}x_{lbw}+\beta_{pmi}}$$

where, $\beta_{lbw}x_{lbw}$ represents LBW coefficient in PM model, $\beta_{lbw\;i}$ represents variable coefficient in the LBW model and $\beta_{pm\;i}$ represents variable coefficient in the PM model that matches $\beta_{lbwi}$ . Table 1 presents results on direct factors of PM and table 2 presents results on indirect factors of PM that are mediated through LBW.

Based on the two equations, LBW mediated +47% of rural resident mothers, +54% of adolescent mothers, +15% of mothers with previous experience of new-born or pregnancy loss, +100% of multiple births and −45% of mothers with partners.

