# Supplementary material for: Mediation role of low birth weight on the factors associated with newborn mortality and the moderation role of institutional delivery in the association of low birth weight with newborn mortality in a resource-poor setting
Source: BMJ Open. 2021 May 22;11(5):e046322. doi: 10.1136/bmjopen-2020-046322 (PMC8149436; doi:10.1136/bmjopen-2020-046322)
Supplement: Supplementary data [file bmjopen-2020-046322supp001.pdf]

Supplement 1

Supplement 1 Table 1: Multicollinearity assessment for perinatal and neonatal mortality variables in the event history dataset

Variable	VIF	SQRT VIF	Tolerance	R-Squared
Birth season in quarters				
2	1.53	1.23	0.6557	0.3443
3	1.54	1.24	0.6481	0.3519
4	1.56	1.25	0.6404	0.3596
Child as Low birth weight	1.43	1.19	0.7006	0.2994
Child low birth weight and delivered in private facility	1.38	1.18	0.7238	0.2762
Child low birth weight and delivered in community	1.4	1.19	0.7118	0.2882
Primary Education	5.04	2.25	0.1982	0.8018
Post Primary Education	5.41	2.33	0.1848	0.8152
Delivered in private health facilities	1.3	1.14	0.7712	0.2288
Delivered in community	1.25	1.12	0.7991	0.2009
Maternal age				
<20 years	1.3	1.14	0.7707	0.2293
30+ years	1.19	1.09	0.8435	0.1565
Birth order				
1	1.39	1.18	0.7208	0.2792
5+	1.06	1.03	0.9397	0.0603
Rural residence	2.23	1.49	0.4477	0.5523
Has a partner	1.19	1.09	0.8412	0.1588
Experienced pregnancy or child loss previously	1.04	1.02	0.9631	0.0369
Multiple birth	1.02	1.01	0.9833	0.0167
Child sex as female	1	1	0.997	0.003
Wealth index				
Index 3	1.26	1.12	0.794	0.206
Index 4 and 5	2.81	1.68	0.3557	0.6443
Mean VIF	1.78			

Kananura RM. *BMJ Open* 2022; 11:e046322. doi: 10.1136/bmjopen-2020-046322

Supplement 1 Table 2: Assessment of missing variable factors for birth weight and wealth index in the event history dataset

	(1) Birth weight missingness	std.Err	(2) Wealth index missingness	std.Err
**Time of death**				
Day 1 and above	1	-	1	-
Still birth and day 0	9.137***	(12.47)	-	-
**Place of birth**			-	-
Hospital	1	-	-	-
Community	3.343***	(20.14)	-	-
Private	2.680***	(16.17)	-	-
**Place of residence**			-	-
Urban	1	-	-	-
Rural	1.779***	(10.28)	0.459***	(-13.56)
**Age group**				
<20 years	1.145	(1.94)	1.226*	(2.44)
20-29	1	-	1	-
30+	0.947	(-1.05)	0.619***	(-7.41)
**Education level**				
None	1	-	1	-
Primary	0.759***	(-4.21)	0.985	(-0.11)
Post primary	0.568***	(-7.60)	1.250	(1.68)
**Marital status**				
Has no a partner	-	-	1	-
Has a partner	-	-	2.010***	(7.38)

Exponentiated coefficients; t statistics in parentheses * p<0.05, ** p<0.01, *** p<0.001

Kananura RM. *BMJ Open* 2022; 11:e046322. doi: 10.1136/bmjopen-2020-046322

Supplement 1 Figure 1: Relationship between age and birth outcomes using for age categorization using event histories data

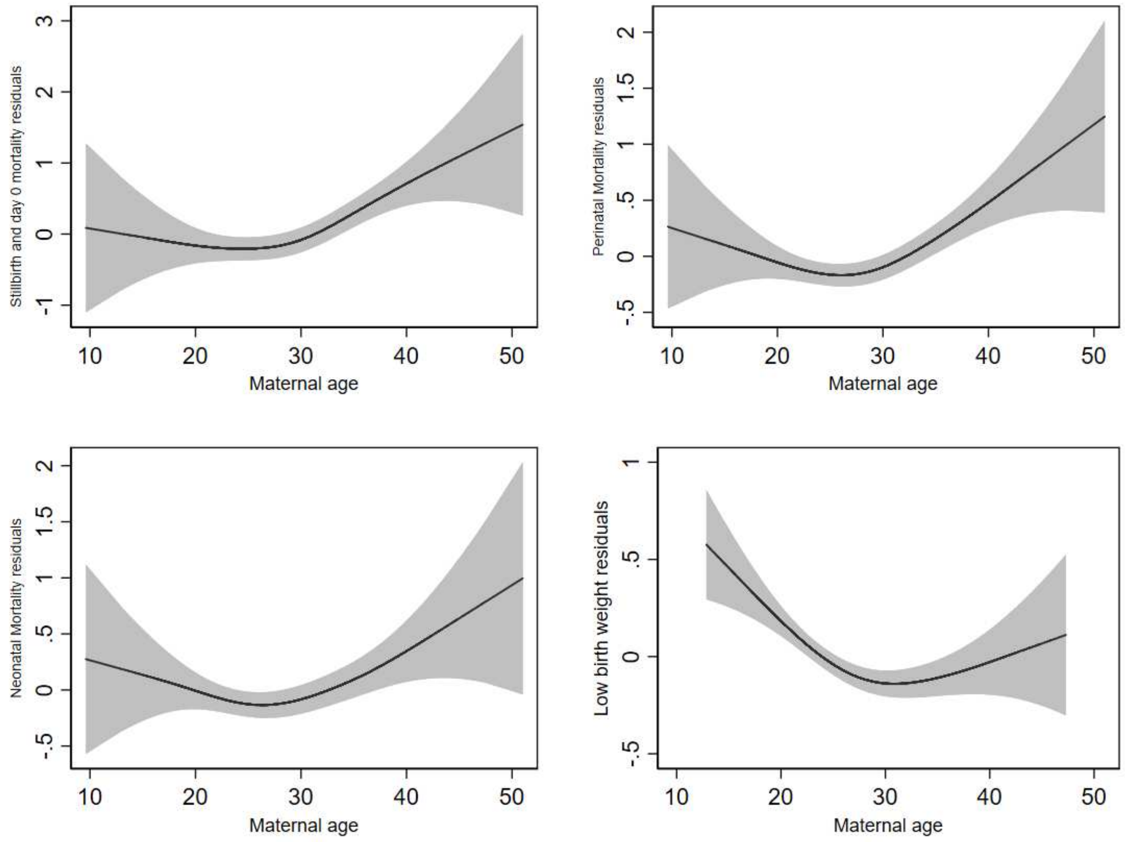

Kananura RM. *BMJ Open* 2022; 11:e046322. doi: 10.1136/bmjopen-2020-046322

Supplement 1 Figure 2: Comparing the LBW and household poor status for imputed and completed cases

I. **LBW estimates for imputed and completed cases analysis**

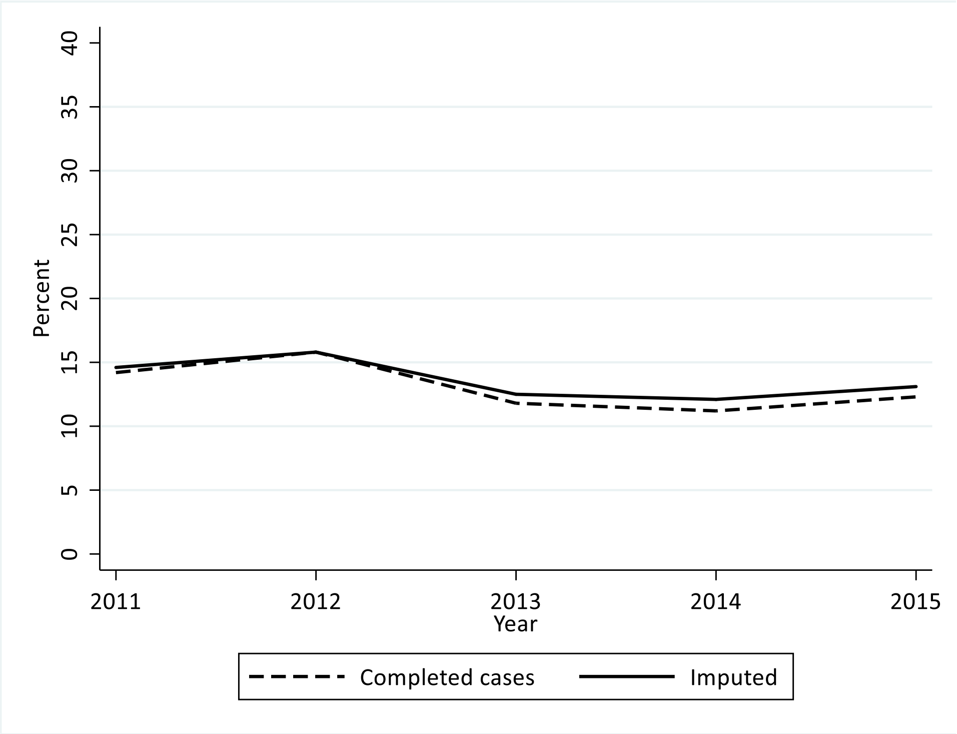

II. **Household poor status (wealth index 1 and 2) estimates for imputed and completed cases analysis**

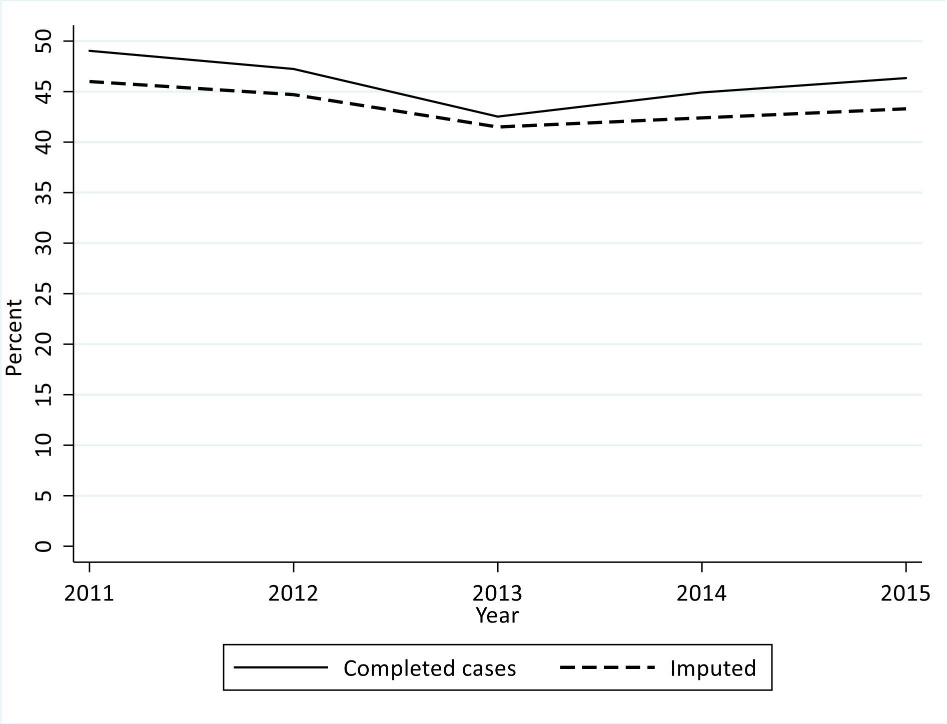

Supplement 1 Table 3: Bivariate analysis comparing logistic and firth logit model using 2011-2015 Iganga-Mayuge event history data

	Perinatal mortality				Late neonatal mortality			
	Logit		Firth logit		Logit		Firth logit	
	**OR**	**P-value**	**OR**	**P-value**	**OR**	**P-value**	**OR**	**P-value**
**Annual birth quarters**								
1	1.00	-	1.00	-	1.00	-	1.00	-
2	1.08	0.63	1.08	0.63	1.55	0.41	1.51	0.42
3	1.26	0.14	1.26	0.14	1.63	0.35	1.58	0.36
4	1.20	0.27	1.20	0.27	1.43	0.51	1.40	0.52
**Childbirth weight**								
<2.5 Kgs	2.52	<0.001	2.53	<0.001	2.37	0.05	2.45	0.04
25 Kgs+								
**Maternal education level**								
None	1.69	<0.001	1.70	<0.001	1.37	0.49	1.46	0.39
Primary+	1.00	-	1.00	-	1.00	-	1.00	-
**Place of delivery**								
Health facility	1.01	0.91	1.01	0.93	1.22	0.61	1.18	0.67
Community	1.00	-	1.00	-	1.00	-	1.00	-
**Maternal age (years)**								
<20	1.12	0.46	1.13	0.42	0.88	0.80	0.97	0.95
20—29	1.00	-	1.00	-	1.00	-	1.00	-
30+	1.48	<0.001	1.48	<0.001	1.73	0.12	1.74	0.11
**Childbirth order**								
1	0.80	0.07	0.80	0.07	0.70	0.32	0.71	0.33
2—4	1.00	-	1.00	-	1.00	-	1.00	-
5th+	0.46	0.06	0.49	0.07	2.20	0.20	2.52	0.10
**Place of residence**								
Urban	1.00	-	1.00	-	1.00	-	1.00	-
Rural	1.40	0.01	1.39	0.01	0.77	0.45	0.76	0.43
**Marital status**								
No partner	1.00	-	1.00	-	1.00	-	1.00	-
Has a partner	0.74	0.07	0.74	0.07	0.63	0.31	0.60	0.24
**Experienced neonatal or pregnancy loss previously**								
No								
Yes	4.18	0.00	4.20	<0.001	3.60	0.01	3.89	0.00
**Birth category**								
Singleton	1.00	-	1.00	-	1.00	-	1.00	-
Multiple	1.02	0.96	1.08	0.83	7.70	<0.001	8.31	0.00
**Child sex**								
Male	1.00	-	1.00	-	1.00	-	1.00	-
Female	1.06	0.68	1.06	0.68	0.57	0.12	0.58	0.12
**Household Wealth**								

	Perinatal mortality				Late neonatal mortality			
	Logit		Firth logit		Logit		Firth logit	
	OR	P-value	OR	P-value	OR	P-value	OR	P-value
Poor(er) (1-2)	1.10	0.43	1.10	0.43	0.73	0.39	0.75	0.41
Less poor (3-5)	1.00	-	1.00	-	1.00	-	1.00	-

Supplement 1 Table 4: Indirect pathways of the association between LBW and perinatal mortality.

Variables	Indirect effect	Se	P-value
Adolescence (<20 years)	0.03	0.12	0.84
Rural residence	0.11	0.06	0.05
Having a partner	0.08	0.06	0.14
Experienced neonatal or pregnancy loss previously	0.40	0.20	0.04
Multiple birth	1.14	0.15	<0.001

Kananura RM. *BMJ Open* 2022; 11:e046322. doi: 10.1136/bmjopen-2020-046322
