# Supplementary material for: Mediation role of low birth weight on the factors associated with newborn mortality and the moderation role of institutional delivery in the association of low birth weight with newborn mortality in a resource-poor setting
Source: BMJ Open. 2021 May 22;11(5):e046322. doi: 10.1136/bmjopen-2020-046322 (PMC8149436; doi:10.1136/bmjopen-2020-046322)
Supplement: Supplementary data [file bmjopen-2020-046322supp003.pdf]

Supplement 3 Table 1: 2011-2015 Iganga-Mayuge HDSS event histories participants' descriptive statistics

	Complete case*		Imputed
	n=10,758		
	%ge	*missing*	*%ge*
**Institutional delivery**		**1.0**	
Public Health facility	68.7		68.7
Community	16.0		16.0
Private health facility	15.4		15.4
**Maternal education level**		**0.5**	
None	14.3		14.3
Primary	52.3		52.3
Post primary	33.4		33.4
**Maternal age group (years)**		**0.0**	
<20	13.7		-
20-29	53.6		-
30+	32.8		-
**Place of residence**		**0.0**	
Rural	64.0		-
Urban	36.0		-
**Marital status**		**9.1**	
Has a partner or married	86.2		86.2
Has no a partner	13.8		13.8
**Household wealth index**		**18.8**	
Index 1 and 2	46.0		43.7
Index 3	21.2		20.6
Index 4 and 5	32.8		35.7
**Previous experience of death**		**0.0**	
Experienced pregnancy loss	5.1		-
Never experienced pregnancy loss	94.9		-
**Childbirth weight**		**27.3**	
LBW (<2.5Kgs)	13.1		13.7
Normal Birth weight	86.9		86.3
**Childbirth order**		**1.2**	
First	45.0		45.0
2nd-4th	50.6		50.7
5th +	4.3		4.3
**Child sex**		**1.2**	
Female	50.2		50.2
Male	49.8		49.8
**Birth category**		**0.9**	
Multiple	2.4		2.4
Singleton	97.6		97.6
**Birth season in annual quarters**		**0.0**	

	**Complete case***		**Imputed**
	n=10,758		
	%ge	*missing*	*%ge*
1	25.2		-
2	24.6		-
3	26.1		-
4	24.1		-
**Birth Year**		**0.0**	
2011	21.9		-
2012	21.6		-
2013	20.5		-
2014	17.9		-
2015	18.0		-
**New-born mortality**			
Deaths with 28 days (0-27 days)	2.2**		-
Deaths within 7 days (0-6 days)	1.9**		-
Stillbirths	1.19		-
Deaths within 7 days (0-6 days) +Stillbirths	3.1		-
Late neonatal mortality (7-27 days)	0.32***		

Note: ** the denominator excludes stillbirths (128), ***the denominator excludes those who died in the first seven days of live + stillbirths
