# Supplementary material for: Mediation role of low birth weight on the factors associated with newborn mortality and the moderation role of institutional delivery in the association of low birth weight with newborn mortality in a resource-poor setting
Source: BMJ Open. 2021 May 22;11(5):e046322. doi: 10.1136/bmjopen-2020-046322 (PMC8149436; doi:10.1136/bmjopen-2020-046322)
Supplement: Supplementary data [file bmjopen-2020-046322supp002.pdf]

Supplement 2: Analyisis of Mediation effect of LBW on newborn mortality

Let y_i represents the outcome of interest as a binary variable (i) that takes the probability of the presence of outcome's exposure as P and probability of absence of the outcome's exposure as 1-P. The bivariate regression model would be of the form as in Eq. 1

$$ln\left(\frac{P_j}{1-P_j}\right) = \alpha_i + \beta_i x_i \qquad (1)$$

Where j represents categories of perinatal, late neonatal mortality, and LBW, x_i covariates of choice for each of the outcome and β_i their respective coefficients.

To assess the moderation effect of health facility delivery on the effect LBW on perinatal mortality (*pm*), an interaction term between the LBW and institutional deliver (*hf*) was introduced as indicated in Eq. 2.

$$ln\left(\frac{P_{pm}}{1-P_{pm}}\right) = \alpha_i + \beta_{lbw} x_{lbw} + \beta_{hf} x_{hf} + \beta_{lbw*hf}(x_{lbw} * x_{hf}) \qquad (2)$$

Subsequently, a multivariable regression model controlling for other factors added on eq. 2 was run (Eq. 3). In this model, I also assessed how the intersection between birth order and maternal age would affect perinatal mortality as shown in Eq. 3.

$$\begin{aligned} ln\left(\frac{P_{pm}}{1-P_{pm}}\right) = {} & \alpha_i + \beta_{lbw} x_{lbw} + \beta_{hf} x_{hf} + \beta_{lbw*hf}(x_{lbw} * x_{hf}) + \beta_{Age} x_{Age} + \beta_{educ} x_{educ} \\ & + \beta_{birth\ order} x_{birth\ order} + (\beta_{birth\ order*Age}(x_{birth\ order} * x_{Age}) \\ & + \beta_{multiple\ birth} x_{multiple\ birth} + \beta_{wealth} x_{wealth} \\ & + \beta_{nm\ expreinced\ preiviously} x_{nm\ expreinced\ preiviously} + \beta_{sex} x_{sex} \\ & + \beta_{birth\ quarter} x_{birth\ quarter} + \beta_{marital\ status} x_{marital\ \ status} \qquad (3) \end{aligned}$$

The independent variables that were included under the multivariate regression model for the late neonatal mortality (*nm*) are as indicated in Eq. 4

$$\begin{aligned} ln\left(\frac{P_{nm}}{1-P_{nm}}\right) = {} & \alpha_i + \beta_{lbw} x_{lbw} + \beta_{Age} x_{Age} + \beta_{educ} x_{educ} + \beta_{birth\ order} x_{birth\ order} \\ & + \beta_{multiple\ birth} x_{multiple\ birth} + \beta_{wealth} x_{wealth} \\ & + \beta_{nm\ expreinced\ preiviously} x_{nm\ expreinced\ preiviously} + \beta_{sex} x_{sex} \\ & + \beta_{birth\ quarter} x_{birth\ quarter} + \beta_{marital\ status} x_{marital\ \ status} \qquad (4) \end{aligned}$$

The mediating variable was selected if the predetermined endogenous – in this case, LBW was strongly associated with the main outcome variable in the multivariate model. The effect of selected covariates on LBW was determined using a multivariate regression model as indicated in Eq. 5

$$ln\left(\frac{P_{lbw}}{1-P_{lbw}}\right) = \alpha_i + \beta_{age}x_{age} + \beta_{educ}x_{educ} + \beta_{birth\ order}x_{birth\ order} + \beta_{multiple\ birth}x_{multiple\ birth} + \beta_{wealth}x_{wealth} + \beta_{nm\ expreinced\ preiviously}x_{nm\ expreinced\ preiviously} + \beta_{sex}x_{sex} + \beta_{birth\ quarter}x_{birth\ quarter} + \beta_{marital\ status}x_{marital\ status} \quad (5)$$

The indirect effect for instance for perinatal mortality with their corresponding standard errors for the factors that were significant in the LBW model were calculated as indicated in Eq. 6.

$$indirect\ effect_i = \beta_{lbw\ i} * \beta_{pm\ i} \quad (6)$$

The indirect effect's standard errors were calculated based on Goodman's approach of the product of coefficients [1] as indicated in Eq. 7.

$$\sigma_{\beta lbw\ i*\beta pm\ i} = \sqrt{\sigma^2_{lbw\ i} * \beta^2_{pm\ i} + \sigma^2_{pm\ i} * \beta^2_{lbw\ i} - \sigma^2_{lbw\ i} * \sigma^2_{pm\ i}} \quad (7)$$

The corresponding z or t -value were calculated as in Eq. 8;

$$z_i = \frac{indirect\ effect_i}{\sigma_{\beta lbw\ i*\beta pm\ i}} \quad (8)$$

Eq. 8 was used to calculate the P-values (P_i) for each indirect variable's coefficient as recommend by [2,3] in Eq. 9;

$$P_i = e^{-0.717138*z_i - 0.415973*{z_i}^2} \quad (9)$$

Reference

1 MacKinnon DP, Lockwood CM, Hoffman JM, *et al.* A comparison of methods to test mediation and other intervening variable effects. *Psychol Methods* 2002;**7**:83–104. doi:10.1037/1082-989X.7.1.83
2 Altman DG, Bland JM. Statistics notes: How to obtain the P value from a confidence interval. *BMJ* 2011;**343**:1–2. doi:10.1136/bmj.d2304
3 Altman DG, Bland JM. How to obtain the confidence interval from a P value. *BMJ* 2011;**343**:1–2. doi:10.1136/bmj.d2090
