# Supplementary material for: Mediation role of low birth weight on the factors associated with newborn mortality and the moderation role of institutional delivery in the association of low birth weight with newborn mortality in a resource-poor setting
Source: BMJ Open. 2021 May 22;11(5):e046322. doi: 10.1136/bmjopen-2020-046322 (PMC8149436; doi:10.1136/bmjopen-2020-046322)
Supplement: Author's manuscript [file bmjopen-2020-046322.draft_revisions.pdf]

BMJ Open

BMJ Open is committed to open peer review. As part of this commitment we make the peer review history of every article we publish publicly available.

When an article is published we post the peer reviewers' comments and the authors' responses online. We also post the versions of the paper that were used during peer review. These are the versions that the peer review comments apply to.

The versions of the paper that follow are the versions that were submitted during the peer review process. They are not the versions of record or the final published versions. They should not be cited or distributed as the published version of this manuscript.

BMJ Open is an open access journal and the full, final, typeset and author-corrected version of record of the manuscript is available on our site with no access controls, subscription charges or pay-per-view fees (http://bmjopen.bmj.com).

If you have any questions on BMJ Open's open peer review process please email info.bmjopen@bmj.com

BMJ Open

Association of low birth weight with new-born mortality in a resource-poor setting: a pathway analysis of event history demographic data

Journal:	*BMJ Open*
Manuscript ID	bmjopen-2020-046322
Article Type:	Original research
Date Submitted by the Author:	28-Oct-2020
Complete List of Authors:	Kananura, Rornald; London School of Economics and Political Science, International Development
Keywords:	SOCIAL MEDICINE, Community child health < PAEDIATRICS, EPIDEMIOLOGY, PUBLIC HEALTH

SCHOLARONE™
Manuscripts

BMJ

I, the Submitting Author has the right to grant and does grant on behalf of all authors of the Work (as defined in the below author licence), an exclusive licence and/or a non-exclusive licence for contributions from authors who are: i) UK Crown employees; ii) where BMJ has agreed a CC-BY licence shall apply, and/or iii) in accordance with the terms applicable for US Federal Government officers or employees acting as part of their official duties; on a worldwide, perpetual, irrevocable, royalty-free basis to BMJ Publishing Group Ltd ("BMJ") its licensees and where the relevant Journal is co-owned by BMJ to the co-owners of the Journal, to publish the Work in this journal and any other BMJ products and to exploit all rights, as set out in our licence.

The Submitting Author accepts and understands that any supply made under these terms is made by BMJ to the Submitting Author unless you are acting as an employee on behalf of your employer or a postgraduate student of an affiliated institution which is paying any applicable article publishing charge ("APC") for Open Access articles. Where the Submitting Author wishes to make the Work available on an Open Access basis (and intends to pay the relevant APC), the terms of reuse of such Open Access shall be governed by a Creative Commons licence – details of these licences and which Creative Commons licence will apply to this Work are set out in our licence referred to above.

Other than as permitted in any relevant BMJ Author's Self Archiving Policies, I confirm this Work has not been accepted for publication elsewhere, is not being considered for publication elsewhere and does not duplicate material already published. I confirm all authors consent to publication of this Work and authorise the granting of this licence.

For peer review only

Association of low birth weight with new-born mortality in a resource-poor setting: a pathway analysis of event history demographic data

Rornald Muhumuza Kananura [1, 2]

1. London School of Economics and Political Science, Department of International Development, Houghton St, London, WC2A 2AE
2. Makerere University School of Public Health, Department of Health Policy Planning and Management, New Mulago Complex, Kampala, Uganda

Corresponding author

Rornald Muhumuza Kananura, r.m.kananura@lse.ac.uk, +447985274523

For peer review only

For peer review only - http://bmjopen.bmj.com/site/about/guidelines.xhtml

Abstract

Objectives

To assess the mediation effect of Low Birth Weight (LBW) on New-born Mortality (NM) and the role of institutional delivery in their association.

Design and participants

I used the 2011-2015 event histories health demographic data collected by Iganga-Mayuge Health Demographic and Surveillance Site (HDSS). The dataset consisted of 10,758 registered women whose birth occurred at least 22 weeks of the gestation period and records of new-borns' living status within 28 days after birth.

Setting

The Iganga-Mayuge HDSS data is located eastern Uganda and it routinely collects data on pregnancies, births, migrations, and mortality from a registered population of at least 90,000 people.

Outcome measure

The study's key outcomes or endogenous factors that were Perinatal Mortality (PM), Late NM, and LBW (mediating factor).

Results

As of 2015, LBW, PM and NM were estimated at 13%, 32 per 1000 total births and 19 per 1000 live births respectively. Of the PM, death within 24 hours including stillbirth accounted for 83%. Similarly, 64% of the NM occurred within 24 hours. LBW was positively associated with perinatal mortality. The factors that were indirectly associated with perinatal mortality via LBW were adolescence age, rural residence, mothers' previous experience of NM, multiple births, and marital status. Multiple births and mother's prior experience of NM were positively associated with NM at a later age. Institutional delivery had a modest inverse role in the association of LBW with NM.

For peer review only - http://bmjopen.bmj.com/site/about/guidelines.xhtml

Conclusion

LBW has a strong mediating role in the NM pathways. Prevention interventions should target adolescents, uneducated and rural residents as the risk groups in addition to LBW new-borns' management measures. The findings demonstrate the need for a holistic life-course approach that gears the health systems to tackle NM from the onset of conception.

Keywords: *new-born health, Low birth weight, health systems, Sub-Saharan Africa, Uganda*

Strengths and limitations

- The study uses a large event history health demographic data that has never been exploited to study perinatal and new-born health.
- The gestation age that could have provided clear categories of prematurity and stillbirths' categories was not documented during birth registration.
- There was no information on the maternal morbidities, and complications that lead to different birth outcomes, which could have been used to understand their effect along the path.
- There was a considerable proportion of missingness for some of the variables which we addressed through multiple imputations to minimise the missingness bias.

For peer review only - http://bmjopen.bmj.com/site/about/guidelines.xhtml

Word count: 5202

Introduction

The evidence is clear that the availability of maternal and newborn life-saving technologies and effective interventions have worked towards reducing newborn mortality in developed countries and other developing countries (Darmstadt et al. 2005; Dickson et al. 2014; Zupan 2005). However, the newborn mortality rate shares the largest portion of under-five mortality and has remained unacceptably high in Sub-Saharan Africa (SSA). Of the 2.5 million new-born death that occurred in 2018, 41% is a portion of SSA [4]. The neonatal mortality rate (NMR) in SSA has persistently remained unacceptably high at an average of 27-28 per 1000 live births [4–6]. At least 75% of the new-born mortality occurs within the first week of life [7], of which close to 55% happen within 24 hours [7]. These rates could be underestimated as stillbirths are normally misclassified because of weak documentation systems in SSA [8].

The high proportion of new-born mortality has been attributed to Low Birth Weight (LBW) [9,10] and their related consequences [11]. The direct effect of the socio-economic and demographic factors such as wealth, education level, and maternal age on newborn mortality that has been indicated in the framework by [12,13] have been widely studied, but evidence of how these could be mediated by LBW is elusive. For instance, a direct effect of adolescence age and advanced maternal age on neonatal mortality has been observed in some of the studies [14–17] however, available evidence indicates that extremes of maternal age are associated with the increased risk of LBW and other adverse complications [18–24] that could ultimately lead to new-born mortality. Regarding socio-economic factors, the association of educated and wealthy households or women with neonatal mortality [25,26] could be through other mechanisms [26,27] such as delayed age at marriage, sexual debut, better economic opportunities for women and better behavioural practices. Additionally, women from wealthy

For peer review only

families or households are more likely to access LBW health control interventions [26,28–30] and can afford to incur an extra bill, aside to what is freely provided by the government [31,32]. Other LBW contributors include infections (such as syphilis, malaria), pre-pregnancy and pregnancy malnutrition exposure and exposure to environmental factors particularly indoor air pollution, and tobacco and drug use [9,20,33,34]. The scarcity of evidence on how LBW mediates newborn mortality limits the design of moderating interventions. The simultaneity analytical approach of understanding the mediating role of LBW in the association of newborn mortality with socio-economic, demographic and other individual factors may contribute to generating both clinical and community-based interventions or policies that focus on the health of women before and after pregnancy; and after delivery.

Further, access to health services is known as the most effective intervention in preventing maternal morbidities and controlling LBW and other complications' consequences (Darmstadt et al. 2005; Dickson et al. 2014). Attending the recommended number of antenatal care exposes the mother to the interventions for identifying and managing pregnancies [35] that may lead to LBW. Additionally, access to ANC exposes the mother-foetus dyad to preventions and care interventions that reduce the occurrences of maternal morbidities such as syphilis and malaria that are associated with the high likelihood of LBW and prematurity. As earlier mentioned, the first day of life is important for the survival of new-born and thus, access to quality of care within labour or delivery time is crucial. During labour, women should be attended to by skilled health workers, who should be able to assess the labour complications such as obstructed labour conditions that may need emergency services such as caesarean-section (Campbell 2014; Darmstadt et al. 2013; Salam et al. 2014; Wall et al. 2010). After delivery, the new-borns should be screened for life-threatening signs such as LBW and asphyxia to be able to benefit from health interventions such as resuscitation for asphyxia and corticosteroid treatment administration or kangaroo mother care for LBW (Berkley et al. 2014; Darmstadt et al. 2005;

For peer review only - http://bmjopen.bmj.com/site/about/guidelines.xhtml

Dickson et al. 2014; Salam et al. 2014). Noteworthy, reaching the health facilities in the SSA context does not guarantee access to the required services [26,42] because of numerous bottlenecks that impend access to required services. The required health facility-based interventions could be performed inappropriately or too late or women and new-borns may fail to access the required interventions at all because of inadequacies in supplies and skilled health workers [3,43]. With the high increasing rates of low birth weight and preterm birth vis-à-vis the stagnation in high neonatal mortality rates in SSA despite the increase in health facility delivery, evidence on how the health facility deliveries moderates the survival of LBW newborns is warranted.

The above discussion highlights the role of LBW in mediating the association of some of the factors with new-born mortality and the role of institutional delivery in the relationship between LBW and new-born mortality. However, the pathways and mechanisms of the interactions are unclear, particularly in the sub-Sharan African context. In this study, therefore, I analyse the event history demographic data that is collected by the Iganga-Mayuge Health and Demographic Surveillance Site (HDSS) to assess how LBW mediates new-born mortality and the role of institutional delivery in the association of LBW and new-born mortality. These data have not been exploited in studying newborn survival in Uganda and other countries that share the same context.

For peer review only

Methods

Data

This study uses the 2011-2015 Event Histories (EH) health demographic data collected by Iganga-Mayuge Health Demographic and Surveillance Site (HDSS). The EH dataset consisted of 10,758 registered women whose birth occurred at least 22 weeks of the gestation period and records of new-borns' living status within 28 days after birth. The HDSS data are collected in the eastern region of Uganda covering 65 villages in seven sub-counties, 16 health centres and one hospital in Iganga and Mayuge districts. The HDSS routinely collects data on pregnancies,

births, migrations, and mortality from a registered population of at least 90,000 people. Community health workers who are based at a village level notify and report all events and thereafter, the HDSS Field Assistants follow-up all the reported events for actual documentation using standard tools. The analysis is limited to births among residents, in-migrants that had stayed in the HDSS for at least 6 months and out-migration that had stayed outside the HDSS within 6 months. The definition of an HDSS resident is someone who has stayed within the HDSS for at least 6 months and out-migrants remain the HDSS residents within 6 months of out-migration. To date, such large event history data in a resource-poor setting have not been used for this kind of studies.

Study variables

The study's key outcomes or endogenous factors that were used to describe the perinatal and new-born mortality or survival system are perinatal mortality, late neonatal mortality and LBW (mediating factor). Perinatal mortality includes deaths within 7 days after birth and all stillbirths, while late neonatal mortality is defined as death within 21 days after the first week of survival (7-28 days)[44,45] – describing the timing of death within the new-born survival system. LBW – defined as a birth weight that is less than 2.5 Kgs at birth[46] explains how new-born survival could be affected indirectly by other factors through birth weight.

The new-born adverse outcomes' exogenous health system variables were categorised as maternal and household factors (marital status, education level, maternal age, wealth index), child individual risk factors (childbirth order, birth category – multiple or singleton, child's sex), institutional delivery, mother's previous experience of new-born loss or stillbirth, place of residence (rural or urban), and birth season (in annual quarters). Marital status was categorised as a dummy variable for 1 – having a partner (those who were either married or cohabiting) and 0 – having not a partner (those who were single or widows). The wealth indices 3-4 were considered as less poor and 1-2 were considered as poorer. The wealth index was

For peer review only - http://bmjopen.bmj.com/site/about/guidelines.xhtml

generated using principal component analysis and the items were household assets, household roof structure, household floor structure, and household wall structure. Birth order was categorised as 1 – first order, 2 – 2nd to 4th order and 3 – 5th order. Education was categorised as no education attained, primary level attained, and the post-primary level attained; however, education level of at least primary was related to the reduced risks of perinatal and late neonatal mortality, so I later made it as a single dummy variable of no education (0 – at least primary level attained and 1 – no education level at all). Maternal age was grouped as <20 years, 20-29 years and 30 years and above based on the non-parametric analysis (Supplement 1).

Data cleaning and organization

Data analysis and cleaning such as categorisation and removing of duplicates were done in STATA v.15. An initial examination of the relationship between the key study independent variables and study outcomes was performed. Non-parametric generalised additive modelling on outcome variables and maternal age relationship was also done to guide the variable classification (Supplement 1) on the assumption of having a non-linear relationship between age and study outcomes.

Multicollinearity between the independent variables was considered at the cut-off point of Variance Inflation Factor (VIF) <10 [47] and there was no evidence of potential multicollinearity as each variable's VIF was less than 6 (Supplement 1). However, because of strong correction between the multiple birth and LBW, multiple birth was dropped under the perinatal mortality multivariable model, while LBW was dropped under the late neonatal mortality multivariable model. Because of the homogeneity across the study clusters such as villages and sub-counties within the HDSS, the application of clustering or multilevel modelling approach could not yield a variation across these clusters. Nonetheless, the use of fixed-effect approach with the inclusion of variables such as place of residence (urban or rural)

For peer review only

and household index as key variables of interest provides an insight into the variation in new-born mortality across the place of residence and household in terms of wealth status.

Sensitivity analysis

The extent and pattern of missing data were scrutinised to guide the modelling strategy. Birth weight was missing among 27%, 19% for wealth index, and <1% for other variables. To check if the missingness status was missing completely at random or missing at random, I ran a model of missing dummies (0 – not missing and 1 – missing) controlling for variables that I expected to increase the probability of missingness. For birth weight, I found that the likelihood of missing was highly associated with those who did not deliver in the health facilities and those who were born as stillbirths or died immediately after birth (Supplement 1) – indicating a possibility of birth weight missing at random. Similarly, the likelihood of wealth index was highly associated with the maternal age of less than 20 years (Supplement 1) – indicating a possibility of wealth index missing at random. Given the determinants of missingness, I ran multiple imputations (m=100) not only controlling for identified factors that contribute to the likelihood of missingness, but also all other variables (as auxiliary) in the dataset. The multiple imputation method has been indicated as an important approach for minimising the missingness bias [48] and this does not depend on the magnitude of missingness [49,50]. After imputation, LBW was found to be underestimated for completed cases data, while the household's poor status was found to be overestimated for completed cases data (Supplement 1).

Additionally, considering newborn mortality as a rare event, I ran a model for rare events known as firth logistic model [51] to assess the sample size bias for perinatal mortality, late neonatal mortality, and LBW coefficient estimates. Comparing the firth logit model results with the logistic regression model, I found that the results were consistent (Supplement 1). I

For peer review only - http://bmjopen.bmj.com/site/about/guidelines.xhtml

later decided to use the logistic modelling because the less time it takes while running imputed data contrary to firth logit.

Statistical modelling

The outcome variables (LBW, prenatal mortality, late neonatal mortality) were in the form of binary with 1 – indicating the presence of outcome exposure and 0 – the absence of outcome exposure. I, therefore, used a generalised linear modelling approach with a logit link function. To examine the newborn mortality pathway, several analysis steps were applied. Bivariate model(s) with all covariates on the perinatal, late mortality and LBW as outcome variables were performed to identify the variables that would significantly affect these outcomes of interest in the adjusted models. Details on the analysis of the mediation effect of LBW on newborn mortality are indicated in the Supplement 2

Patient and public involvement

This study uses anonymised secondary data that were collected by Iganga-Mayuge Health and Demographic Surveillance Site (HDSS) in Eastern Uganda and thus, it does not directly involve human participants.

Results

Participants' characteristics

Of the total registered births between 2011-2015, 64% were rural residents (Supplement 3). The average maternal age was 27.3 years (standard deviation=±6.5) and 14% were adolescent mothers. Overall, 84% of deliveries occurred in health facilities of which 15% occurred in private health facilities (Supplement 3). Regarding maternal socio-economic, 86% of the mothers had attained at least primary level education, 53% belonged to a wealth index of 3-5 and 86% were staying with a partner or married (Supplement 3).

For peer review only - http://bmjopen.bmj.com/site/about/guidelines.xhtml

Perinatal mortality, neonatal mortality, and LBW estimates

The overall perinatal mortality rate and stillbirth rate within 7 days after delivery (0-6) in the 5 years preceding 2016 were 31 and 12 per 1000 total births respectively (Supplement 1). Of the total prenatal mortality cases, death within 24 hours (0-1 days + stillbirths) accounted for 83% (Figure 1). Similarly, the overall neonatal mortality rate in the 5 years preceding 2016 was 22 per 1000 live births. Of the neonatal mortality cases, 62% were deaths within 24 hours of life (0-1 day) and 14 per 1000 live births were deaths within 7-27 days (Figure 1).

Comparing the neonatal and perinatal mortality across five years of birth and outcome registration, we see a slight change in perinatal mortality curve between 2012-2014 with mortality rate reducing from 31-28 per 1000 total birth and gaining its position in the subsequent years (Figure 2). Similarly, slight changes in neonatal mortality rates were observed between 2013 and 2015 with the rates increasing by 3 units between 2013-2014 and reducing by 6 units in the subsequent year (Supplement 1). With regards to the LBW, we observe a linear trend between 2011-2012 that later reduced by 3 units in the subsequent year – remaining uniform for the rest of the reporting years (Figure 2).

Perinatal mortality, neonatal mortality, and LBW distribution by social-economic and demographic characteristics

I examined the distribution of the study's LBW, perinatal and neonatal mortality by key socio-economic and demographic characteristics. LBW was higher among adolescent aged women as compared to those aged 20 years and above, unmarried women, and rural area residents (Figure 3). Perinatal mortality was higher among those who delivered in the community and public health facilities. For neonatal mortality, a slightly lower death among married, urban residents and deliveries in the private health facilities was observed (Figure 3). Additionally, LBW and neonatal mortality were observed to be higher among those whose mothers had ever experienced pregnancy loss and those born as multiple births (Figure 4). The

For peer review only - http://bmjopen.bmj.com/site/about/guidelines.xhtml

For peer review only

previous experience of pregnancy loss was also associated with increased cases of perinatal mortality (Figure 4). The potential factors for perinatal mortality, late neonatal mortality and LBW are investigated in the subsequent sections.

The perinatal mortality pathways

To describe the perinatal mortality pathway, I provide the results in two tables (Table 1, Table 2 and Table 3). Table 1 indicates how the LBW effect changed with institutional delivery and the inclusion of other factors. Table 2 indicates the indirect factors that affected perinatal mortality via LBW. Table 3 indicates the total indirect effect. At bivariable level, the crude odds of perinatal mortality were slightly higher for facility deliveries relative to those conducted in the community (e^{β}=1.01, P>0.05) (Table 1). Also, at bivariable level, the crude odds of perinatal mortality were higher among LBW babies relative to those weighing 2.5Kgs+ (e^{β}=2.54, P<0.001). Controlling for health facility delivery and introducing an interaction between LBW, a modest effect of health facility delivery on the effect of LBW was observed (e^{β}=0.91, P>0.10) (Table 1). Controlling for other factors in the final model, the odds of perinatal mortality among the LBW new-borns remained almost the same (e^{β}=2.55, P<0.05). The other factors that were directly associated with the increased odds of perinatal mortality were being a rural resident (e^{β}=1.38, P<0.05), mother's previous experience of perinatal mortality(e^{β} =3.95, P<0.001), maternal advanced age (e^{β}=1.68, P<0.01) and having no education at all (e^{β} =1.68, P<0.001). Birth of 5th order and above was inversely associated with perinatal mortality (e^{β}=0.38, P<0.05) (Table 1).

For peer review only - http://bmjopen.bmj.com/site/about/guidelines.xhtml

Table 1: Underlying determinants of perinatal mortality using 2011-2015 Iganga-Mayuge HDSS event histories data

	Unadjusted			Adjusted 1			Adjusted 2			Fully adjusted		
	**Coef.**	**Se**	**P-value**	**Coef.**	**Se**	**P-value**	**Coef.**	**Se**	**P**	**Coef.**	**Se**	**P-value**
**Childbirth weight**												
<2.5 Kgs	-	-	-	-	-	-	-	-	-	-	-	-
25 Kgs+	0.93	0.19	<0.001	0.93	0.19	<0.001	1.00	0.41	0.02	0.94	0.41	0.02
**Place of delivery**												
Community	-	-	-	-	-	-	-	-	-	-	-	-
Health facility	0.01	0.12	0.91	0.04	0.12	0.75	0.06	0.13	0.68	0.15	0.14	0.28
**Place of delivery and LBW interaction**												
<2.5 Kgs and health facility delivery interaction	-	-	-	-	-	-	-0.10	0.42	0.82	-0.10	0.42	0.82
**Annual birth quarters**												
1	-	-	-	-	-	-	-	-	-	-	-	-
2	0.08	0.16	0.63	-	-	-	-	-	-	0.07	0.17	0.67
3	0.23	0.16	0.14	-	-	-	-	-	-	0.20	0.16	0.20
4	0.18	0.16	0.27	-	-	-	-	-	-	0.18	0.16	0.27
**Maternal education level**												
Primary+	-	-	-	-	-	-	-	-	-	-	-	-
None	0.52	0.15	<0.001	-	-	-	-	-	-	0.49	0.15	<0.001
**Maternal age**												
<20	0.12	0.16	0.46	-	-	-	-	-	-	0.08	0.39	0.84
20—29	-	-	-	-	-	-	-	-	-	-	-	-
30+	0.39	0.11	<0.001	-	-	-	-	-	-	0.52	0.17	<0.001
**Childbirth order**												
1	-0.23	0.12	0.06	-	-	-	-	-	-	0.05	0.22	0.82
2—4	-	-	-	-	-	-	-	-	-			
5th+	-0.67	0.41	0.10	-	-	-	-	-	-	-0.96	0.42	0.02
**Place of residence**												
Urban	-	-	-	-	-	-	-	-	-			

For peer review only

For peer review only - http://bmjopen.bmj.com/site/about/guidelines.xhtml

	Unadjusted			Adjusted 1			Adjusted 2			Fully adjusted		
	**Coef.**	**Se**	**P-value**	**Coef.**	**Se**	**P-value**	**Coef.**	**Se**	**P**	**Coef.**	**Se**	**P-value**
Rural	0.33	0.12	0.01	-	-	-	-	-	-	0.32	0.15	0.04
**Marital status**												
No partner	-	-	-	-	-	-	-	-	-	-	-	-
Has a partner	-0.30	0.16	0.07	-	-	-	-	-	-	-0.29	0.18	0.11
**Experienced neonatal or pregnancy loss previously**												
No	-	-	-	-	-	-	-	-	-	-	-	-
Yes	1.43	0.15	<0.001	-	-	-	-	-	-	1.37	0.16	<0.001
**Household Wealth**												
Poor(er) (1-2)	-	-	-	-	-	-	-	-	-	-	-	-
Less poor (3-5)	0.10	0.11	0.40	-	-	-	-	-	-	-0.16	0.14	0.25
**Child sex**												
Male	-	-	-	-	-	-	-	-	-	-	-	-
Female	0.07	0.14	0.61	-	-	-	-	-	-	0.05	0.14	0.73
**First birth order-adolescence age interaction**												
First order and <20 years	-	-	-	-	-	-	-	-	-	0.17	0.45	0.71
**First birth order-advanced age interaction**										-	-	-
First order and 30+ years	-	-	-	-	-	-	-	-	-	-0.31	0.36	0.40
**Birth category**												
Singleton	-	-	-	-	-	-	-	-	-	-	-	-
Multiple	0.02	0.36	0.95	-	-	-	-	-	-	-	-	-

For peer review only

For peer review only - http://bmjopen.bmj.com/site/about/guidelines.xhtml

As mentioned earlier, the fact that LBW was strongly associated with perinatal mortality, it was considered as a potential mediating factor for perinatal mortality. Table 2 indicates the magnitude effect of the LBW factors in the study's dataset. The factors that were associated with the increased odds of LBW were adolescent mothers (e^{β}=1.39, P<0.001), rural resident mothers (e^{β}=1.40, P<0.001), mothers with previous experience of new-born or pregnancy loss (e^{β}=1.33, P=0.05), babies born as multiple births(e^{β}=3.12, P<0.001). Having a partner or being married was inversely associated with LBW (e^{β}=0.76, P<0.05) (Table 2). The indirect effect of these variables on perinatal mortality is summarised in (Supplement 1).

Table 2: Perinatal mortality indirect factors mediated through LBW using Iganga-Muyuge HDSS event history data

	Unadjusted			Adjusted		
	**Coef.**	**Se**	**P-value**	**Coef.**	**Se**	**P-value**
**Annual birth quarters**						
1	-	-	-	-	-	-
2	0.11	0.09	0.25	0.11	0.09	0.23
3	0.05	0.09	0.59	0.06	0.10	0.55
4	0.03	0.09	0.76	0.03	0.10	0.76
**Maternal education level**						
Primary+	-	-	-	-	-	-
None	0.13	0.09	0.16	0.13	0.10	0.18
**Maternal age**						
<20	0.36	0.09	<0.001	0.33	0.10	<0.001
20—29	-	-	-	-	-	-
30+	-0.06	0.07	0.39	-0.02	0.08	0.80
**Childbirth order**						
1	-0.09	0.06	0.16	-0.13	0.08	0.09
2—4	-	-	-	-	-	-
5th+	0.03	0.16	0.87	-0.03	0.17	0.85
**Place of residence**						
Urban	-	-	-	-	-	-
Rural	0.33	0.07	<0.001	0.34	0.08	<0.001
**Marital status**						
No partner	-	-	-	-	-	-
Has a partner	-0.34	0.09	<0.001	-0.28	0.10	0.01
**Experienced neonatal or pregnancy loss previously**						
No	-	-	-	-	-	-
Yes	0.37	0.14	0.01	0.29	0.14	0.05
**Birth category**						

For peer review only - http://bmjopen.bmj.com/site/about/guidelines.xhtml

For peer review only

	Unadjusted			Adjusted		
	Coef.	Se	P-value	Coef.	Se	P-value
Singleton	-	-	-	-	-	-
Multiple	1.13	0.15	<0.001	1.14	0.15	<0.001
**Household Wealth**						
Poor(er) (1-2)	-	-	-	-	-	-
Less poor (3-5)	0.15	0.07	0.03	-0.08	0.08	0.32
**Child sex**						
Male	-	-	-	-	-	-
Female	0.12	0.07	0.06	0.12	0.07	0.08

The increase in the odds of late neonatal mortality was associated with previous experience of pregnancy loss or neonatal mortality (e^{β}=3.17, P<0.05) and multiple births (e^{β} =6.92, P<0.001) (Table 3).

Table 3: Late neonatal mortality risk factors using 2011-2015 Iganga-Mayuge HDSS event histories data

	Unadjusted			Adjusted		
	Coef.	Se	P-value	Coef.	Se	P-value
**Annual birth quarters**						
1	-	-	-	-	-	-
2	0.44	0.53	0.41	0.50	0.53	0.34
3	0.49	0.52	0.35	0.50	0.52	0.34
4	0.36	0.54	0.51	0.34	0.54	0.53
**Education Level**						
No level of education	-	-	-	-	-	-
Primary education+	0.31	0.45	0.49	0.22	0.46	0.63
**Maternal age (years)**						
<20	-0.13	0.53	0.80	0.19	0.61	0.76
20-29	-	-	-	-	-	-
30+	0.55	0.35	0.12	0.55	0.39	0.16
**Childbirth order**						
1st	-0.36	0.36	0.32	-0.13	0.43	0.76
2—4	-	-	-	-	-	-
5th+	0.79	0.61	0.20	0.64	0.64	0.32
**Place of residence**						
Urban	-	-	-	-	-	-
Rural	-0.27	0.35	0.45	-0.20	0.44	0.65
**Marital status**						
Has no partner	-	-	-	-	-	-
Has a partner	-0.41	0.46	0.37	-0.61	0.49	0.22
**Experienced neonatal or pregnancy loss previously**						
No	-	-	-	-	-	-
Yes	1.28	0.49	0.01	1.16	0.52	0.03

For peer review only - http://bmjopen.bmj.com/site/about/guidelines.xhtml

For peer review only

	Unadjusted			Adjusted		
	**Coef.**	**Se**	**P-value**	**Coef.**	**Se**	**P-value**
**Birth category**						
Singleton	-	-	-	-	-	-
Multiple	2.04	0.49	<0.001	1.94	0.50	<0.001
**Household Wealth**						
Poorer (1-2 index)	-	-	-	-	-	-
Less poor (3-5 index)	-0.29	0.36	0.43	-0.33	0.45	0.46
**Child sex**						
Male	-	-	-	-	-	-
Female	-0.57	0.36	0.12	-0.60	0.36	0.10
**Place of delivery**						
Community	-	-	-	-	-	-
Health facility	0.20	0.39	0.61	-	-	-
**Birth weight***						
Birth weight>=2.5 Kg	-	-	-	-	-	-
Low birth weight	.83	0.45	0.06	-	-	-

Note: *Could not be included because of its strong relationship with multiple births*

Discussion

To the best of my knowledge, this is the first study in a resource-poor setting that seeks to understand how LBW newborn mediates the new-born mortality and how the institution delivery may moderate the LBW effect on new-born survival. The analysis was based on an event history health demographic dataset that has not been exploited in studying the newborn survival in Uganda and other countries with the same context. The study results provide an insight into new-born survival life-course and health systems interventions, particularly targeting the prevention of LBW occurrences and management of or care for LBW new-borns. In the subsequent sub-sections, I discuss 1) the health facility pathways in affecting the survival of new-borns, and 2) the pathway through which LBW mediate the new-born survival. The results are interpreted and where possible recommendations for the design of appropriate interventions are discussed based on the available studies' and implementation research evidence in a similar context.

Health facility delivery and neonatal mortality

For peer review only - http://bmjopen.bmj.com/site/about/guidelines.xhtml

For peer review only

The study results confirm the persistent high perinatal mortality rates in Uganda with stillbirths and death within 24 hours contributing the largest share. The modest effect of health facility delivery on the survival of LBW new-borns and the large proportion of new-born death within 24 hours after birth indicates a gap in accessing the required health services. We know that, during labour, women should be attended to by skilled health workers who are knowledgeable and skilled to screen for labour complications such as obstructed labour conditions, which need lifesaving emergency services for instance caesarean section (Darmstadt et al. 2013; Das et al. 2014). However, access to these interventions is in most cases limited in resource-limited setting [3]. Further, the substantial effect of LBW on the survival for new-borns is well documented (Barros et al. 2011; Blencowe et al. 2012), but interventions for preventing or controlling the effect of LBW are always inadequately available in such resource-limited settings. For instance, kangaroo mother care and resuscitation that are easy to implement, inexpensive and effective [1,52] for helping such new-borns survival are not universally implemented in most of the health facilities in such settings. Additionally, access to ANC such as screening for maternal morbidities and prenatal interventions may help in protecting the mother-foetus dyad from maternal morbidities [53] and ultimately reducing the likelihood of adverse outcomes, but usually, most women do not access such services.

Noteworthy is that in this study's setting and sub-Saharan Africa in general, such interventions may fail to work effectively because of community contextual problem in particular poor transport and referral systems, which contribute to delays and failures in receiving required services. For instance, in this study setting, there is one public hospital (Iganga Hospital) that provides Comprehensive Emergency Obstetric and New-born Care services in the district [54] and because of the long-distance coupled with poor transport and referral systems, in most cases, women from rural or remote areas fail to access the services and for those that make it reach when they are in advanced stages of complications. Such could

For peer review only - http://bmjopen.bmj.com/site/about/guidelines.xhtml

perhaps explain why in this study the rural residents were associated with the high risk of perinatal mortality.

Further, in such settings, most women first access services at different care points within the community and are later referred or go to the accredited or higher-level facilities when adverse complication have emerged. Thus, most women that deliver in higher-level facilities in most cases have complications that are associated with a high likelihood of perinatal mortality. These highlighted challenges could perhaps explain why health facility delivery could not contribute substantially to the reduced likelihood of stillbirth and death within 24 hours as these are in most cases done too late. Additionally, the SSA's rural communities are sometimes characterised by community cultural and social behaviours that affect access to pregnancy, delivery and new-born care interventions that may later contribute to the high likelihood of adverse birth outcomes such as LBW as this study confirms. These findings suggest interventions that improve healthcare access coverage and quality of care simultaneously if the facility interventions are to match better outcomes.

LBW effect on perinatal and neonatal mortality

LBW was identified as a potential determinant of perinatal mortality. The other determinants of new-born mortality were marital status, maternal age, and maternal education. Having a partner was inversely associated with LBW, which was consistent with another study on the effect of marital status on the birth outcome [55] – implying that it may reduce perinatal mortality indirectly through birth weight. The effect of marital status on child health outcome could be via decision-making power, which could influence the choice of appropriate services. In the study's context and sub-Saharan Africa in general, men usually possess resources and assets; and thus, unmarried or single women in such settings are less likely to have access to better care [56]. Thus, in addition to the negotiating power with the health workers, married women have support from their spouse in terms of joint income, joint care and joint decision-

For peer review only

making, which may contribute to their birth preparedness [57], child nutrition and general health of the household. In addition, the health policy or rule in Uganda that require women to attend maternal services in accompany of their spouses may limit the unmarried counterpart from accessing maternal health facility services [58–60] that would address LBW. Studies done in similar settings have indicated that women would be denied services unless they attended with their husbands and sometimes those with husbands would be attended to as a priority [58–60]. While male engagement is a positive factor, it has been found that it could end up being a discriminative strategy that could create stigma among unmarried women, inhibiting them from accessing the required services [59,60]. Inclusive interventions that target all groups of women regardless of statuses such as age and marital status should be designed.

Maternal age of 30 years+ was positively associated with perinatal mortality as the adolescence age (15-19 years) was positively associated with LBW – indicating how the tail ends of maternal age are associated with the increased likelihood of new-born mortality. Notwithstanding, the effect of maternal age on perinatal mortality needs to be interpreted with care because of other confounding factors such as birth order or parity. On the first hand, the effect of maternal age of 30 years+ could be related to birth order. In this study, new-borns of birth order of at least 5 were at lower risk of mortality compared to the first birth order – a finding that is consistent with a study done in India and Bangladesh [61] and when an interaction between maternal age and birth order was introduced, the first births among women aged at least 30 years were positively associated with perinatal mortality. On the other hand, we also know that high parity is associated with pregnancy complications such as hypertension in pregnancy that may lead to the high likelihood of adverse birth outcomes. In low and middle-income countries where maternal age at the first delivery begins as early as 10 years [62], by the age of 25-30 years most women have experienced at least 4 number of pregnancies that would increase the risk of hypertension and ultimately adverse birth outcomes. In this study,

For peer review only - http://bmjopen.bmj.com/site/about/guidelines.xhtml

28% and 49% of women aged 20-29 and 30 years respectively had children of 3rd and 4th birth order. These results suggest for adolescence pregnancy and fertility control interventions, and for the pregnant women, improved screening for morbidity in pregnancy such as hypertension and early access to interventions that would reduce growth restriction and preterm births are emphasised.

In this study, multiple births were not strongly associated with perinatal mortality but strongly associated with mortality at a later age. Although some studies have indicated multiple births to be highly associated with infant and under-five mortality [63,64], this study highlights that the association may vary by age. The association of multiple births with perinatal mortality in this study has been indicated to be moderated by LBW. The association of multiple births with LBW and preterm births has been also studied in other studies [65,66]. Women with multiple births need special care [67,68] in terms of nutrition to be able to produce enough breast milk to feed the new-borns. Further, the fact the multiple new-borns are more likely to be very small new-borns, appropriate regular postnatal examinations are recommended [65]. However, for marginalised communities and families, such care could be inadequate because of limited resources. In addition, multiple birth children are in most cases preterm or very LBW new-born and thus susceptible to recurrent infections that may need frequent health care services, which are often inadequately available in rural communities. Interventions within community and health facility levels for supporting women with multiple births are thus needed. I also recommend a comprehensive study on the care for multiple births including small new-borns beyond the facility interventions, particularly focusing on the burden of caring for such new-borns in a resource-limited setting.

Attending at least primary education level was directly associated with the reduced likelihood of perinatal mortality – a finding that has been indicated in another study [69]. The pathway could be better health behavioural practices such as nutrition, better sanitation, and

For peer review only - http://bmjopen.bmj.com/site/about/guidelines.xhtml

appropriate healthcare access. In most cases, educated women have access to the health information on maternal nutrition, good hygiene practices and maternal danger signs that could have been attained either while at school or through reading media publications. These findings suggest a need to design and implement behavioural interventions that integrate less educated communities. One of the strategies that have been effective in mobilizing and sensitizing poor and illiterate communities in such resources-limited settings is the use of community health workers, which could be exploited to target such groups with maternal and newborn specific health information. Additionally, interventions that promote girl child education and those that prevent early pregnancies targeting girls and families could contribute to increasing the level of education among women in the long run.

Experience of previous stillbirth and neonatal death was related to a high likelihood of perinatal mortality and continued to affect the neonates in the later stage, which is consistent with other studies done in developed countries [70,71]. Women with previous experience of pregnancy loss are susceptible to risk conditions such as preeclampsia, preterm births, intrauterine growth retardation and foetal distress that could affect the health of subsequent pregnancies [72]. Additionally, the recurrent of perinatal mortality particularly stillbirths could be related to maternal chronic conditions [71]. Proper screening for the histories of pregnancy loss and neonatal mortality in addition to the histories of related risk factors such as hypertension, diabetes and epilepsy should be emphasised during prenatal and labour period.

Study strengths and limitations

The study uses a large event history health demographic data that has never been exploited to study perinatal and new-born health. The health and demographic surveillance data have been indicated to have a high statistical power that is close to 100% for mortality measurement [73]. Nevertheless, this study has some limitations related to the data source and context. First, the gestation age that could have provided clear categories of prematurity and

For peer review only - http://bmjopen.bmj.com/site/about/guidelines.xhtml

stillbirths' categories was not documented during birth registration. Second, there was no information on the maternal morbidities, and complications that lead to different birth outcomes, which could have been used to understand their effect along the path. Third, there was a considerable proportion of missingness for some of the variables that could lead to bias, however, multiple imputations approach was performed to minimise the missingness bias. Lastly, the study results are generalisable to eastern Uganda setting, nonetheless the fact that most of the communities not only in Uganda but also other Sub-Saharan Africa share the same community and health characteristics, such results could guide the design of health systems interventions that address both mortality causes and risk factors.

Conclusion

This manuscript contributes to the understanding of child mortality by analysing the new-born mortality pathways in a resource-poor setting. The study affirms the role of LBW in mediating the association of socio-economic and demographic factors with new-born mortality. In addition, the study has identified the continued effect of multiple birth and mother's previous experiences of pregnancy loss on the survival of new-borns in the later age. Further, in addition to the large proportion of mortality that occurs within the first day (24 hours), the study has discovered that institutional deliveries had a modest moderation effect in the association of LBW with new-born mortality.

These results also demonstrate the need for life-course approach in the design of interventions. These interventions should include control of LBW and management or treatment of LBW new-borns. The LBW control interventions should target the socially and economically disadvantaged women who include the adolescents, unmarried women and rural residents with information on birth preparedness and care for pregnancies. For the LBW new-borns including multiple birth, I proposal a postnatal treatment and care package for mothers and health workers throughout the life-course of the new-born. Intensifying the health systems

For peer review only - http://bmjopen.bmj.com/site/about/guidelines.xhtml

through replication of known LBW effective strategies such identification of LBW cases through immediate new-born weighing and their management through for instance kangaroo mother care, better nutrition and other medical interventions is needed.

Additionally, the effect of institutional delivery in such resources limited setting is complex to understand because of plural health services' points that women go through before reaching the formal care points. The challenges of inadequate amenities for maternal and new-born health including qualified staff may make it difficult for the health facilities to appropriately address obstetric emergencies. I suggest a comprehensive health systems' audit both internal and external to provide an insight into the reasons the insignificant effect of institutional deliveries on the survival of LBW new-borns and the large proportion of death within the first day of life.

Acknowledgement

Drs Tiziana Leone and Arjan Gjonca advised on the design of the study and frequently reviewed the statistical approaches in addition to reviewing the manuscript. Miss Tryphena Nareeba, Dr Dan Kajungu and Dr Peter Waiswa supported me to access the Iganga-Mayuge HDSS data. Dr Peter Waiswa also gave me valuable comments that improved this manuscript. I am also grateful for the HDSS data collection team and the mothers who tirelessly provide such information.

Funding

This study is part of my PhD project that is being funded by the London School of Economics and Political Science. The funder had no role in study design, data collection and analysis, decision to publish, or preparation of the manuscript.

Reference

1 Dickson KE, Simen-Kapeu A, Kinney M V., *et al.* Every Newborn: Health-systems

For peer review only - http://bmjopen.bmj.com/site/about/guidelines.xhtml

bottlenecks and strategies to accelerate scale-up in countries. *Lancet* 2014;**384**:438–54. doi:10.1016/S0140-6736(14)60582-1

2 Darmstadt, L. G, Bhutta ZA, Cousens S, *et al.* Evidence-based, cost-effective interventions: How many newborn babies can we save? *Lancet* 2005;**365**:977–88. doi:10.1016/S0140-6736(05)71088-6

3 Zupan J. Perinatal mortality in developing countries. *N Engl J Med* 2005;**352**:2047–8. doi:10.1056/NEJMp058032

4 United Nations Inter-agency Group for Child Mortality Estimation (UN IGME). Levels & Trends in Child Mortality: Report 2018, Estimates developed by the United Nations Inter-agency Group for Child Mortality Estimation'. New York: 2018.

5 United Nations Inter-agency Group for Child Mortality Estimation (UN IGME). Levels & Trends in Child Mortality: Report 2017, Estimates Developed by the UN Inter-agency Group for Child Mortality Estimation. New York: 2017. http://www.childmortality.org/files_v21/download/IGME report 2017 child mortality final.pdf

6 United Nations Inter-agency Group for Child Mortality Estimation (UN IGME). Levels & Trends in Child Mortality: Report 2019, Estimates Developed by the UN Inter-agency Group for Child Mortality Estimation. 2019.

7 Sankar MJ, Natarajan CK, Das RR, *et al.* When do newborns die? A systematic review of timing of overall and cause-specific neonatal deaths in developing countries. *J Perinatol* 2016;**36**:S1–11. doi:10.1038/jp.2016.27

8 Reinebrant HE, Leisher SH, Coory M, *et al.* Making stillbirths visible: a systematic review of globally reported causes of stillbirth. *BJOG An Int J Obstet Gynaecol* 2018;**125**:212–24. doi:10.1111/1471-0528.14971

9 Blencowe H, Cousens S, Oestergaard MZ, *et al.* National , regional , and worldwide estimates of preterm birth rates in the year 2010 with time trends since 1990 for selected countries : a systematic analysis and implications. *Lancet* 2012;**379**:2162–72. doi:10.1016/S0140-6736(12)60820-4

10 Lawn JE, Blencowe H, Oza S, *et al.* Every Newborn 2 Every Newborn : progress , priorities , and potential beyond. Published Online First: 2015. doi:10.1016/S0140-6736(14)60496-7

11 Barros FC, Matijasevich A, Domingues MR, *et al.* How many low birthweight babies in low- and middle- income countries are preterm ? *Rev Saúde Pública* 2011;**45**:607–16.dx.doi.org/10.1590/S0034-89102011005000019

12 Marsh DR, Darmstadt GL, Moore J, *et al.* Advancing newborn health and survival in developing countries: a conceptual framework. *J Perinatol* 2002;**22**:572–6. doi:10.1038/sj.jp.7210793

13 Mosley WH, Chen LC. An analytical framework for the study of child survival in developing countries. *Popul Dev Rev* 1984;**10**:25–45. doi:10.2307/2807954

14 Sharma V, Katz J, Mullany LC, *et al.* Young Maternal Age and the Risk of Neonatal Mortality in Rural Nepal. 2009;**162**:828–35. doi:10.1001/archpedi.162.9.828.Young

15 Sinha S, Sc M, Indian F. Association between maternal age at childbirth and perinatal and under-five mortality in a prospective birth cohort from Delhi. *Indian Pediatr* 2017;**53**:871–7.

16 Kibria GM Al, Burrowes V, Choudhury A, *et al.* Determinants of early neonatal mortality in Afghanistan: An analysis of the Demographic and Health Survey 2015. *Global Health* Published Online First: 2018. doi:10.1186/s12992-018-0363-8

17 Fawole AO, Shah A, Tongo O, *et al.* Determinants of perinatal mortality in Nigeria. *Int J Gynecol Obstet* Published Online First: 2011. doi:10.1016/j.ijgo.2011.01.013

18 Grønvik T, Sandøy IF. Complications associated with adolescent childbearing in Sub-

For peer review only - http://bmjopen.bmj.com/site/about/guidelines.xhtml

Saharan Africa: A systematic literature review and meta-analysis. *PLoS One* 2018;**13**:1–21. doi:10.1371/journal.pone.0204327
19 Manyeh AK, Kukula V, Odonkor G, *et al.* Socioeconomic and demographic determinants of birth weight in southern rural Ghana : evidence from Dodowa Health and Demographic Surveillance System. *BMC Pregnancy Childbirth* 2016;:1–9. doi:10.1186/s12884-016-0956-2
20 Blencowe H, Krasevec J, de Onis M, *et al.* National, regional, and worldwide estimates of low birthweight in 2015, with trends from 2000: a systematic analysis. *Lancet Glob Heal* 2019;**7**:e849–60. doi:10.1016/S2214-109X(18)30565-5
21 Lean SC, Derricott H, Jones RL, *et al.* Advanced maternal age and adverse pregnancy outcomes: A systematic review and meta-analysis. *PLoS One* Published Online First: 2017. doi:10.1371/journal.pone.0186287
22 Althabe F, Moore JL, Gibbons L, *et al.* Adverse maternal and perinatal outcomes in adolescent pregnancies : The Global Network ' s Maternal Newborn Health Registry study. *Reprod Health* 2015;**12**:S8. doi:10.1186/1742-4755-12-S2-S8
23 Weng YH, Yang CY, Chiu YW. Risk assessment of adverse birth outcomes in relation to maternal age. *PLoS One* Published Online First: 2014. doi:10.1371/journal.pone.0114843
24 Blomberg M, Tyrberg RB, Kjølhede P. Impact of maternal age on obstetric and neonatal outcome with emphasis on primiparous adolescents and older women : a Swedish Medical Birth Register Study. *BMJ Open* 2014;**4**:e005840. doi:10.1136/bmjopen-2014-005840
25 McKinnon B, Harper S, Kaufman JS, *et al.* Socioeconomic inequality in neonatal mortality in countries of low and middle income: A multicountry analysis. *Lancet Glob Heal* Published Online First: 2014. doi:10.1016/S2214-109X(14)70008-7
26 Lohela TJ, Nesbitt RC, Pekkanen J, *et al.* Comparing socioeconomic inequalities between early neonatal mortality and facility delivery: Cross-sectional data from 72 low- and middle-income countries. *Sci Rep* Published Online First: 2019. doi:10.1038/s41598-019-45148-5
27 Houweling TAJ, Kunst AE. Socio-economic inequalities in childhood mortality in low- and middle-income countries : a review of the international evidence. 2017;:7–26. doi:10.1093/bmb/ldp048
28 Ronsmans C, Holtz S, Stanton C. Socioeconomic differentials in caesarean rates in developing countries: a retrospective analysis. *Lancet* Published Online First: 2006. doi:10.1016/S0140-6736(06)69639-6
29 Fenn B, Kirkwood BR, Popatia Z, *et al.* Inequities in neonatal survival interventions: Evidence from national surveys. *Arch Dis Child Fetal Neonatal Ed* Published Online First: 2007. doi:10.1136/adc.2006.104836
30 Barros AJ, Ronsmans C, Axelson H, *et al.* Equity in maternal, newborn, and child health interventions in Countdown to 2015: A retrospective review of survey data from 54 countries. *Lancet* Published Online First: 2012. doi:10.1016/S0140-6736(12)60113-5
31 Austin P, Ntenda M, Chuang K, *et al.* Factors Associated with Infant Mortality in Malawi. *J Exp Clin Med* 2014;**6**:125–31. doi:10.1016/j.jecm.2014.06.005
32 Kayode GA, Adekanmbi VT, Uthman OA. Risk factors and a predictive model for under-five mortality in Nigeria : evidence from Nigeria demographic and health survey. *BMC Pregnancy Childbirth* 2012;**12**:10. doi:10.1186/1471-2393-12-10
33 Accrombessi M, Zeitlin J, Massougbodji A, *et al.* What Do We Know about Risk Factors for Fetal Growth Restriction in Africa at the Time of Sustainable Development Goals? A Scoping Review. Paediatr. Perinat. Epidemiol. 2018. doi:10.1111/ppe.12433

For peer review only - http://bmjopen.bmj.com/site/about/guidelines.xhtml

34 Pereira PP da S, Da Mata FAF, Figueiredo ACG, *et al.* Maternal active smoking during pregnancy and low birth weight in the americas: A systematic review and meta-analysis. Nicotine Tob. Res. 2017. doi:10.1093/ntr/ntw228
35 World Health Organization (WHO). *WHO Recommendation on Antenatal care for positive pregnancy experience*. Geneva, Switzerland: : World Health Organization 2016. doi:ISBN 978 92 4 154991 2
36 Darmstadt GL, Marchant T, Claeson M, *et al.* A strategy for reducing maternal and newborn deaths by 2015 and beyond. *BMC Pregnancy Childbirth* 2013;**13**:216. doi:10.1186/1471-2393-13-216
37 Wall SN, Lee ACC, Carlo W, *et al.* Reducing Intrapartum-Related Neonatal Deaths in Low- and Middle-Income Countries-What Works? *Semin Perinatol* 2010;**34**:395–407. doi:10.1053/j.semperi.2010.09.009
38 Salam RA, Mansoor T, Mallick D, *et al.* Essential childbirth and postnatal interventions for improved maternal and neonatal health. *Reprod Health* 2014;**11**:1–17. doi:10.1186/1742-4755-11-S1-S3
39 Campbell S. Fetal macrosomia: A problem in need of a policy. *Ultrasound Obstet Gynecol* 2014;**43**:3–10. doi:10.1002/uog.13268
40 Darmstadt GL, Bhutta Z a., Cousens S, *et al.* Evidence-based, cost-effective interventions: How many newborn babies can we save? *Lancet* 2005;**365**:977–88. doi:10.1016/S0140-6736(05)71088-6
41 Berkley S, Dybul M, Godal T, *et al.* Integration and innovation to advance newborn survival. *Lancet* 2014;**384**:e22–3. doi:10.1016/S0140-6736(14)60691-7
42 Benova L, Tunçalp Ö, Moran AC, *et al.* Not just a number: examining coverage and content of antenatal care in low-income and middle-income countries. *BMJ Glob Heal* 2018;**3**:e000779. doi:10.1136/bmjgh-2018-000779
43 Goldenberg RL, McClure EM, Saleem S. Improving pregnancy outcomes in low- and middle-income countries. *Reprod Health* 2018;**15**:2017–8. doi:10.1186/s12978-018-0524-5
44 Pathirana J, Muñoz FM, Abbing-Karahagopian V, *et al.* Neonatal death: Case definition & guidelines for data collection, analysis, and presentation of immunization safety data. *Vaccine* 2016;**34**:6027–37. doi:10.1016/j.vaccine.2016.03.040
45 Da Silva FT, Gonik B, McMillan M, *et al.* Stillbirth: Case definition and guidelines for data collection, analysis, and presentation of maternal immunization safety data. *Vaccine* 2016;**34**:6057–68. doi:10.1016/j.vaccine.2016.03.044
46 Cutland CL, Lackritz EM, Mallett-Moore T, *et al.* Low birth weight: Case definition & guidelines for data collection, analysis, and presentation of maternal immunization safety data. *Vaccine* 2017;**35**:6492–500. doi:10.1016/j.vaccine.2017.01.049
47 Hair JF, Black WC, Babin BJ, *et al. Multivariate Data Analysis*. Seventh Ed. London: : Pearson Education Limited 2006.
48 Graham JW, Olchowski AE, Gilreath TD. How many imputations are really needed? Some practical clarifications of multiple imputation theory. *Prev Sci* 2007;**8**:206–13. doi:10.1007/s11121-007-0070-9
49 Madley-Dowd P, Hughes R, Tilling K, *et al.* The proportion of missing data should not be used to guide decisions on multiple imputation. *J Clin Epidemiol* 2019;**110**:63–73. doi:10.1016/j.jclinepi.2019.02.016
50 Lee J, Huber JJ. Multiple imputation with large proportions of missing data: How much is too much? United Kingdom Stata Users' Gr. Meet. 2011. 2011.
51 Coveney J. 'FIRTHLOGIT: Stata module to calculate bias reduction in logistic regression,' Statistical Software Components S456948, Boston College Department of Economics, revised 25 Jul 2015. 2008.

For peer review only - http://bmjopen.bmj.com/site/about/guidelines.xhtml

52 Das JK, Kumar R, Salam RA, *et al.* Evidence from facility level inputs to improve quality of care for maternal and newborn health: interventions and findings. *Reprod Health* 2014;**11 Suppl 2**:S2. doi:10.1186/1742-4755-11-S2-S2

53 Lincetto O, Mothebesoane-anoh S, Gomez P, *et al.* Antenatal Care: Opprotunities for Africa's Newborns. *Int J Sci Technol Res* 2013;**2**:51–62.

54 Uganda Ministry of Health. National Health Facility Master List 2018. Kampala: 2018. http://library.health.go.ug/publications/health-facility-inventory/national-health-facility-master-list-2017

55 Singh U, Ueranantasun A, Kuning M. Factors associated with low birth weight in Nepal using multiple imputation. 2017;:1–10. doi:10.1186/s12884-017-1252-5

56 Cohen JJ, Blevins M, Mapenzi A, *et al.* Overcoming the perceived barriers to health care access among single mothers in coastal Kenya. *Int J Public Health* Published Online First: 2014. doi:10.1007/s00038-013-0511-0

57 Kaiser JL, Fong RM, Hamer DH, *et al.* How a woman's interpersonal relationships can delay care-seeking and access during the maternity period in rural Zambia: An intersection of the Social Ecological Model with the Three Delays Framework. *Soc Sci Med* 2018;**220**:312–21. doi:10.1016/J.SOCSCIMED.2018.11.011

58 Peneza AK, Maluka SO. 'Unless you come with your partner you will be sent back home': strategies used to promote male involvement in antenatal care in Southern Tanzania. *Glob Health Action* 2018;**11**. doi:10.1080/16549716.2018.1449724

59 Påfs J, Musafili A, Binder-Finnema P, *et al.* 'They would never receive you without a husband': Paradoxical barriers to antenatal care scale-up in Rwanda. *Midwifery* 2015;**31**:1149–56. doi:10.1016/j.midw.2015.09.010

60 Sochas L. Women who break the rules: Social exclusion and inequities in pregnancy and childbirth experiences in Zambia. *Soc Sci Med* 2019;**232**:278–88. doi:10.1016/j.socscimed.2019.05.013

61 Al Kibria GM, Khanam R, Mitra DK, *et al.* Rates and determinants of neonatal mortality in two rural sub-districts of Sylhet, Bangladesh. *PLoS One* 2018;**13**:e0206795. doi:10.1371/journal.pone.0206795

62 Christiansen CS, Gibbs S, Chandra-Mouli V. Preventing early pregnancy and pregnancy-related mortality and morbidity in adolescents in developing countries: The place of interventions in the prepregnancy period. *J Pregnancy* 2013;**2013**:15–20. doi:10.1155/2013/257546

63 Uthman OA, Uthman MB, Yahaya I. A population-based study of effect of multiple birth on infant mortality in Nigeria. *BMC Pregnancy Childbirth* 2008;**8**:1–6. doi:10.1186/1471-2393-8-41

64 Monden CWS, Smits J. Mortality among twins and singletons in sub-Saharan Africa between 1995 and 2014: a pooled analysis of data from 90 Demographic and Health Surveys in 30 countries. *Lancet Glob Heal* 2017;**5**:e673–9. doi:10.1016/S2214-109X(17)30197-3

65 Dudenhausen JW, Maier RF. Perinatale probleme von mehrlingen. *Dtsch Arztebl* 2010;**107**:663–8. doi:10.3238/arztebl.2010.0663

66 Blondel B, Kogan MD, Alexander GR, *et al.* The impact of the increasing number of multiple births on the rates of preterm birth and low birthweight: An international study. *Am J Public Health* 2002;**92**:1323–30. doi:10.2105/AJPH.92.8.1323

67 Heather M W, Selina K W, Therese D, *et al.* Breastfeeding education and support for women with twins or higher order multiples. *Cochrane Database Syst Rev* Published Online First: 2017. doi:10.1002/14651858.CD012003.pub2.www.cochranelibrary.com

68 Montgomery KS, Cubera S, Belcher C, *et al.* Childbirth Education for Multiple Pregnancy. *J Perinat Educ* 2005;**14**:33–8. doi:10.1624/105812405x57561

For peer review only - http://bmjopen.bmj.com/site/about/guidelines.xhtml

69 Nattey C, Masanja H, Klipstein-Grobusch K. Relationship between household socio-economic status and under-five mortality in Rufiji DSS, Tanzania. *Glob Health Action* 2013;**6**:19278. doi:10.3402/gha.v6i0.19278

70 Patterson JA, Ford JB, Morris JM, *et al.* Trends and recurrence of stillbirths in NSW. *Aust N Z J Public Health* 2014;**38**:384–9. doi:10.1111/1753-6405.12179

71 Heazell AEP, Clewlow J. Protecting families from recurrent stillbirth. *BMJ* 2015;**350**:1–2. doi:10.1136/bmj.h3262

72 Getahun D, Lawrence JM, Fassett MJ, *et al.* The association between stillbirth in the first pregnancy and subsequent adverse perinatal outcomes. *Am J Obstet Gynecol* 2009;**201**:378.e1-378.e6. doi:10.1016/j.ajog.2009.06.071

73 Bocquier P, Sankoh O, Byass P. Are health and demographic surveillance system estimates sufficiently generalisable? *Glob Health Action* 2017;**10**. doi:10.1080/16549716.2017.1356621

Figure legends

Figure 1: Perinatal and New-born mortality distribution by days of death

Figure 2: Annual changes in perinatal mortality rates, neonatal mortality rates and LBW

Figure 3: New-born mortality and LBW stratified by maternal socio-economic characteristics, place of residence and place of delivery

Figure 4: New-born mortality and LBW stratified by individual characteristics

For peer review only

For peer review only - http://bmjopen.bmj.com/site/about/guidelines.xhtml

Figure 1: Perinatal and New-born mortality distribution by days of death

I. Distribution of perinatal mortality by days of death

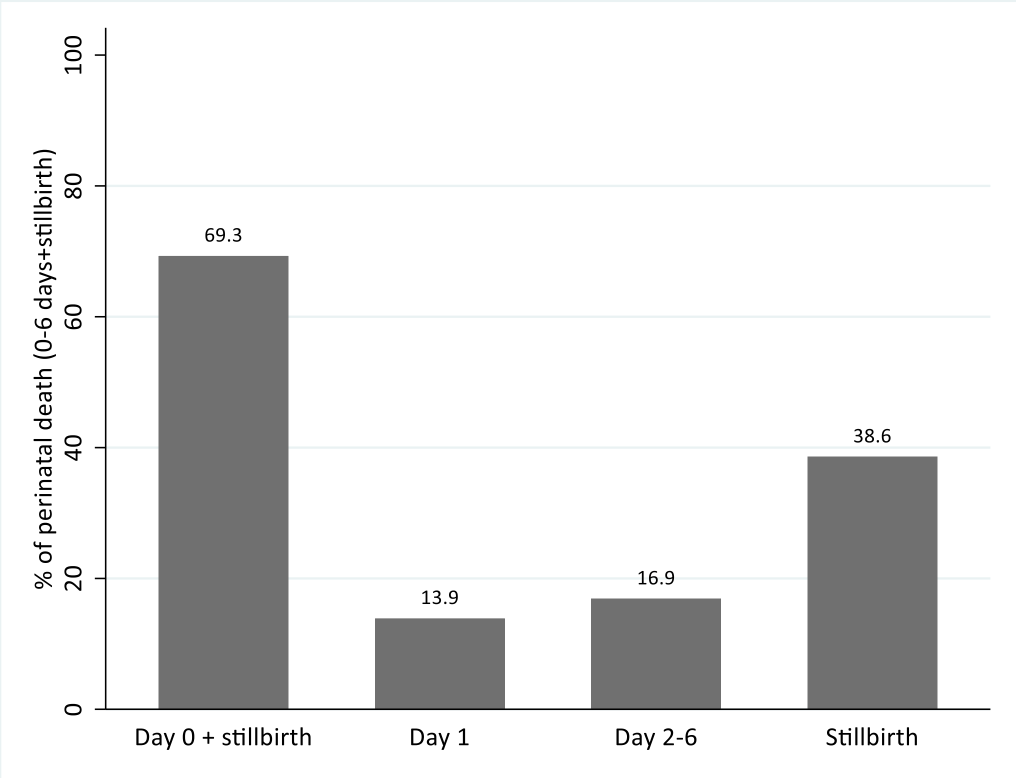

II. Distribution of neonatal mortality by days of death

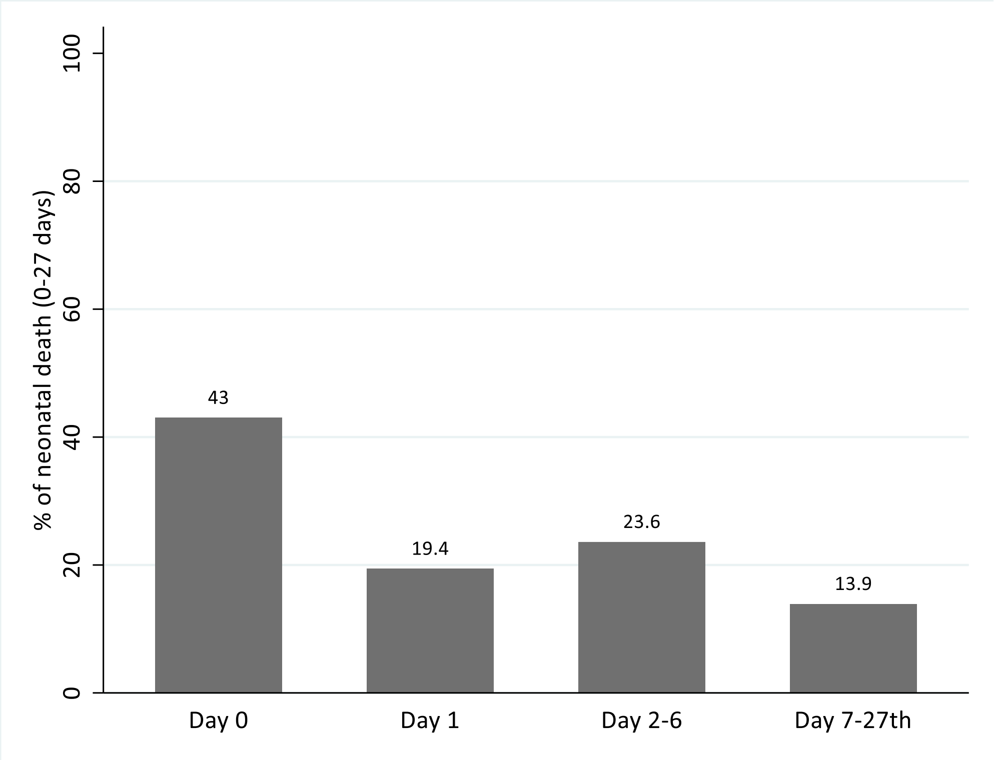

Figure 2: Annual changes in perinatal mortality rates, neonatal mortality rates and LBW

I. Perinatal mortality and Neonatal mortality annual estimates

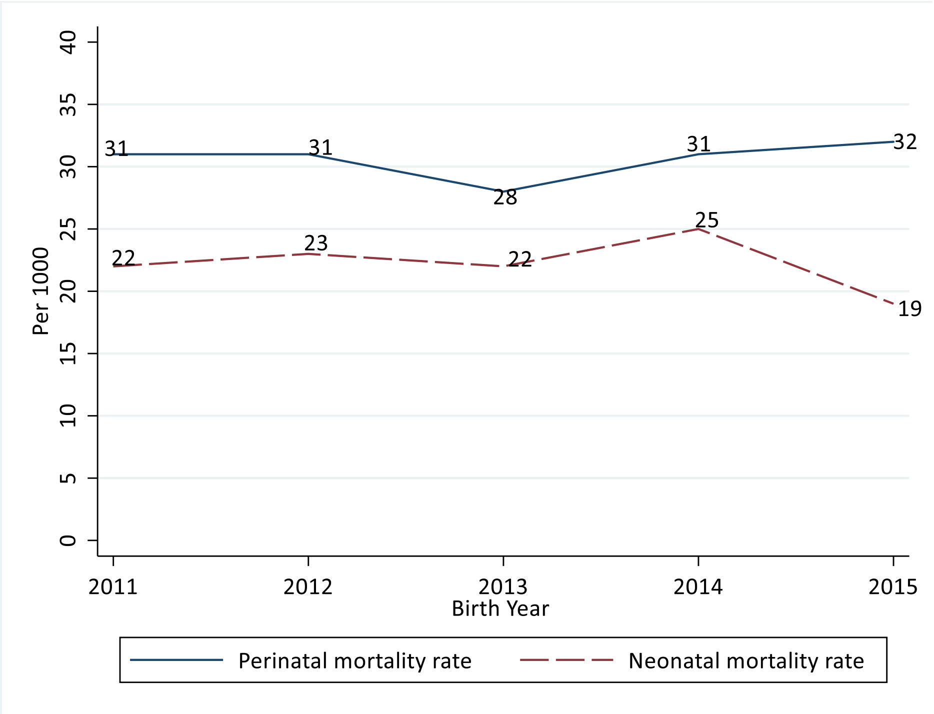

Note:

1. *Perinatal mortality rates are calculated as the number of births that resulted in stillbirths or died within 6 days after birth per total number of births (0-6 days + stillbirths).*
2. *Neonatal mortality is calculated as the number of newborns that died within 27 days immediately or during birth (0-27days)*

II. LBW annual estimates

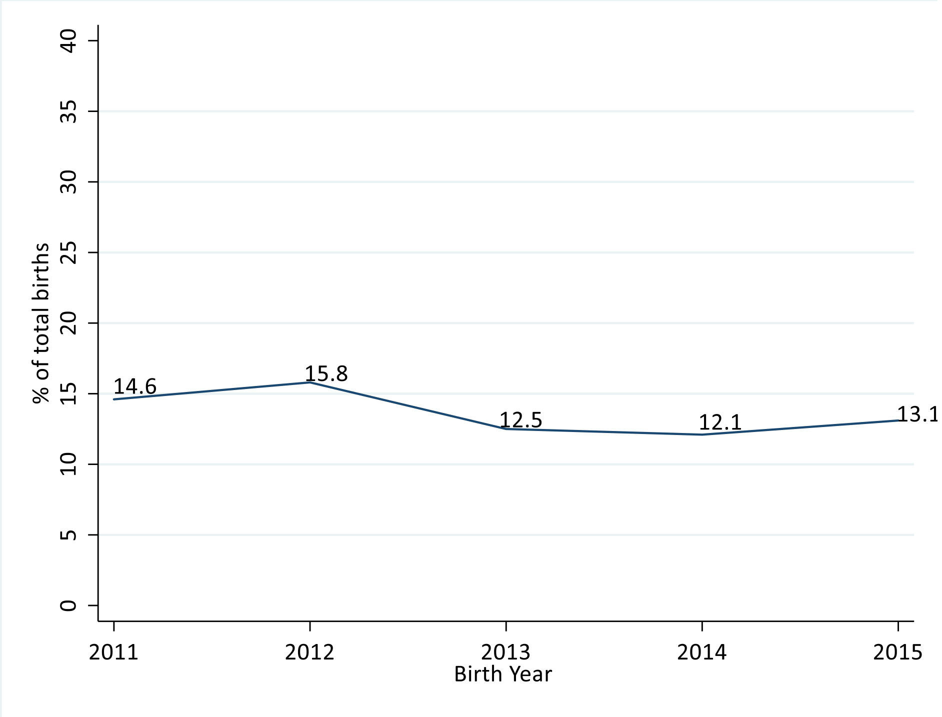

Figure 3: New-born mortality and LBW stratified by maternal socio-economic characteristics, place of residence and place of delivery

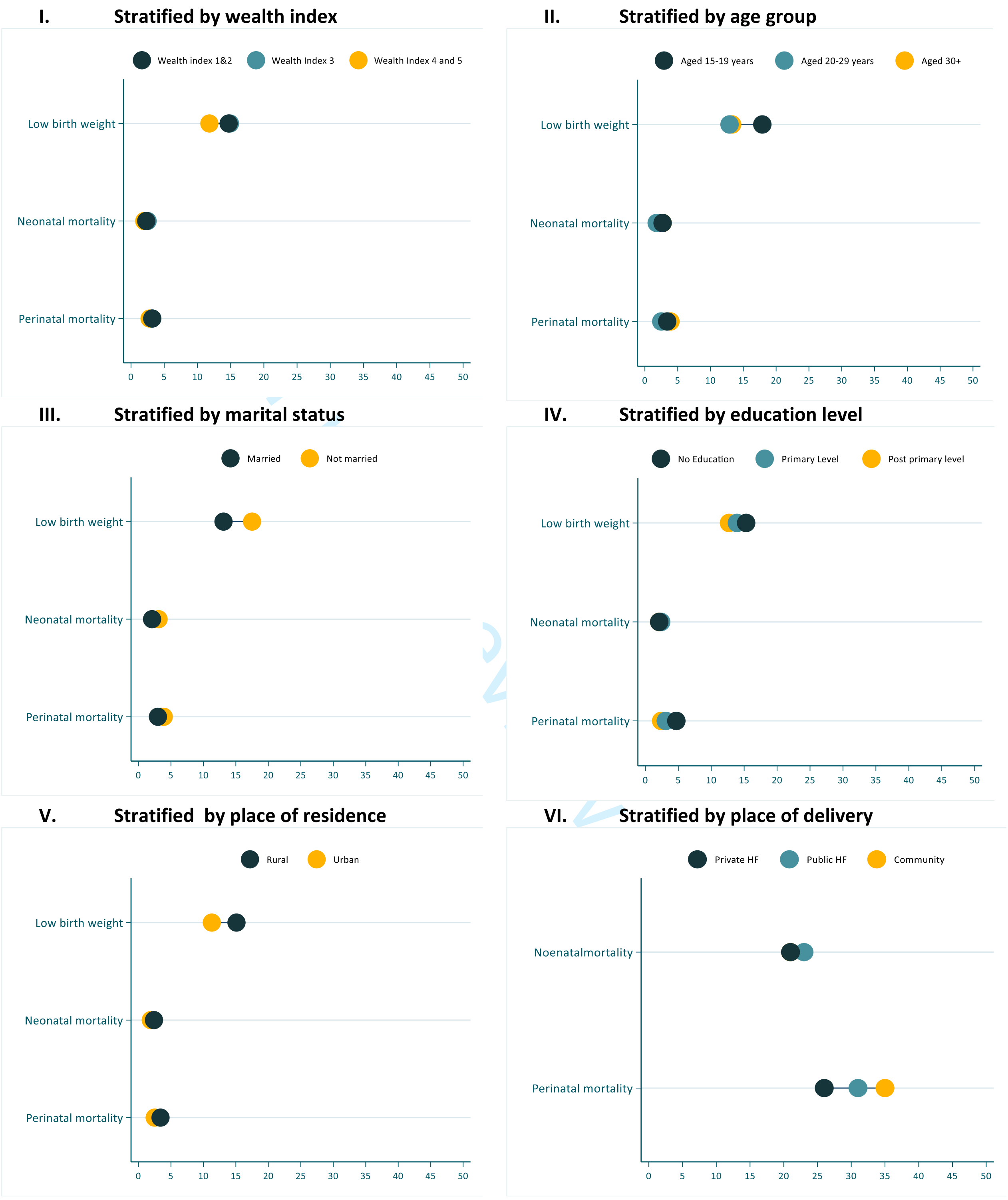

Figure 4: New-born mortality and LBW stratified by individual characteristics

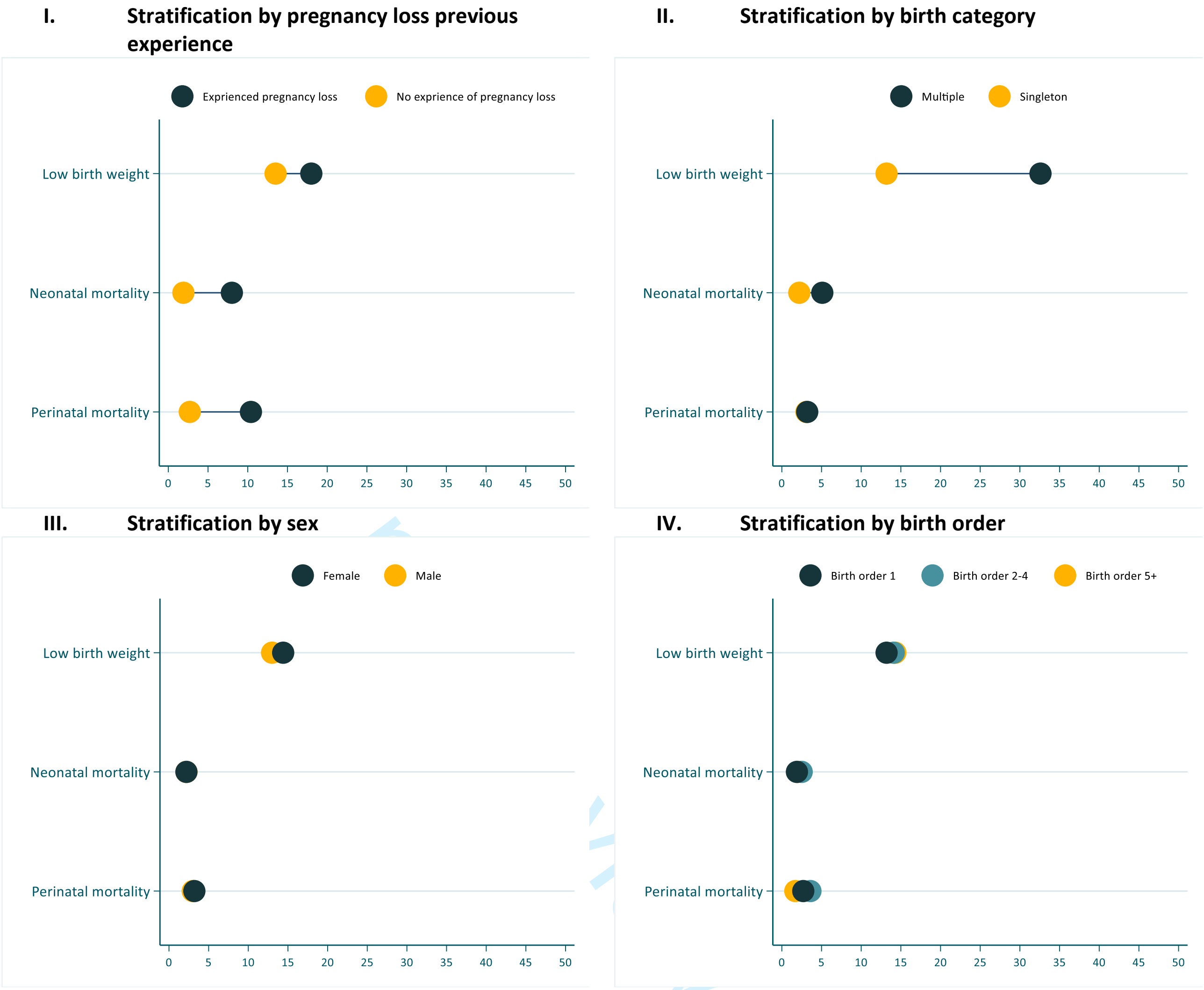

For peer review only - http://bmjopen.bmj.com/site/about/guidelines.xhtml

Supplement 1

Supplement 1 Table 1: Multicollinearity assessment for perinatal and neonatal mortality variables in the event history dataset

Variable	VIF	SQRT VIF	Tolerance	R-Squared
Birth season in quarters				
2	1.53	1.23	0.6557	0.3443
3	1.54	1.24	0.6481	0.3519
4	1.56	1.25	0.6404	0.3596
Child as Low birth weight	1.43	1.19	0.7006	0.2994
Child low birth weight and delivered in private facility	1.38	1.18	0.7238	0.2762
Child low birth weight and delivered in community	1.4	1.19	0.7118	0.2882
Primary Education	5.04	2.25	0.1982	0.8018
Post Primary Education	5.41	2.33	0.1848	0.8152
Delivered in private health facilities	1.3	1.14	0.7712	0.2288
Delivered in community	1.25	1.12	0.7991	0.2009
Maternal age				
<20 years	1.3	1.14	0.7707	0.2293
30+ years	1.19	1.09	0.8435	0.1565
Birth order				
1	1.39	1.18	0.7208	0.2792
5+	1.06	1.03	0.9397	0.0603
Rural residence	2.23	1.49	0.4477	0.5523
Has a partner	1.19	1.09	0.8412	0.1588
Experienced pregnancy or child loss previously	1.04	1.02	0.9631	0.0369
Multiple birth	1.02	1.01	0.9833	0.0167
Child sex as female	1	1	0.997	0.003
Wealth index				
Index 3	1.26	1.12	0.794	0.206
Index 4 and 5	2.81	1.68	0.3557	0.6443
Mean VIF	1.78			

For peer review only - http://bmjopen.bmj.com/site/about/guidelines.xhtml

Supplement 1 Table 2: Assessment of missing variable factors for birth weight and wealth index in the event history dataset

	(1) Birth weight missingness	std.Err	(2) Wealth index missingness	std.Err
**Time of death**				
Day 1 and above	1	-	1	-
Still birth and day 0	9.137***	(12.47)	-	-
**Place of birth**			-	-
Hospital	1	-	-	-
Community	3.343***	(20.14)	-	-
Private	2.680***	(16.17)	-	-
**Place of residence**			-	-
Urban	1	-	-	-
Rural	1.779***	(10.28)	0.459***	(-13.56)
**Age group**				
<20 years	1.145	(1.94)	1.226*	(2.44)
20-29	1	-	1	-
30+	0.947	(-1.05)	0.619***	(-7.41)
**Education level**				
None	1	-	1	-
Primary	0.759***	(-4.21)	0.985	(-0.11)
Post primary	0.568***	(-7.60)	1.250	(1.68)
**Marital status**				
Has no a partner	-	-	1	-
Has a partner	-	-	2.010***	(7.38)

Exponentiated coefficients; t statistics in parentheses * p<0.05, ** p<0.01, *** p<0.001

For peer review only

Supplement 1 Figure 1: Relationship between age and birth outcomes using for age categorization using event histories data

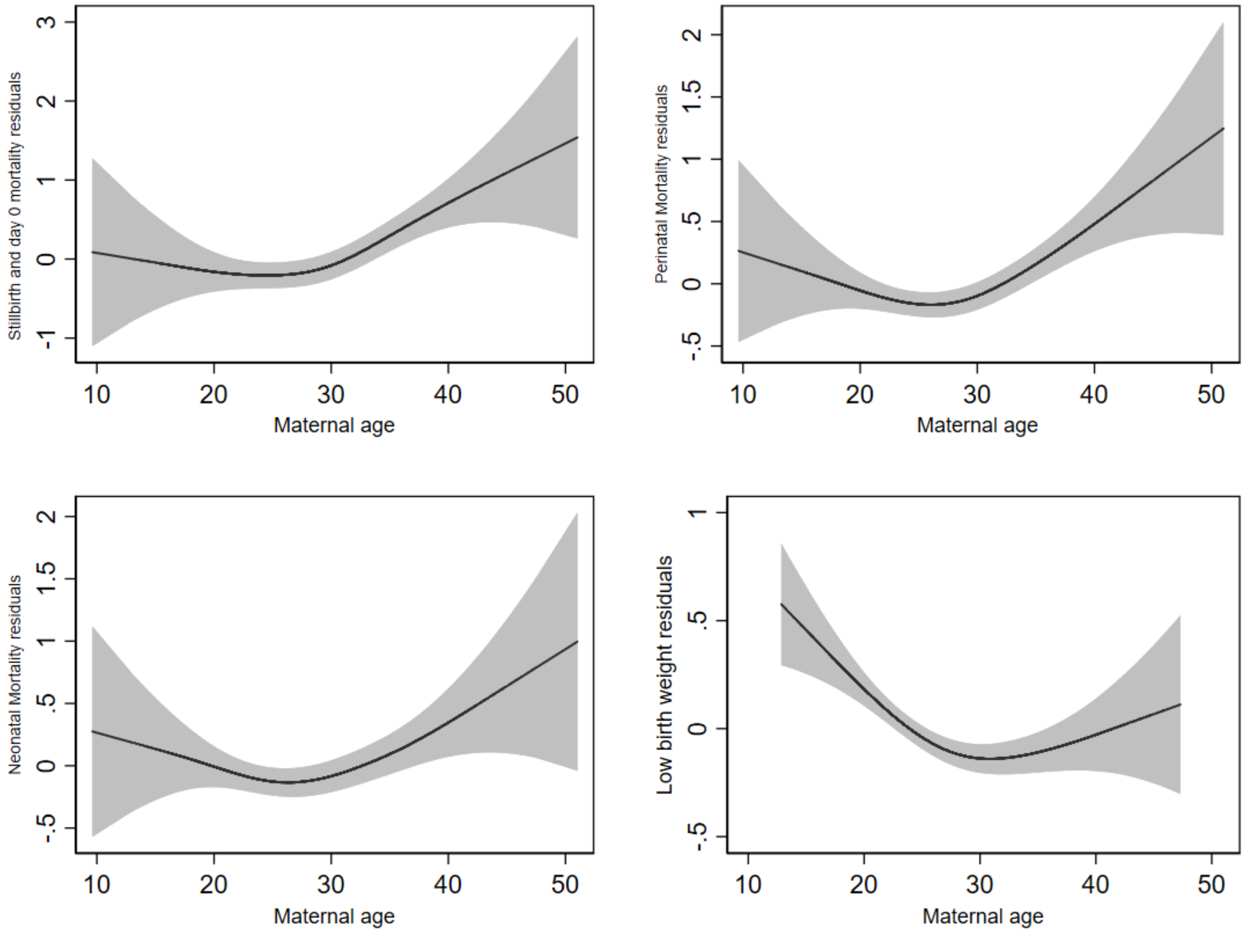

only

Supplement 1 Figure 2: Comparing the LBW and household poor status for imputed and completed cases

I. LBW estimates for imputed and completed cases analysis

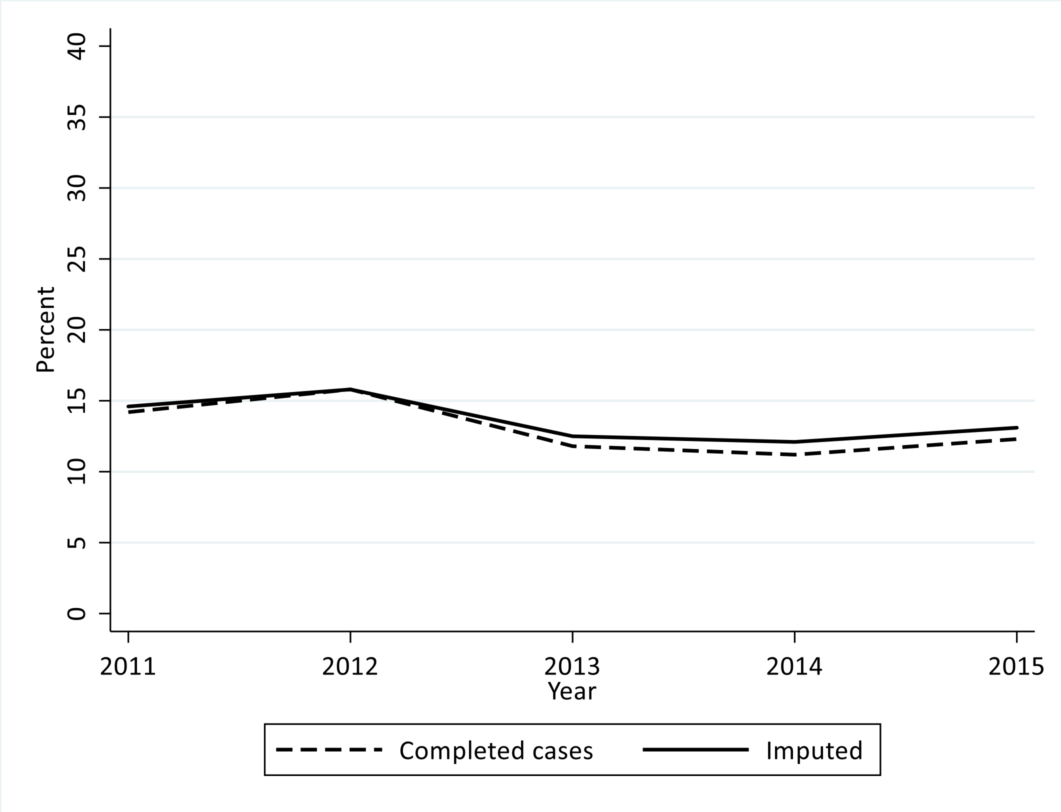

II. Household poor status (wealth index 1 and 2) estimates for imputed and completed cases analysis

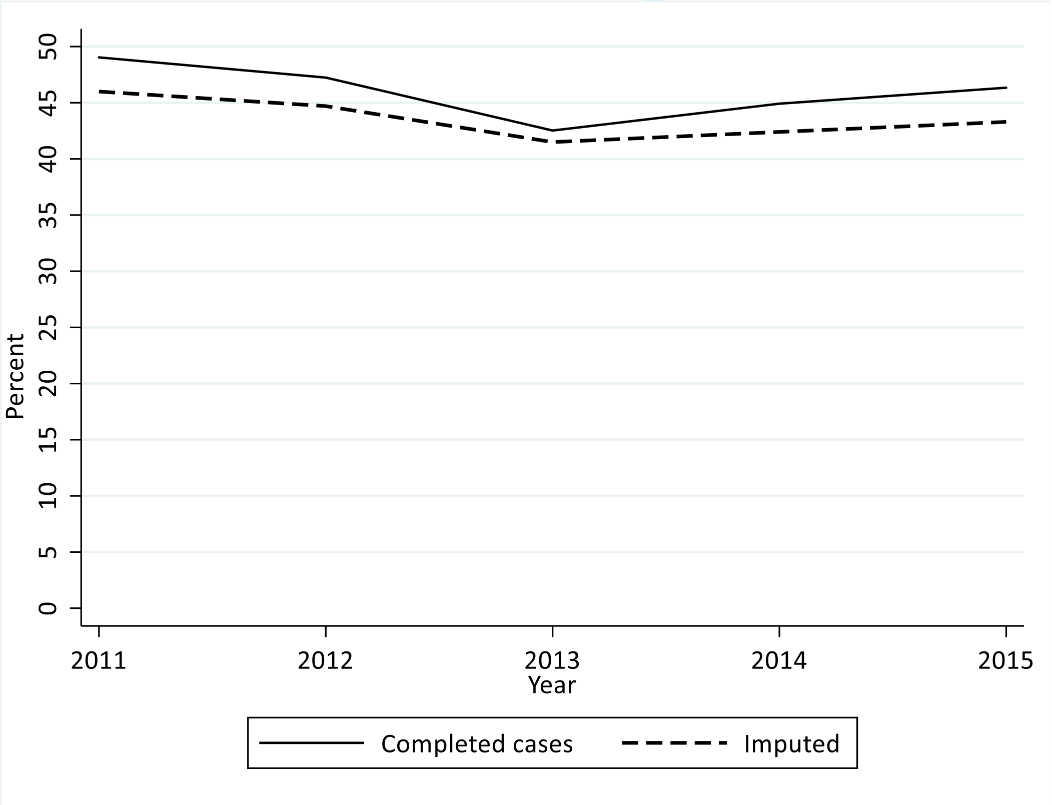

For peer review only - http://bmjopen.bmj.com/site/about/guidelines.xhtml

Supplement 1 Table 3: Bivariate analysis comparing logistic and firth logit model using 2011-2015 Iganga-Mayuge event history data

	Perinatal mortality				Late neonatal mortality			
	Logit		Firth logit		Logit		Firth logit	
	OR	P-value	OR	P-value	OR	P-value	OR	P-value
**Annual birth quarters**								
1	1.00	-	1.00	-	1.00	-	1.00	-
2	1.08	0.63	1.08	0.63	1.55	0.41	1.51	0.42
3	1.26	0.14	1.26	0.14	1.63	0.35	1.58	0.36
4	1.20	0.27	1.20	0.27	1.43	0.51	1.40	0.52
**Childbirth weight**								
<2.5 Kgs	2.52	<0.001	2.53	<0.001	2.37	0.05	2.45	0.04
25 Kgs+								
**Maternal education level**								
None	1.69	<0.001	1.70	<0.001	1.37	0.49	1.46	0.39
Primary+	1.00	-	1.00	-	1.00	-	1.00	-
**Place of delivery**								
Health facility	1.01	0.91	1.01	0.93	1.22	0.61	1.18	0.67
Community	1.00	-	1.00	-	1.00	-	1.00	-
**Maternal age (years)**								
<20	1.12	0.46	1.13	0.42	0.88	0.80	0.97	0.95
20—29	1.00	-	1.00	-	1.00	-	1.00	-
30+	1.48	<0.001	1.48	<0.001	1.73	0.12	1.74	0.11
**Childbirth order**								
1	0.80	0.07	0.80	0.07	0.70	0.32	0.71	0.33
2—4	1.00	-	1.00	-	1.00	-	1.00	-
5th+	0.46	0.06	0.49	0.07	2.20	0.20	2.52	0.10
**Place of residence**								
Urban	1.00	-	1.00	-	1.00	-	1.00	-
Rural	1.40	0.01	1.39	0.01	0.77	0.45	0.76	0.43
**Marital status**								
No partner	1.00	-	1.00	-	1.00	-	1.00	-
Has a partner	0.74	0.07	0.74	0.07	0.63	0.31	0.60	0.24
**Experienced neonatal or pregnancy loss previously**								
No								
Yes	4.18	0.00	4.20	<0.001	3.60	0.01	3.89	0.00
**Birth category**								
Singleton	1.00	-	1.00	-	1.00	-	1.00	-
Multiple	1.02	0.96	1.08	0.83	7.70	<0.001	8.31	0.00
**Child sex**								
Male	1.00	-	1.00	-	1.00	-	1.00	-
Female	1.06	0.68	1.06	0.68	0.57	0.12	0.58	0.12
**Household Wealth**								

For peer review only

For peer review only - http://bmjopen.bmj.com/site/about/guidelines.xhtml

	Perinatal mortality				Late neonatal mortality			
	Logit		Firth logit		Logit		Firth logit	
	OR	P-value	OR	P-value	OR	P-value	OR	P-value
Poor(er) (1-2)	1.10	0.43	1.10	0.43	0.73	0.39	0.75	0.41
Less poor (3-5)	1.00	-	1.00	-	1.00	-	1.00	-

Supplement 1 Table 4: Indirect pathways of the association between LBW and perinatal mortality.

Variables	Indirect effect	Se	P-value
Adolescence (<20 years)	0.03	0.12	0.84
Rural residence	0.11	0.06	0.05
Having a partner	0.08	0.06	0.14
Experienced neonatal or pregnancy loss previously	0.40	0.20	0.04
Multiple birth	1.14	0.15	<0.001

For peer review only

For peer review only - http://bmjopen.bmj.com/site/about/guidelines.xhtml

Supplement 2: Analyisis of Mediation effect of LBW on newborn mortality

Let y_i represents the outcome of interest as a binary variable (i) that takes the probability of the presence of outcome's exposure as P and probability of absence of the outcome's exposure as 1-P. The bivariate regression model would be of the form as in Eq. 1

$$ln\left(\frac{P_j}{1-P_j}\right) = \alpha_i + \beta_i x_i \qquad (1)$$

Where *j* represents categories of perinatal, late neonatal mortality, and LBW, x_i covariates of choice for each of the outcome and β_i their respective coefficients.

To assess the moderation effect of health facility delivery on the effect LBW on perinatal mortality (*pm*), an interaction term between the LBW and institutional deliver (*hf*) was introduced as indicated in Eq. 2.

$$ln\left(\frac{P_{pm}}{1-P_{pm}}\right) = \alpha_i + \beta_{lbw}x_{lbw} + \beta_{hf}x_{hf} + \beta_{lbw*hf}(x_{lbw} * x_{hf}) \qquad (2)$$

Subsequently, a multivariable regression model controlling for other factors added on eq. 2 was run (Eq. 3). In this model, I also assessed how the intersection between birth order and maternal age would affect perinatal mortality as shown in Eq. 3.

$$ln\left(\frac{P_{pm}}{1-P_{pm}}\right) = \alpha_i + \beta_{lbw}x_{lbw} + \beta_{hf}x_{hf} + \beta_{lbw*hf}(x_{lbw} * x_{hf}) + \beta_{Age}x_{Age} + \beta_{educ}x_{educ} + \beta_{birth\ order}x_{birth\ order} + (\beta_{birth\ order*Age}(x_{birth\ order} * x_{Age}) + \beta_{multiple\ birth}x_{multiple\ birth} + \beta_{wealth}x_{wealth} + \beta_{nm\ expreinced\ preiviously}x_{nm\ expreinced\ preiviously} + \beta_{sex}x_{sex} + \beta_{birth\ quarter}x_{birth\ quarter} + \beta_{marital\ status}x_{marital\ \ status} \qquad (3)$$

The independent variables that were included under the multivariate regression model for the late neonatal mortality (*nm*) are as indicated in Eq. 4

$$ln\left(\frac{P_{nm}}{1-P_{nm}}\right) = \alpha_i + \beta_{lbw}x_{lbw} + \beta_{Age}x_{Age} + \beta_{educ}x_{educ} + \beta_{birth\ order}x_{birth\ order} + \beta_{multiple\ birth}x_{multiple\ birth} + \beta_{wealth}x_{wealth} + \beta_{nm\ expreinced\ preiviously}x_{nm\ expreinced\ preiviously} + \beta_{sex}x_{sex} + \beta_{birth\ quarter}x_{birth\ quarter} + \beta_{marital\ status}x_{marital\ \ status} \qquad (4)$$

For peer review only

The mediating variable was selected if the predetermined endogenous – in this case, LBW was strongly associated with the main outcome variable in the multivariate model. The effect of selected covariates on LBW was determined using a multivariate regression model as indicated in Eq. 5

$$ln\left(\frac{P_{lbw}}{1-P_{lbw}}\right) = \alpha_i + \beta_{age}x_{age} + \beta_{educ}x_{educ} + \beta_{birth\ order}x_{birth\ order} + \beta_{multiple\ birth}x_{multiple\ birth} + \beta_{wealth}x_{wealth} + \beta_{nm\ expreinced\ preiviously}x_{nm\ expreinced\ preiviously} + \beta_{sex}x_{sex} + \beta_{birth\ quarter}x_{birth\ quarter} + \beta_{marital\ status}x_{marital\ status} \quad (5)$$

The indirect effect for instance for perinatal mortality with their corresponding standard errors for the factors that were significant in the LBW model were calculated as indicated in Eq. 6.

$$indirect\ \ effect_i = \beta_{lbw\ i} * \beta_{pm\ i} \quad (6)$$

The indirect effect's standard errors were calculated based on Goodman's approach of the product of coefficients [1] as indicated in Eq. 7.

$$\sigma_{\beta lbw\ i*\beta pm\ i} = \sqrt{\sigma^2_{lbw\ i} * \beta^2_{pm\ i} + \sigma^2_{pm\ i} * \beta^2_{lbw\ i} - \sigma^2_{lbw\ i} * \sigma^2_{pm\ i}} \quad (7)$$

The corresponding z or t -value were calculated as in Eq. 8;

$$z_i = \frac{indirect\ \ effect_i}{\sigma_{\beta lbw\ i*\beta pm\ i}} \quad (8)$$

Eq. 8 was used to calculate the P-values (P_i) for each indirect variable's coefficient as recommend by [2,3] in Eq. 9;

$$P_i = e^{-0.717138*z_i - 0.415973*z_i^2} \quad (9)$$

Reference

1 MacKinnon DP, Lockwood CM, Hoffman JM, *et al.* A comparison of methods to test mediation and other intervening variable effects. *Psychol Methods* 2002;**7**:83–104. doi:10.1037/1082-989X.7.1.83
2 Altman DG, Bland JM. Statistics notes: How to obtain the P value from a confidence interval. *BMJ* 2011;**343**:1–2. doi:10.1136/bmj.d2304
3 Altman DG, Bland JM. How to obtain the confidence interval from a P value. *BMJ* 2011;**343**:1–2. doi:10.1136/bmj.d2090

For peer review only

Supplement 3 Table 1: 2011-2015 Iganga-Mayuge HDSS event histories participants' descriptive statistics

	Complete case*		Imputed
	n=10,758		
	%ge	*missing*	*%ge*
**Institutional delivery**		**1.0**	
Public Health facility	68.7		68.7
Community	16.0		16.0
Private health facility	15.4		15.4
**Maternal education level**		**0.5**	
None	14.3		14.3
Primary	52.3		52.3
Post primary	33.4		33.4
**Maternal age group (years)**		**0.0**	
<20	13.7		-
20-29	53.6		-
30+	32.8		-
**Place of residence**		**0.0**	
Rural	64.0		-
Urban	36.0		-
**Marital status**		**9.1**	
Has a partner or married	86.2		86.2
Has no a partner	13.8		13.8
**Household wealth index**		**18.8**	
Index 1 and 2	46.0		43.7
Index 3	21.2		20.6
Index 4 and 5	32.8		35.7
**Previous experience of death**		**0.0**	
Experienced pregnancy loss	5.1		-
Never experienced pregnancy loss	94.9		-
**Childbirth weight**		**27.3**	
LBW (<2.5Kgs)	13.1		13.7
Normal Birth weight	86.9		86.3
**Childbirth order**		**1.2**	
First	45.0		45.0
2nd-4th	50.6		50.7
5th +	4.3		4.3
**Child sex**		**1.2**	
Female	50.2		50.2
Male	49.8		49.8
**Birth category**		**0.9**	
Multiple	2.4		2.4
Singleton	97.6		97.6
**Birth season in annual quarters**		**0.0**	

For peer review only

	Complete case*		Imputed
	n=10,758		
	%ge	*missing*	*%ge*
1	25.2		-
2	24.6		-
3	26.1		-
4	24.1		-
**Birth Year**		**0.0**	
2011	21.9		-
2012	21.6		-
2013	20.5		-
2014	17.9		-
2015	18.0		-
**New-born mortality**			
Deaths with 28 days (0-27 days)	2.2**		-
Deaths within 7 days (0-6 days)	1.9**		-
Stillbirths	1.19		-
Deaths within 7 days (0-6 days) +Stillbirths	3.1		-
Late neonatal mortality (7-27 days)	0.32***		

Note: ** the denominator excludes stillbirths (128), ***the denominator excludes those who died in the first seven days of live + stillbirths

For peer review only

BMJ Open

The mediation role of low birth weight on the factors associated with new-born mortality and the moderation role of institutional delivery in the association of low birth weight with new-born mortality in a resource-poor setting

Journal:	*BMJ Open*
Manuscript ID	bmjopen-2020-046322.R1
Article Type:	Original research
Date Submitted by the Author:	08-Apr-2021
Complete List of Authors:	Kananura, Rornald; London School of Economics and Political Science, Department of International Development; Makerere University School of Public Health, Department of Health Policy Planning and Management
**Primary Subject Heading**:	Epidemiology
Secondary Subject Heading:	Public health, Health policy
Keywords:	SOCIAL MEDICINE, Community child health < PAEDIATRICS, EPIDEMIOLOGY, PUBLIC HEALTH

SCHOLARONE™
Manuscripts

I, the Submitting Author has the right to grant and does grant on behalf of all authors of the Work (as defined in the below author licence), an exclusive licence and/or a non-exclusive licence for contributions from authors who are: i) UK Crown employees; ii) where BMJ has agreed a CC-BY licence shall apply, and/or iii) in accordance with the terms applicable for US Federal Government officers or employees acting as part of their official duties; on a worldwide, perpetual, irrevocable, royalty-free basis to BMJ Publishing Group Ltd ("BMJ") its licensees and where the relevant Journal is co-owned by BMJ to the co-owners of the Journal, to publish the Work in this journal and any other BMJ products and to exploit all rights, as set out in our licence.

The Submitting Author accepts and understands that any supply made under these terms is made by BMJ to the Submitting Author unless you are acting as an employee on behalf of your employer or a postgraduate student of an affiliated institution which is paying any applicable article publishing charge ("APC") for Open Access articles. Where the Submitting Author wishes to make the Work available on an Open Access basis (and intends to pay the relevant APC), the terms of reuse of such Open Access shall be governed by a Creative Commons licence – details of these licences and which Creative Commons licence will apply to this Work are set out in our licence referred to above.

Other than as permitted in any relevant BMJ Author's Self Archiving Policies, I confirm this Work has not been accepted for publication elsewhere, is not being considered for publication elsewhere and does not duplicate material already published. I confirm all authors consent to publication of this Work and authorise the granting of this licence.

For peer review only

The mediation role of low birth weight on the factors associated with new-born mortality and the moderation role of institutional delivery in the association of low birth weight with new-born mortality in a resource-poor setting

Rornald Muhumuza Kananura [1, 2]

1. London School of Economics and Political Science, Department of International Development, Houghton St, London, WC2A 2AE
2. Makerere University School of Public Health, Department of Health Policy Planning and Management, New Mulago Complex, Kampala, Uganda

Corresponding author

Rornald Muhumuza Kananura, r.m.kananura@lse.ac.uk, +447985274523

For peer review only

For peer review only - http://bmjopen.bmj.com/site/about/guidelines.xhtml

Abstract

Objectives

To assess Low Birth Weight (LBW)'s mediation role on the factors associated with New-born Mortality (NM), including stillbirth and the role of institutional delivery in the association between LBW and NM.

Design and participants

I used the 2011-2015 event histories health demographic data collected by Iganga-Mayuge Health Demographic and Surveillance Site (HDSS). The dataset consisted of 10,758 registered women whose birth occurred at least 22 weeks of the gestation period and records of new-borns' living status 28 days after delivery.

Setting

The Iganga-Mayuge HDSS is in eastern Uganda, which routinely collects health and demographic data from a registered population of at least 100,000 people.

Outcome measure

The study's key outcomes or endogenous factors were Perinatal Mortality (PM), Late NM, and LBW (mediating factor).

Results

The factors that were directly associated with PM were LBW (Odds Ratio(OR)=2.55, 95% confidence interval(95%CI) =1.15-5.67)), maternal age of 30+ (OR=1.68, 95%CI=1.21-2.33), rural residence(OR=1.38, 95%CI=1.02-1.85), mothers with previous experience of NM(OR=3.95, 95%CI=2.86-5.46), and mothers with no education level(OR=1.63, 95%CI=1.21-2.18). Multiple births and mother's prior experience of NM were positively associated with NM at a later age. Institutional delivery had a modest inverse role in the association of LBW with PM. LBW mediated the association of PM with residence status, mothers' previous NM experience, multiple births, adolescent mothers, and mothers marital

For peer review only - http://bmjopen.bmj.com/site/about/guidelines.xhtml

status. Of the total effect attributable to each of these factors, LBW mediated +25%, +22%, +100%, 25%, and -38% of rural resident mothers, mothers with previous experience of new-born or pregnancy loss, multiple births, adolescent mothers, and mothers with partners, respectively.

Conclusion

LBW mediated multiple factors in the NM pathways, and the effect of institutional delivery in reducing mortality among LBW new-borns was insignificant. The findings demonstrate the need for a holistic life-course approach that gears the health systems to tackle NM.

Keywords: *new-born; low birth weight; institutional delivery; pathway, Sub-Saharan Africa; Uganda*

Strengths and limitations

- The study used a large event history health demographic data that had never been exploited to study perinatal and new-born health.
- The gestation age data could indicate if delivery was premature or otherwise and if a pregnancy loss/death was a stillbirth or otherwise were not collected during birth registration.
- There was no information on the maternal morbidities and complications that lead to different birth outcomes, which could have been used to understand their effect along the path.
- There was a considerable proportion of missingness for some of the variables addressed through multiple imputations to minimise the missingness bias.

For peer review only - http://bmjopen.bmj.com/site/about/guidelines.xhtml

Word count: 5287

Introduction

The evidence is clear that the availability of maternal and new-born life-saving technologies and effective interventions have worked towards reducing new-born mortality in developed countries and other developing countries [1–3]. However, new-born mortality continues to have the largest portion of under-five mortality (47%) and has remained unacceptably high in Sub-Saharan Africa (SSA) [4–7]. Of the 2.5 and 2.4 million new-born deaths in 2018 and 2019, respectively, 42% was a portion of SSA in both years of reporting [4,5]. The neonatal mortality rate (NMR) in SSA has persistently remained unacceptably high at an average of 27-28 per 1000 live births [4–7]. At least 75% of the new-born mortality occurs within the first week of life [8], close to 55% within 24 hours [8]. These rates could be underestimated as stillbirths are normally misclassified because of weak documentation systems in SSA [9].

The high proportion of new-born mortality has been attributed to Low Birth Weight (LBW) [10,11] and their related consequences [12]. The direct effect of the socio-economic and demographic factors such as wealth, education level, and maternal age on new-born mortality indicated in existing frameworks [13,14] has been widely studied; however, evidence of how these could be mediated by LBW is elusive. For instance, a direct effect of adolescent age and advanced maternal age on neonatal mortality has been observed in some studies [13–16]. However, maternal age extremes are associated with the increased risk of LBW and other adverse complications [17–23], ultimately leading to new-born mortality. Regarding socio-economic factors, educated and less poor women are more likely to access better maternal health prevention services that control LBW [24–27] and can afford to incur an extra bill, aside from what is freely provided by the government [28,29]. Other LBW contributors include

For peer review only - http://bmjopen.bmj.com/site/about/guidelines.xhtml

morbidity in pregnancy (syphilis and malaria), pre-pregnancy and pregnancy malnutrition exposure, and exposure to environmental factors, particularly indoor air pollution [10,19,30], which are common among vulnerable populations. The scarcity of evidence on how LBW mediates new-born mortality limits the design of moderating interventions. Understanding the mediating role of LBW in the association of new-born mortality with socio-economic, demographic and other individual factors may contribute to generating clinical and community-based interventions that focus on women's health before and after pregnancy; and after delivery.

Further, access to health services is the most effective intervention in preventing maternal morbidities and controlling LBW and other complications' consequences [1,2]. Attending the recommended number of antenatal care exposes the mother to the interventions for identifying and managing pregnancies [31] that may lead to LBW. Additionally, access to ANC exposes the mother-foetus dyad to preventions and care interventions that reduce maternal morbidities associated with the increased likelihood of LBW and prematurity. The first day of life is vital for the survival of new-born, and thus, access to quality of care within labour or delivery time is crucial. During childbirth, women should be attended to by skilled health workers, who should assess the labour complications, including obstructed labour conditions that may need emergency services such as caesarean-section [32–35]. After delivery, the new-borns should be screened for life-threatening signs, including LBW and asphyxia, to benefit from health interventions such as resuscitation for asphyxia and corticosteroid treatment administration/kangaroo mother care for LBW [1,2,34,36]. Notably, reaching the health facilities in the SSA context does not guarantee access to the required services [24,37] because of multiple (concurrent) barriers that impede access to needed services. The health facility-based interventions could be performed inappropriately or too late, or women and new-borns may fail to access the necessary interventions at all because of inadequacies in supplies and

For peer review only - http://bmjopen.bmj.com/site/about/guidelines.xhtml

skilled health workers [3,38]. With the high increasing rates of LBW and preterm birth vis-à-vis the stagnation of high neonatal mortality rates in SSA despite the increase in health facility delivery, evidence on how the health facility deliveries moderates the survival of LBW new-borns is warranted.

The above discussion highlights the role of LBW in mediating the association of some of the factors with new-born mortality and the role of institutional delivery in the relationship between LBW and new-born mortality. However, the pathways and mechanisms of the interactions are unclear, particularly in the sub-Sharan African context. In this study, I analyse the Iganga-Mayuge Health and Demographic Surveillance Site (HDSS) data to assess the LBW's mediation role on the factors associated with new-born mortality and the role of institutional delivery in the association between LBW and new-born mortality. These data have not been exploited in studying new-born survival in Uganda and other countries that share the same context.

Methods

Data

In this study, I used the 2011-2015 Event Histories (EH) health demographic data collected by Iganga-Mayuge Health Demographic and Surveillance Site (HDSS). The EH dataset consisted of 10,758 registered women whose birth occurred at least 22 weeks of the gestation period and records of new-borns' living status 28 days after delivery. The HDSS data are collected in the eastern region of Uganda, covering at least 185 villages in seven sub-counties of Iganga and Mayuge districts. The HDSS routinely collects data on pregnancies, births, migrations, and mortality from a registered population of at least 100,000 people. Community health workers who are based at a village level notify and report all events, and thereafter, the HDSS Field Assistants follow up all the reported events for actual documentation using standard tools. The analysis was limited to births among residents, in-migrants who stayed in the HDSS for at least six months and out-migration that had stayed outside the HDSS within

For peer review only - http://bmjopen.bmj.com/site/about/guidelines.xhtml

six months. The definition of an HDSS resident is someone who has stayed within the HDSS for at least six months, and out-migrants remain the HDSS residents within six months of out-migration. To date, such large event history data in a resource-poor setting have not been used for this kind of studies.

Study variables

The study's key outcomes or endogenous factors that were used to describe the perinatal and new-born mortality or survival system are perinatal mortality, late neonatal mortality and LBW (mediating factor). Perinatal mortality includes deaths within seven days after birth and all stillbirths, while late neonatal mortality is defined as death within 21 days after the first week of survival (7-28 days)[39,40] – describing the timing of death within the new-born survival system. LBW – defined as a birth weight that is less than 2.5 Kgs at birth [41] explains how new-born survival could be affected indirectly by other factors through birth weight.

The new-born adverse outcomes' exogenous variables were categorised as maternal and household factors (having a partner, maternal education level, maternal age, household wealth index), child individual risk factors (childbirth order, birth category – multiple or singleton, child's sex), institutional delivery, mother's previous experience of new-born loss or stillbirth, place of residence (rural or urban), and birth season (in annual quarters). Having a partner was categorised as a dummy variable for 1 – having a partner (those who were either married or cohabiting) and 0 – having no partner (those who were single or widowed). The wealth indices 3-4 were considered less poor, and wealth indices1-2 were considered poorer. The wealth index was generated using principal component analysis, and the items were household assets, household roof structure, household floor structure, and household wall structure. Birth order was categorised as 1 – first order, 2 – 2nd to 4th order and 3 – 5th order. Education was categorised as no education attained, primary level attained, and the post-primary level attained; however, education level of at least primary was related to the reduced risks of

For peer review only - http://bmjopen.bmj.com/site/about/guidelines.xhtml

perinatal and late neonatal mortality, so I later made it as a single dummy variable of no education (0 – at least primary level attained and 1 – no education level at all). Maternal age was grouped as <20 years, 20-29 years and 30 years and above based on the non-parametric analysis (Figure 1 in Supplement 1).

Data cleaning and organisation

Data analysis and cleaning such as categorisation and removing of duplicates were done in STATA v.15. An initial examination of the relationship between the key study independent variables and study outcomes was performed. Non-parametric generalised additive modelling on outcome variables and maternal age relationship was also done to guide the variable classification (Figure 1 in Supplement 1), assuming a non-linear relationship between age and study outcomes.

Multicollinearity between the independent variables was considered at the cut-off point of Variance Inflation Factor (VIF) <10 [42], and there was no evidence of potential multicollinearity as each variable's VIF was less than 6 (Table 1 in Supplement 1). However, because of strong correction between the multiple birth and LBW, multiple birth was dropped under the perinatal mortality multivariable model, while LBW was dropped under the late neonatal mortality multivariable model. Because of the homogeneity across the study clusters, such as villages and sub-counties within the HDSS, the clustering or multilevel modelling approach could not yield a variation across these clusters. Nonetheless, the use of a fixed-effect approach with the inclusion of variables such as place of residence (urban or rural) and household wealth index as key variables of interest provides an insight into the variation in new-born mortality across the place of residence and households.

Multiple imputations for missingness and sensitivity analysis

The extent and pattern of missing data were scrutinised to guide the modelling strategy. Birth weight was missing among 27%, 19% for wealth index, and <1% for other variables. To

For peer review only - http://bmjopen.bmj.com/site/about/guidelines.xhtml

check if the missingness status was missing completely at random or missing at random, I ran a model of missing dummies (0 – not missing and 1 – missing), controlling for variables that I expected to increase the probability of missingness. For birth weight, I found that the likelihood of missing was highly associated with those who did not deliver in the health facilities and those who were born as stillbirths or died immediately after birth (Table 2 in Supplement 1) – indicating a possibility of birth weight missing at random. Similarly, the likelihood of wealth index was highly associated with the maternal age of less than 20 years (Table 2 in Supplement 1) – indicating a possibility of wealth index missing at random. Given the determinants of missingness, I ran multiple imputations (m=100), controlling for identified factors that contribute to the likelihood of missingness and all other variables (as auxiliary) in the dataset. The multiple imputation method has been indicated as an important approach for minimising the missingness bias [43], and this does not depend on the magnitude of missingness [44,45]. After imputation, LBW was found to be underestimated for completed cases data, while the household's poor status was found to be overestimated for completed cases data (Figure 2 in Supplement 1).

Additionally, considering new-born mortality as a rare event, I ran a model for rare events known as the firth logistic model [46] to assess the sample size bias for perinatal mortality, late neonatal mortality, and LBW coefficient estimates. Comparing the firth logit model results with the logistic regression model, I found that the results were consistent (Table 3 in Supplement 1). I later decided to use logistic modelling because of the less time it takes while running imputed data than the firth logit.

Statistical modelling

The analysis was guided by this study's new-born framework (Figure 3 in Supplement 1) that was generated based on the available variables in the dataset and literature on the factors associated with new-born mortality and low birth weight. The outcome variables (LBW,

For peer review only - http://bmjopen.bmj.com/site/about/guidelines.xhtml

prenatal mortality, late neonatal mortality) were in the form of binary, with 1 – indicating the presence of outcome exposure and 0 – the absence of outcome exposure. I, therefore, used a generalised linear modelling approach with a logit link function. To examine the new-born mortality pathway, several analysis steps were applied. Bivariate model(s) with all covariates on the perinatal, late mortality and LBW as outcome variables were performed to identify the variables that would significantly affect outcomes of interest in the adjusted models. The results were reported in terms of odds ratios (OR) and 95% confidence interval (CI). The level of significance was determined at a p-value of <=0.05. Details on the analysis of the mediation effect of LBW on new-born mortality are indicated in Supplement 2.

Patient and public involvement

There was no patient involvement in the study.

Results

Participants' characteristics

Of the total registered births between 2011-2015, 64% were rural residents (Supplement 3). The average maternal age was 27.3 years (standard deviation=±6.5), and 14% were adolescent mothers. Overall, 84% of deliveries occurred in health facilities, of which 15% occurred in private health facilities (Supplement 3). Regarding maternal socio-economic, 86% of the mothers had attained at least primary level education, 53% belonged to a wealth index of 3-5, and 86% were staying with a partner or married (Supplement 3).

Perinatal mortality, neonatal mortality, and LBW estimates

The overall perinatal mortality rate and stillbirth rate within seven days after delivery (0-6) in the five years preceding 2016 were 31 and 12 per 1000 total births, respectively (Supplement 1). Of the total prenatal mortality cases, death within 24 hours (0-1 days +

For peer review only - http://bmjopen.bmj.com/site/about/guidelines.xhtml

stillbirths) accounted for 83%, while death within 1-6 days accounted for 30.8% (Figure 1). Similarly, the overall neonatal mortality rate in the five years preceding 2016 was 22 per 1000 live births. Of the neonatal mortality cases, 62% were deaths within 24 hours of life (0-1 day), and 14 per 1000 live births were deaths within 7-27 days (Figure 1).

Comparing the neonatal and perinatal mortality across five years of birth and death outcome registration, we see a slight change in the perinatal mortality curve between 2011-2014, with the mortality rate reducing from 31-28 per 1000 total birth and gaining its position in the subsequent years (Figure 2). The new-born mortality within day 0-1 has also not significantly changed (27 per 1000 in 2011 versus 26 per 1000 in 2015). Similarly, slight changes in neonatal mortality rates were observed between 2013 and 2015, with the rates increasing by three units between 2013-2014 and reducing by six units in the subsequent year (Figure 2). The new-born mortality within day 1-6 has also not significantly changed (10 per 1000 in 2011 versus 11 per 1000 in 2015). Regarding the LBW, we observe a linear trend between 2011-2012 that was later reduced by three units in the subsequent year – remaining uniform for the rest of the reporting years (Figure 2).

Perinatal mortality, neonatal mortality, and LBW distribution by social-economic and demographic characteristics

I examined the study's LBW distribution, perinatal and neonatal mortality by socio-economic and demographic characteristics. LBW was higher among adolescent aged women than those aged 20 years and above, unmarried women, and rural area residents (Figure 3). Perinatal mortality was higher among those who delivered in the community and public health facilities. For neonatal mortality, a slightly lower death among married, urban residents and deliveries in the private health facilities was observed (Figure 3). Additionally, LBW and neonatal mortality were observed to be higher among those whose mothers had ever experienced pregnancy loss and those born as multiple births (Figure 4). The previous

For peer review only - http://bmjopen.bmj.com/site/about/guidelines.xhtml

experience of pregnancy loss was also related to increased cases of perinatal mortality (Figure 4). The potential factors for perinatal mortality, late neonatal mortality and LBW are investigated in the subsequent sections.

The perinatal mortality pathways

To describe the perinatal mortality pathway, I provide the results in two tables (Table 1 and Table 2). Table 1 indicates how the association of LBW with perinatal mortality changes with institutional delivery and the inclusion of other factors. Table 2 shows the indirect factors associated with perinatal mortality via LBW. At the bivariable level, the crude odds of perinatal mortality were slightly higher for facility deliveries relative to those conducted in the community (Odds Ratio (OR)=1.01, 95% confidence interval (95%CI)=0.80-1.28) (Table 1). Also, at the bivariable level, the crude odds of perinatal mortality were higher among LBW babies relative to those weighing 2.5Kgs+ (OR=2.54, 95%CI=1.74-3.70). Controlling for health facility delivery and introducing an interaction between LBW, a modest effect of health facility delivery on the effect of LBW was observed (OR=0.91, 95%CI=0.40-2.07) (Table 1). Controlling for other factors in the final model, the odds of perinatal mortality among the LBW new-borns remained almost the same (OR=2.55, 95%CI=1.15-5.67). The other factors that were directly associated with the increased odds of perinatal mortality were being a rural resident (OR=1.38, 95%CI=1.02-1.85), mother's previous experience of perinatal mortality (OR=3.95, 95%CI=2.86-5.46), advanced maternal age (OR=1.68, 95%CI=1.21-2.33) and having no education at all (OR=1.63, 95%CI=1.21-2.18). Birth of 5th order and above was inversely associated with perinatal mortality (OR=0.38, 95%CI=0.17-0.87) (Table 1).

As mentioned earlier, LBW was strongly associated with perinatal mortality and was considered a potential mediating factor for perinatal mortality. Table 2 indicates the magnitude effect of the LBW factors in the study's dataset. The factors that were associated with the increased odds of LBW were adolescent mothers (OR=1.40, 95%CI=1.14-1.71), rural resident mothers (OR=1.40, 95%CI=1.19-1.65), mothers with previous experience of new-born or

For peer review only - http://bmjopen.bmj.com/site/about/guidelines.xhtml

pregnancy loss (OR=1.33, 95%CI=1.01-1.76), babies born as multiple births (OR=3.12, 95%CI=2.31-4.21). Having a partner or being married was inversely associated with LBW (OR=0.76, 95%CI=0.63-0.92) (Table 2). Of the total effect attributable to each of these factors, LBW mediated +25% of rural resident mothers, +25% of adolescent mothers, +22% of mothers with previous experience of new-born or pregnancy loss, +100% of multiple births and -38% of mothers with partners **(**Table 4 in Supplement 1).

For peer review only

Table 1: Underlying determinants of perinatal mortality using 2011-2015 Iganga-Mayuge HDSS event histories data

	Unadjusted		Adjusted 1		Adjusted 2		Fully adjusted	
	**OR**	**95%CI**	**OR**	**95%CI**	**OR**	**95%CI**	**OR**	**95%CI**
**Childbirth weight**								
<2.5 Kgs	1.00	-	1.00	-	1.00		1.00	-
25 Kgs+	2.54	[1.74-3.70]***	2.54	[1.75-3.71]***	2.73	[1.21-6.14]*	2.55	[1.15-5.67]*
**Place of delivery**								
Community	1.00	-	1.00	-	1.00	-	1.00	-
Health facility	1.01	[0.8-1.28]	1.04	[0.82-1.32]	1.06	[0.82-1.37]	1.16	[0.89-1.53]
**Place of delivery and LBW interaction [a]**								
<2.5 Kgs and health facility delivery interaction	-	-	-	-	0.91	[0.4-2.07]	0.91	[0.40-2.06]
**Annual birth quarters**								
1	1.00	-	-	-	-	-	1.00	-
2	1.08	[0.78-1.49]	-	-	-	-	1.07	[0.77-1.49]
3	1.26	[0.93-1.72]	-	-	-	-	1.23	[0.9-1.68]
4	1.20	[0.87-1.64]	-	-	-	-	1.20	[0.87-1.66]
**Maternal education level**								
Primary+	1.00	-	-	-	-	-	1.00	-
None	1.69	[1.27-2.25]***	-	-	-	-	1.63	[1.21-2.18]***
**Maternal age**								
<20	1.12	[0.83-1.53]	-	-	-	-	1.08	[0.50-2.33]
20—29	1.00	-	-	-	-	-	1.00	-
30+	1.48	[1.18-1.85]***	-	-	-	-	1.68	[1.21-2.33]***
**Childbirth order**								
1	0.79	[0.62-1.01]	-	-	-	-	1.05	[0.69-1.60]
2—4	1.00	-	-	-	-	-	1.00	-
5th+	0.51	[0.23-1.14]	-	-	-	-	0.38	[0.17-0.87]*
**Place of residence**								
Urban	1.00	-	-	-	-	-	1.00	-

For peer review only - http://bmjopen.bmj.com/site/about/guidelines.xhtml

	Unadjusted		Adjusted 1		Adjusted 2		Fully adjusted	
	**OR**	**95%CI**	**OR**	**95%CI**	**OR**	**95%CI**	**OR**	**95%CI**
Rural	1.40	[1.1-1.78]**	-	-	-	-	1.38	[1.02-1.85]*
**Marital status**								
No partner	1.00	-	-	-	-	-	1.00	-
Has a partner	0.74	[0.54-1.02]	-	-	-	-	0.75	[0.52-1.07]
**Experienced neonatal or pregnancy loss previously**								
No	1.00	-	-	-	-	-	1.00	-
Yes	4.18	[3.1-5.63]***	-	-	-	-	3.95	[2.86-5.46]***
**Household Wealth**								
Poor(er) (1-2)	1.00		-	-	-	-	1.00	-
Less poor (3-5)	1.10	[0.88-1.38]	-	-	-	-	0.85	[0.65-1.12]
**Child sex**								
Male	1.00	-	-	-	-	-	1.00	-
Female	1.07	[0.82-1.40]	-	-	-	-	1.05	[0.8-1.37]
**First birth order-adolescence age interaction [b]**								
First order and <20 years	-	-	-	-	-	-	1.18	[0.49-2.87]
**First birth order-advanced age interaction [b]**								
First order and 30+ years	-	-	-	-	-	-	0.74	[0.36-1.50]
**Birth category**								
Singleton	1.00	-	-	-	-	-	-	-
Multiple	1.02	[0.5-2.08]	-	-	-	-	-	-

Note: [a] base – community delivery – birth weight 2.5Kgs and above interaction; [b] base - second and above birth order – 30 years of age and below interaction; 95%CI – 95% confidence interval; OR – Odds Ration; *** P<0.001; ** P<0.01; * P<0.05

For peer review only - http://bmjopen.bmj.com/site/about/guidelines.xhtml

Table 2: Perinatal mortality indirect factors mediated through LBW using Iganga-Muyuge HDSS event history data

	Unadjusted		Adjusted	
	**OR**	**95%CI**	**OR**	**95%CI**
**Annual birth quarters**				
1	1.00	-	1.00	-
2	1.11	[0.93-1.34]	1.12	[0.93-1.34]
3	1.05	[0.87-1.27]	1.06	[0.88-1.28]
4	1.03	[0.86-1.24]	1.03	[0.85-1.24]
**Maternal education level**				
Primary+	1.00	-	1.00	-
None	1.14	[0.95-1.37]	1.14	[0.94-1.37]
**Maternal age**				
<20	1.43	[1.2-1.7]***	1.40	[1.14-1.71]***
20—29	1.00	-	1.00	-
30+	0.94	[0.82-1.08]	0.98	[0.84-1.14]
**Childbirth order**				
1	0.91	[0.8-1.04]	0.88	[0.75-1.02]
2—4	1.00	-	1.00	-
5th+	1.03	[0.75-1.41]	0.97	[0.69-1.35]
**Place of residence**				
Urban	1.00	-	1.00	-
Rural	1.39	[1.22-1.59]***	1.40	[1.19-1.65]***
**Marital status**				
No partner	1.00	-	1.00	-
Has a partner	0.71	[0.6-0.85]***	0.76	[0.63-0.92]**
**Experienced neonatal or pregnancy loss previously**				
No	1.00	-	1.00	-
Yes	1.45	[1.11-1.89]**	1.33	[1.01-1.76]*
**Birth category**				
Singleton	1.00	-	1.00	-
Multiple	3.10	[2.31-4.16]***	3.12	[2.31-4.21]***
**Household Wealth**				
Poor(er) (1-2)	1.00	-	1.00	-
Less poor (3-5)	1.16	[1.02-1.32]*	0.92	[0.78-1.08]
**Child sex**				
Male	1.00	-	1.00	-
Female	1.13	[0.99-1.29]	1.12	[0.99-1.28]

Note: 95%CI – 95% confidence interval; OR – Odds Ration; *** P<0.001; ** P<0.01; * P<0.05

For peer review only - http://bmjopen.bmj.com/site/about/guidelines.xhtml

The increase in the odds of late neonatal mortality was associated with previous experience of pregnancy loss or neonatal mortality (OR=3.17, 95%CI= 1.15-8.74) and multiple births (OR=6.93, 95%CI=2.58-18.55) (Table 3).

Table 3: Late neonatal mortality risk factors using 2011-2015 Iganga-Mayuge HDSS event histories data

	Unadjusted		Adjusted	
	**OR**	**95%CI**	**OR**	**95%CI**
**Annual birth quarters**				
1	1.00	-	1.00	-
2	1.55	[0.55-2.91]	1.65	[0.58-4.68]
3	1.63	[0.59-3.02]	1.64	[0.59-4.56]
4	1.43	[0.49-2.72]	1.41	[0.49-4.09]
**Education Level**				
No level of education	1.00	-	1.00	-
Primary education+	1.37	[0.56-2.35]	1.24	[0.51-3.06]
**Maternal age (years)**				
<20	0.87	[0.31-1.66]	1.21	[0.37-3.97]
20-29	1.00	-	1.00	-
30+	1.73	[0.87-2.63]	1.73	[0.81-3.69]
**Childbirth order**				
1st	0.70	[0.34-1.07]	0.88	[0.38-2.04]
2—4	1.00	-	1.00	-
5th+	2.20	[0.67-4.54]	1.89	[0.54-6.61]
**Place of residence**				
Urban	1.00	-	1.00	-
Rural	0.77	[0.38-1.17]	0.82	[0.35-1.95]
**Marital status**				
Has no partner	1.00	-	1.00	-
Has a partner	0.66	[0.27-1.14]	0.54	[0.21-1.43]
**Experienced neonatal or pregnancy loss previously**				
No	1.00	-	1.00	-
Yes	3.60	[1.39-6.46]**	3.17	[1.15-8.74]*
**Birth category**				
Singleton	1.00	-	1.00	-
Multiple	7.71	[2.95-13.85]***	6.93	[2.58-18.55]***
**Household Wealth**				
Poorer (1-2 index)	1.00	-	1.00	-
Less poor (3-5 index)	0.75	[0.37-1.16]	0.72	[0.29-1.74]
**Child sex**				
Male	1.00	-	1.00	-
Female	0.57	[0.28-0.88]	0.55	[0.27-1.12]

For peer review only - http://bmjopen.bmj.com/site/about/guidelines.xhtml

For peer review only

	Unadjusted		Adjusted	
	**OR**	**95%CI**	**OR**	**95%CI**
**Place of delivery**				
Community	1.00	-	-	-
Health facility	1.22	[0.57-1.95]	-	-

Note: 95%CI – 95% confidence interval; OR – Odds Ration; *** P<0.001; ** P<0.01; * P<0.05

Discussion

To the best of my knowledge, this is the first study in a resource-poor setting that seeks to understand how LBW new-born mediates new-born mortality and how the institution delivery may moderate the LBW effect on new-born survival. The analysis was based on an event history health demographic dataset that has not been exploited in studying new-born survival in Uganda and other countries with the same context. The study results provide an insight into new-born survival life-course and health systems interventions, particularly targeting the prevention of LBW occurrences and management of or care for LBW new-borns. In the subsequent sub-sections, I discuss 1) the health facility pathways in affecting the survival of new-borns, and 2) the pathway through which LBW mediate the new-born survival. The results are interpreted, and where possible, recommendations for the design of appropriate interventions are discussed based on the available studies and implementation research evidence in a similar context.

Health facility delivery and neonatal mortality

The study results confirm the persistent high perinatal mortality rates in Uganda, with stillbirths and death within 24 hours contributing the largest share. The modest effect of health facility delivery on the survival of LBW new-borns and the large proportion of new-born death within 24 hours after birth indicates a gap in accessing the required health services. We know that, during labour, women should be attended to by skilled health workers who are knowledgeable and qualified to screen for labour complications such as obstructed labour conditions, which need life-saving emergency services, for instance, caesarean section [32,47].

For peer review only - http://bmjopen.bmj.com/site/about/guidelines.xhtml

However, access to these interventions is usually limited in such resource-limited setting [3]. Further, the substantial effect of LBW on the survival of new-borns is well documented [10,12], but interventions for preventing or controlling the effect of LBW are always inadequately available in such resource-limited settings. For instance, kangaroo mother care and resuscitation that are easy to implement, inexpensive and effective [1,47] for helping such new-borns survival are not universally implemented in most of the health facilities in such settings. Additionally, access to ANC such as screening for maternal morbidities and prenatal interventions may help protect the mother-foetus dyad from maternal morbidities [48] and ultimately reduce the likelihood of adverse outcomes. However, usually, most women do not access such services [37].

Noteworthy is that in this study's setting and sub-Saharan Africa in general, such interventions may fail to work effectively because of community contextual problem, in particular poor transport and referral systems, which contribute to delays and failures in receiving required services. For instance, there is one public hospital (Iganga Hospital) in this study setting that provides Comprehensive Emergency Obstetric and New-born Care services in the district [49]. Because of the long-distance coupled with poor transport and referral systems, women from rural or remote areas usually fail to access the services and those that make it reach when they are in advanced stages of complications. Such could perhaps explain why rural residents were associated with a high risk of perinatal mortality in this study.

Further, in such settings, most women first access services at different care points within the community because of pluralism in healthcare. They are later referred or go to the accredited or higher-level facilities when adverse complications have emerged. Thus, most women who deliver in higher-level facilities often have complications associated with a high likelihood of perinatal mortality. These highlighted challenges could explain why health facility delivery could not contribute substantially to the reduced probability of stillbirth and

For peer review only - http://bmjopen.bmj.com/site/about/guidelines.xhtml

death within 24 hours. Additionally, the rural communities are characterised by cultural and social behaviours that affect access to pregnancy, delivery and new-born care interventions. These findings suggest interventions that simultaneously improve healthcare access coverage and quality of care if the facility interventions match better outcomes.

LBW effect on perinatal and neonatal mortality

LBW was identified as a potential determinant of perinatal mortality. The other determinants of new-born mortality were marital status, maternal age, and maternal education. Having a partner was inversely associated with LBW, which was consistent with another study on the effect of marital status on the birth outcome [50] – implying that it may reduce perinatal mortality indirectly through birth weight. The effect of marital status on child health outcome could be via decision-making power, which could influence the choice of appropriate services. In the study's context and sub-Saharan Africa, men usually possess resources and assets; thus, unmarried or single women access inadequate health services [51]. In addition to the negotiating power with the health workers, married women have support from their spouse in terms of joint income, joint care and joint decision-making, which may contribute to their birth preparedness [52], child nutrition and general health of the household.

Furthermore, the health policy or rule in Uganda that require women to attend maternal services in accompany of their spouses may limit the unmarried counterpart from accessing maternal health facility services [53–55] that would address LBW. Studies done in similar settings have indicated that women would be denied services unless they attended with their husbands. Sometimes, those with husbands would be attended to as a priority [53–55]. While male engagement is a positive factor, it has been found that it could end up being a discriminative strategy that could create stigma among unmarried women, inhibiting them from accessing the required services [54,55]. Inclusive interventions that target all women groups regardless of statuses, such as age and marital status, should be designed.

For peer review only - http://bmjopen.bmj.com/site/about/guidelines.xhtml

Maternal age of 30 years+ was positively associated with perinatal mortality as the adolescence age (15-19 years) was positively associated with LBW – indicating how the tail ends of maternal age are associated with the increased likelihood of new-born mortality. Notwithstanding, the effect of maternal age on perinatal mortality needs to be interpreted with care because of other confounding factors such as birth order or parity. On the first hand, the effect of maternal age of 30 years+ could be related to birth order. Consistent with a study done in India and Bangladesh [56], new-borns of birth order of at least 5 were at lower risk of mortality compared to the first birth order. When an interaction between maternal age and birth order was introduced, the first births among women aged at least 30 years were positively associated with perinatal mortality. On the other hand, we also know that high parity is associated with pregnancy complications such as hypertension in pregnancy, leading to an increased likelihood of adverse birth outcomes. In low and middle-income countries where maternal age at the first delivery begins as early as 10 years [57], by the age of 25-30, most women have experienced at least four pregnancies that would increase the risk of hypertension and ultimately adverse birth outcomes. In this study, 28% and 49% of women aged 20-29 and 30 years respectively had children of 3rd and 4th birth order. These results suggest adolescent pregnancy and fertility control interventions targeting adolescents, newly delivered women and multiparous women. Furthermore, the results suggest clinical screening and treatment for morbidity in pregnancy, which may reduce adverse birth outcomes.

In this study, multiple births were not strongly associated with perinatal mortality but strongly associated with mortality at a later age. Although some studies have indicated multiple births to be highly associated with infant and under-five mortality [58,59], this study highlights that the association may vary by age. The association of multiple births with perinatal mortality in this study has been indicated to be mediated by LBW. The association of multiple births with LBW and preterm births has also been studied in other studies [60,61]. Women with

For peer review only - http://bmjopen.bmj.com/site/about/guidelines.xhtml

multiple births need special care [62,63] in terms of nutrition to produce enough breast milk to feed the new-borns.

Further, multiple new-borns are more likely to be very small new-borns, and appropriate regular postnatal examinations are recommended [60]. However, for marginalised communities and families, such care could be inadequate because of limited resources. In addition, multiple birth children are in most cases preterm or very LBW new-born and thus susceptible to recurrent infections that may need frequent health care services, which are often inadequately available in rural communities. Interventions within community and health facility levels for supporting women with multiple births are thus needed. I also recommend a comprehensive study on the care for multiple births, including small new-borns, beyond the facility interventions, particularly focusing on the burden and the community practices of caring for such new-borns in a resource-limited setting.

Attending at least primary education level was directly associated with the reduced likelihood of perinatal mortality – a finding that has been indicated in another study [64]. The pathway could be better health behavioural practices such as nutrition, better sanitation, and appropriate healthcare access. In most cases, educated women have access to the health information on maternal nutrition, good hygiene practices and maternal danger signs that could have been attained either while at school or through reading media publications. These findings suggest a need to design and implement behavioural interventions that integrate less educated communities. One of the strategies that have been effective in mobilising and sensitising poor and illiterate communities in such resource-limited settings is the use of community health workers, which could be exploited to target such groups with maternal and new-born specific health information. Additionally, interventions that promote girl child education and those that prevent early pregnancies targeting girls and families could increase education level among women in the long run.

For peer review only - http://bmjopen.bmj.com/site/about/guidelines.xhtml

Previous stillbirth and neonatal death experience was related to a high likelihood of perinatal mortality and continued to affect the neonates in the later stage, which is consistent with other studies done in developed countries [65,66]. Women with previous experience of pregnancy loss are susceptible to risk conditions such as preeclampsia, preterm births, intrauterine growth retardation and foetal distress that could affect the health of subsequent pregnancies [67]. Additionally, perinatal mortality recurrence, particularly stillbirths, could be related to chronic maternal conditions [66]. Proper screening for the histories of pregnancy loss and neonatal mortality in addition to the histories of related risk factors such as hypertension, diabetes and epilepsy should be emphasised during the prenatal and labour period.

Study strengths and limitations

The study used a large event history health demographic data that had never been exploited to study perinatal and new-born health. The health and demographic surveillance data have been indicated to have a high statistical power close to 100% for mortality measurement [68]. Nevertheless, this study has some limitations related to the data source and context. First, gestation age data that could show if the birth was premature or otherwise and if the pregnancy loss/death was a stillbirth or otherwise were not documented during birth registration. Second, there was no information on the maternal morbidities and complications that lead to different birth outcomes, which could have been used to understand their effect along the path. Third, there was a considerable proportion of missingness for some of the variables that could lead to bias; however, multiple imputations approach was performed to minimise the missingness bias. Lastly, the study results are generalisable to eastern Uganda's setting. Nonetheless, since most communities in Uganda and sub-Saharan Africa share the same community and health characteristics, these results could guide the design of health systems interventions that address mortality causes and risk factors.

Conclusion

For peer review only - http://bmjopen.bmj.com/site/about/guidelines.xhtml

For peer review only

This manuscript contributes to understanding child mortality by analysing the new-born mortality pathways in a resource-poor setting. The study affirms the role of LBW in mediating the association of socio-economic and demographic factors with new-born mortality. The study has also identified the continued effect of multiple birth and the mother's previous pregnancy loss experiences on new-borns survival at a later age. Further, in addition to the large proportion of mortality that occurs within the first day (24 hours), the study has discovered that institutional deliveries had a modest moderation effect in the association of LBW with new-born mortality.

These results also demonstrate the need for a life-course approach in the design of interventions. These interventions should include control of LBW and management or treatment of LBW new-borns. The LBW control interventions should target the socially and economically disadvantaged women, who include adolescents, unmarried women and rural residents, with information on birth preparedness and care for pregnancies. I propose a postnatal treatment and care package for mothers and health workers throughout the new-born life-course for the LBW new-borns and multiple birth. Intensifying the health systems by replicating known LBW effective strategies such as identifying LBW cases through immediate new-born weighing and their management through kangaroo mother care, better nutrition, and other medical interventions are needed.

Additionally, the effect of institutional delivery in such resources limited setting is complex to understand because of the plural health services' points that women go through before reaching the formal care points. The challenges of inadequate amenities for maternal and new-born health, including qualified staff, may make it difficult for the health facilities to address obstetric emergencies appropriately. I suggest a comprehensive health systems' audit, both internal and external, to provide an insight into the reasons for the insignificant effect of

For peer review only - http://bmjopen.bmj.com/site/about/guidelines.xhtml

institutional deliveries on the survival of LBW new-borns and the large proportion of death within the first day of life.

Acknowledgement

Drs Tiziana Leone and Arjan Gjonca advised on the study's design and frequently reviewed the statistical approaches in addition to reviewing the manuscript. Miss Tryphena Nareeba, Dr Dan Kajungu and Dr Peter Waiswa supported me to access the Iganga-Mayuge HDSS data. Dr 5Peter Waiswa also gave me valuable comments that improved this manuscript. I am also grateful for the HDSS data collection team and the mothers who tirelessly provide such information.

Funding

This study is part of my PhD project that is being funded by the London School of Economics and Political Science. The funder had no role in study design, data collection and analysis, decision to publish, or manuscript preparation.

Contributors

RMK solely did data analysis and writing of the manuscript

Competing Interest statement

None declared.

Data sharing statement

The Iganga-Mayuge Health and Demographic Surveillance Site data are not shared publicly. However, they can be made available to other researchers with approval by the Iganga-Mayuge HDSS research ethics committee. Requests for data access may be sent to Collins Gyezayo at info@muchap.mak.ac.ug.

For peer review only

Ethics statements

This study used anonymised secondary data that were provided and thus the patient consent for publication was not required.

Reference

1 Dickson KE, Simen-Kapeu A, Kinney M V., *et al.* Every Newborn: Health-systems bottlenecks and strategies to accelerate scale-up in countries. *Lancet* 2014;**384**:438–54. doi:10.1016/S0140-6736(14)60582-1

2 Darmstadt, L. G, Bhutta, A. Z, Cousens S, *et al.* Evidence-based, cost-effective interventions: How many newborn babies can we save? *Lancet* 2005;**365**:977–88. doi:10.1016/S0140-6736(05)71088-6

3 Zupan J. Perinatal mortality in developing countries. *N Engl J Med* 2005;**352**:2047–8. doi:10.1056/NEJMp058032

4 United Nations Inter-agency Group for Child Mortality Estimation (UN IGME). Levels & Trends in Child Mortality: Report 2020, Estimates Developed by the UN Inter-agency Group for Child Mortality Estimation. 2020. https://www.unicef.org/media/60561/file/UN-IGME-child-mortality-report-2019.pdf

5 United Nations Inter-agency Group for Child Mortality Estimation (UN IGME). Levels & Trends in Child Mortality: Report 2018, Estimates developed by the United Nations Inter-agency Group for Child Mortality Estimation'. New York: 2018.

6 United Nations Inter-agency Group for Child Mortality Estimation (UN IGME). Levels & Trends in Child Mortality: Report 2017, Estimates Developed by the UN Inter-agency Group for Child Mortality Estimation. New York: 2017. http://www.childmortality.org/files_v21/download/IGME report 2017 child mortality final.pdf

7 United Nations Inter-agency Group for Child Mortality Estimation (UN IGME). Levels & Trends in Child Mortality: Report 2019, Estimates Developed by the UN Inter-agency Group for Child Mortality Estimation. 2019.

8 Sankar MJ, Natarajan CK, Das RR, *et al.* When do newborns die? A systematic review of timing of overall and cause-specific neonatal deaths in developing countries. *J Perinatol* 2016;**36**:S1–11. doi:10.1038/jp.2016.27

9 Reinebrant HE, Leisher SH, Coory M, *et al.* Making stillbirths visible: a systematic review of globally reported causes of stillbirth. *BJOG An Int J Obstet Gynaecol* 2018;**125**:212–24. doi:10.1111/1471-0528.14971

10 Blencowe H, Cousens S, Oestergaard MZ, *et al.* National , regional , and worldwide estimates of preterm birth rates in the year 2010 with time trends since 1990 for selected countries : a systematic analysis and implications. *Lancet* 2012;**379**:2162–72. doi:10.1016/S0140-6736(12)60820-4

11 Lawn JE, Blencowe H, Oza S, *et al.* Every Newborn 2 Every Newborn : progress , priorities , and potential beyond. Published Online First: 2015. doi:10.1016/S0140-6736(14)60496-7

12 Barros FC, Matijasevich A, Domingues MR, *et al.* How many low birthweight babies in low- and middle- income countries are preterm ? *Rev Saúde Pública* 2011;**45**:607–16.dx.doi.org/10.1590/S0034-89102011005000019

13 Sharma V, Katz J, Mullany LC, *et al.* Young Maternal Age and the Risk of Neonatal

For peer review only - http://bmjopen.bmj.com/site/about/guidelines.xhtml

Mortality in Rural Nepal. 2009;**162**:828–35. doi:10.1001/archpedi.162.9.828.Young
14 Sinha S, Sc M, Indian F. Association between maternal age at childbirth and perinatal and under-five mortality in a prospective birth cohort from Delhi. *Indian Pediatr* 2017;**53**:871–7.
15 Kibria GM Al, Burrowes V, Choudhury A, *et al.* Determinants of early neonatal mortality in Afghanistan: An analysis of the Demographic and Health Survey 2015. *Global Health* Published Online First: 2018. doi:10.1186/s12992-018-0363-8
16 Fawole AO, Shah A, Tongo O, *et al.* Determinants of perinatal mortality in Nigeria. *Int J Gynecol Obstet* Published Online First: 2011. doi:10.1016/j.ijgo.2011.01.013
17 Grønvik T, Sandøy IF. Complications associated with adolescent childbearing in Sub-Saharan Africa: A systematic literature review and meta-analysis. *PLoS One* 2018;**13**:1–21. doi:10.1371/journal.pone.0204327
18 Manyeh AK, Kukula V, Odonkor G, *et al.* Socioeconomic and demographic determinants of birth weight in southern rural Ghana : evidence from Dodowa Health and Demographic Surveillance System. *BMC Pregnancy Childbirth* 2016;:1–9. doi:10.1186/s12884-016-0956-2
19 Blencowe H, Krasevec J, de Onis M, *et al.* National, regional, and worldwide estimates of low birthweight in 2015, with trends from 2000: a systematic analysis. *Lancet Glob Heal* 2019;**7**:e849–60. doi:10.1016/S2214-109X(18)30565-5
20 Lean SC, Derricott H, Jones RL, *et al.* Advanced maternal age and adverse pregnancy outcomes: A systematic review and meta-analysis. *PLoS One* Published Online First: 2017. doi:10.1371/journal.pone.0186287
21 Althabe F, Moore JL, Gibbons L, *et al.* Adverse maternal and perinatal outcomes in adolescent pregnancies : The Global Network ' s Maternal Newborn Health Registry study. *Reprod Health* 2015;**12**:S8. doi:10.1186/1742-4755-12-S2-S8
22 Weng YH, Yang CY, Chiu YW. Risk assessment of adverse birth outcomes in relation to maternal age. *PLoS One* Published Online First: 2014. doi:10.1371/journal.pone.0114843
23 Blomberg M, Tyrberg RB, Kjølhede P. Impact of maternal age on obstetric and neonatal outcome with emphasis on primiparous adolescents and older women : a Swedish Medical Birth Register Study. *BMJ Open* 2014;**4**:e005840. doi:10.1136/bmjopen-2014-005840
24 Lohela TJ, Nesbitt RC, Pekkanen J, *et al.* Comparing socioeconomic inequalities between early neonatal mortality and facility delivery: Cross-sectional data from 72 low- and middle-income countries. *Sci Rep* Published Online First: 2019. doi:10.1038/s41598-019-45148-5
25 Ronsmans C, Holtz S, Stanton C. Socioeconomic differentials in caesarean rates in developing countries: a retrospective analysis. *Lancet* Published Online First: 2006. doi:10.1016/S0140-6736(06)69639-6
26 Fenn B, Kirkwood BR, Popatia Z, *et al.* Inequities in neonatal survival interventions: Evidence from national surveys. *Arch Dis Child Fetal Neonatal Ed* Published Online First: 2007. doi:10.1136/adc.2006.104836
27 Barros AJ, Ronsmans C, Axelson H, *et al.* Equity in maternal, newborn, and child health interventions in Countdown to 2015: A retrospective review of survey data from 54 countries. *Lancet* Published Online First: 2012. doi:10.1016/S0140-6736(12)60113-5
28 Austin P, Ntenda M, Chuang K, *et al.* Factors Associated with Infant Mortality in Malawi. *J Exp Clin Med* 2014;**6**:125–31. doi:10.1016/j.jecm.2014.06.005
29 Kayode GA, Adekanmbi VT, Uthman OA. Risk factors and a predictive model for under-five mortality in Nigeria : evidence from Nigeria demographic and health

For peer review only

For peer review only - http://bmjopen.bmj.com/site/about/guidelines.xhtml

survey. *BMC Pregnancy Childbirth* 2012;**12**:10. doi:10.1186/1471-2393-12-10
30 Accrombessi M, Zeitlin J, Massougbodji A, *et al.* What Do We Know about Risk Factors for Fetal Growth Restriction in Africa at the Time of Sustainable Development Goals? A Scoping Review. Paediatr. Perinat. Epidemiol. 2018. doi:10.1111/ppe.12433
31 World Health Organization (WHO). *WHO Recommendation on Antenatal care for positive pregnancy experience*. Geneva, Switzerland: : World Health Organization 2016. doi:ISBN 978 92 4 154991 2
32 Darmstadt, L. G, Marchant T, Claeson M, *et al.* A strategy for reducing maternal and newborn deaths by 2015 and beyond. *BMC Pregnancy Childbirth* 2013;**13**:216. doi:10.1186/1471-2393-13-216
33 Wall SN, Lee ACC, Carlo W, *et al.* Reducing Intrapartum-Related Neonatal Deaths in Low- and Middle-Income Countries-What Works? *Semin Perinatol* 2010;**34**:395–407. doi:10.1053/j.semperi.2010.09.009
34 Salam RA, Mansoor T, Mallick D, *et al.* Essential childbirth and postnatal interventions for improved maternal and neonatal health. *Reprod Health* 2014;**11**:1–17. doi:10.1186/1742-4755-11-S1-S3
35 Campbell S. Fetal macrosomia: A problem in need of a policy. *Ultrasound Obstet Gynecol* 2014;**43**:3–10. doi:10.1002/uog.13268
36 Berkley S, Dybul M, Godal T, *et al.* Integration and innovation to advance newborn survival. *Lancet* 2014;**384**:e22–3. doi:10.1016/S0140-6736(14)60691-7
37 Benova L, Tunçalp Ö, Moran AC, *et al.* Not just a number: examining coverage and content of antenatal care in low-income and middle-income countries. *BMJ Glob Heal* 2018;**3**:e000779. doi:10.1136/bmjgh-2018-000779
38 Goldenberg RL, McClure EM, Saleem S. Improving pregnancy outcomes in low- and middle-income countries. *Reprod Health* 2018;**15**:2017–8. doi:10.1186/s12978-018-0524-5
39 Pathirana J, Muñoz FM, Abbing-Karahagopian V, *et al.* Neonatal death: Case definition & guidelines for data collection, analysis, and presentation of immunization safety data. *Vaccine* 2016;**34**:6027–37. doi:10.1016/j.vaccine.2016.03.040
40 Da Silva FT, Gonik B, McMillan M, *et al.* Stillbirth: Case definition and guidelines for data collection, analysis, and presentation of maternal immunization safety data. *Vaccine* 2016;**34**:6057–68. doi:10.1016/j.vaccine.2016.03.044
41 Cutland CL, Lackritz EM, Mallett-Moore T, *et al.* Low birth weight: Case definition & guidelines for data collection, analysis, and presentation of maternal immunization safety data. *Vaccine* 2017;**35**:6492–500. doi:10.1016/j.vaccine.2017.01.049
42 Hair JF, Black WC, Babin BJ, *et al. Multivariate Data Analysis*. Seventh Ed. London: : Pearson Education Limited 2006.
43 Graham JW, Olchowski AE, Gilreath TD. How many imputations are really needed? Some practical clarifications of multiple imputation theory. *Prev Sci* 2007;**8**:206–13. doi:10.1007/s11121-007-0070-9
44 Madley-Dowd P, Hughes R, Tilling K, *et al.* The proportion of missing data should not be used to guide decisions on multiple imputation. *J Clin Epidemiol* 2019;**110**:63–73. doi:10.1016/j.jclinepi.2019.02.016
45 Lee J, Huber JJ. Multiple imputation with large proportions of missing data: How much is too much? United Kingdom Stata Users' Gr. Meet. 2011. 2011.
46 Coveney J. 'FIRTHLOGIT: Stata module to calculate bias reduction in logistic regression,' Statistical Software Components S456948, Boston College Department of Economics, revised 25 Jul 2015. 2008.
47 Das JK, Kumar R, Salam RA, *et al.* Evidence from facility level inputs to improve quality of care for maternal and newborn health: interventions and findings. *Reprod*

For peer review only - http://bmjopen.bmj.com/site/about/guidelines.xhtml

Health 2014;**11 Suppl 2**:S2. doi:10.1186/1742-4755-11-S2-S2
48 Lincetto O, Mothebesoane-anoh S, Gomez P, *et al.* Antenatal Care: Opprotunities for Africa's Newborns. *Int J Sci Technol Res* 2013;**2**:51–62.
49 Uganda Ministry of Health. National Health Facility Master List 2018. Kampala: 2018. http://library.health.go.ug/publications/health-facility-inventory/national-health-facility-master-list-2017
50 Singh U, Ueranantasun A, Kuning M. Factors associated with low birth weight in Nepal using multiple imputation. 2017;:1–10. doi:10.1186/s12884-017-1252-5
51 Cohen JJ, Blevins M, Mapenzi A, *et al.* Overcoming the perceived barriers to health care access among single mothers in coastal Kenya. *Int J Public Health* Published Online First: 2014. doi:10.1007/s00038-013-0511-0
52 Kaiser JL, Fong RM, Hamer DH, *et al.* How a woman's interpersonal relationships can delay care-seeking and access during the maternity period in rural Zambia: An intersection of the Social Ecological Model with the Three Delays Framework. *Soc Sci Med* 2018;**220**:312–21. doi:10.1016/J.SOCSCIMED.2018.11.011
53 Peneza AK, Maluka SO. 'Unless you come with your partner you will be sent back home': strategies used to promote male involvement in antenatal care in Southern Tanzania. *Glob Health Action* 2018;**11**. doi:10.1080/16549716.2018.1449724
54 Påfs J, Musafili A, Binder-Finnema P, *et al.* 'They would never receive you without a husband': Paradoxical barriers to antenatal care scale-up in Rwanda. *Midwifery* 2015;**31**:1149–56. doi:10.1016/j.midw.2015.09.010
55 Sochas L. Women who break the rules: Social exclusion and inequities in pregnancy and childbirth experiences in Zambia. *Soc Sci Med* 2019;**232**:278–88. doi:10.1016/j.socscimed.2019.05.013
56 Al Kibria GM, Khanam R, Mitra DK, *et al.* Rates and determinants of neonatal mortality in two rural sub-districts of Sylhet, Bangladesh. *PLoS One* 2018;**13**:e0206795. doi:10.1371/journal.pone.0206795
57 Christiansen CS, Gibbs S, Chandra-Mouli V. Preventing early pregnancy and pregnancy-related mortality and morbidity in adolescents in developing countries: The place of interventions in the prepregnancy period. *J Pregnancy* 2013;**2013**:15–20. doi:10.1155/2013/257546
58 Uthman OA, Uthman MB, Yahaya I. A population-based study of effect of multiple birth on infant mortality in Nigeria. *BMC Pregnancy Childbirth* 2008;**8**:1–6. doi:10.1186/1471-2393-8-41
59 Monden CWS, Smits J. Mortality among twins and singletons in sub-Saharan Africa between 1995 and 2014: a pooled analysis of data from 90 Demographic and Health Surveys in 30 countries. *Lancet Glob Heal* 2017;**5**:e673–9. doi:10.1016/S2214-109X(17)30197-3
60 Dudenhausen JW, Maier RF. Perinatale probleme von mehrlingen. *Dtsch Arztebl* 2010;**107**:663–8. doi:10.3238/arztebl.2010.0663
61 Blondel B, Kogan MD, Alexander GR, *et al.* The impact of the increasing number of multiple births on the rates of preterm birth and low birthweight: An international study. *Am J Public Health* 2002;**92**:1323–30. doi:10.2105/AJPH.92.8.1323
62 Heather M W, Selina K W, Therese D, *et al.* Breastfeeding education and support for women with twins or higher order multiples. *Cochrane Database Syst Rev* Published Online First: 2017. doi:10.1002/14651858.CD012003.pub2.www.cochranelibrary.com
63 Montgomery KS, Cubera S, Belcher C, *et al.* Childbirth Education for Multiple Pregnancy. *J Perinat Educ* 2005;**14**:33–8. doi:10.1624/105812405x57561
64 Nattey C, Masanja H, Klipstein-Grobusch K. Relationship between household socio-economic status and under-five mortality in Rufiji DSS, Tanzania. *Glob Health Action*

For peer review only - http://bmjopen.bmj.com/site/about/guidelines.xhtml

2013;**6**:19278. doi:10.3402/gha.v6i0.19278
65 Patterson JA, Ford JB, Morris JM, *et al.* Trends and recurrence of stillbirths in NSW. *Aust N Z J Public Health* 2014;**38**:384–9. doi:10.1111/1753-6405.12179
66 Heazell AEP, Clewlow J. Protecting families from recurrent stillbirth. *BMJ* 2015;**350**:1–2. doi:10.1136/bmj.h3262
67 Getahun D, Lawrence JM, Fassett MJ, *et al.* The association between stillbirth in the first pregnancy and subsequent adverse perinatal outcomes. *Am J Obstet Gynecol* 2009;**201**:378.e1-378.e6. doi:10.1016/j.ajog.2009.06.071
68 Bocquier P, Sankoh O, Byass P. Are health and demographic surveillance system estimates sufficiently generalisable? *Glob Health Action* 2017;**10**. doi:10.1080/16549716.2017.1356621

Figure legends

Figure 1: Perinatal and New-born mortality distribution by days of death
Figure 2: Annual changes in perinatal mortality rates, neonatal mortality rates and LBW
Figure 3: New-born mortality and LBW stratified by maternal socio-economic characteristics, place of residence and place of delivery
Figure 4: New-born mortality and LBW stratified by individual characteristics

For peer review only

For peer review only - http://bmjopen.bmj.com/site/about/guidelines.xhtml

Figure 1: Perinatal and New-born mortality distribution by days of death

I. **Distribution of perinatal mortality by days of death**

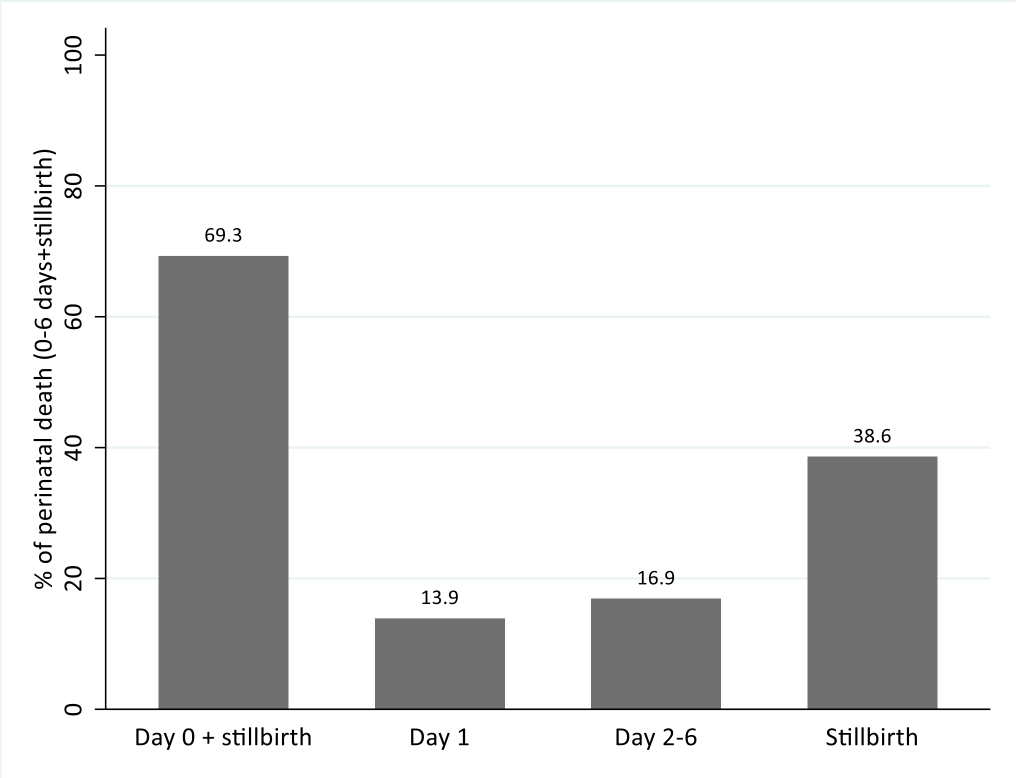

II. **Distribution of neonatal mortality by days of death**

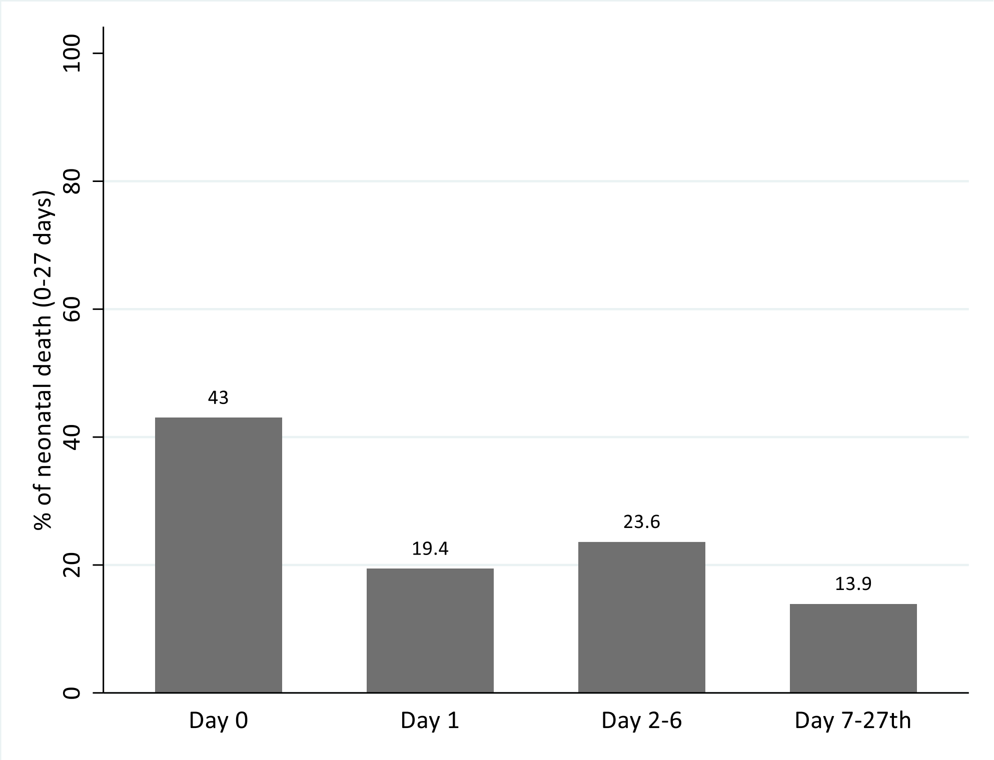

I. Perinatal mortality and Neonatal mortality annual estimates

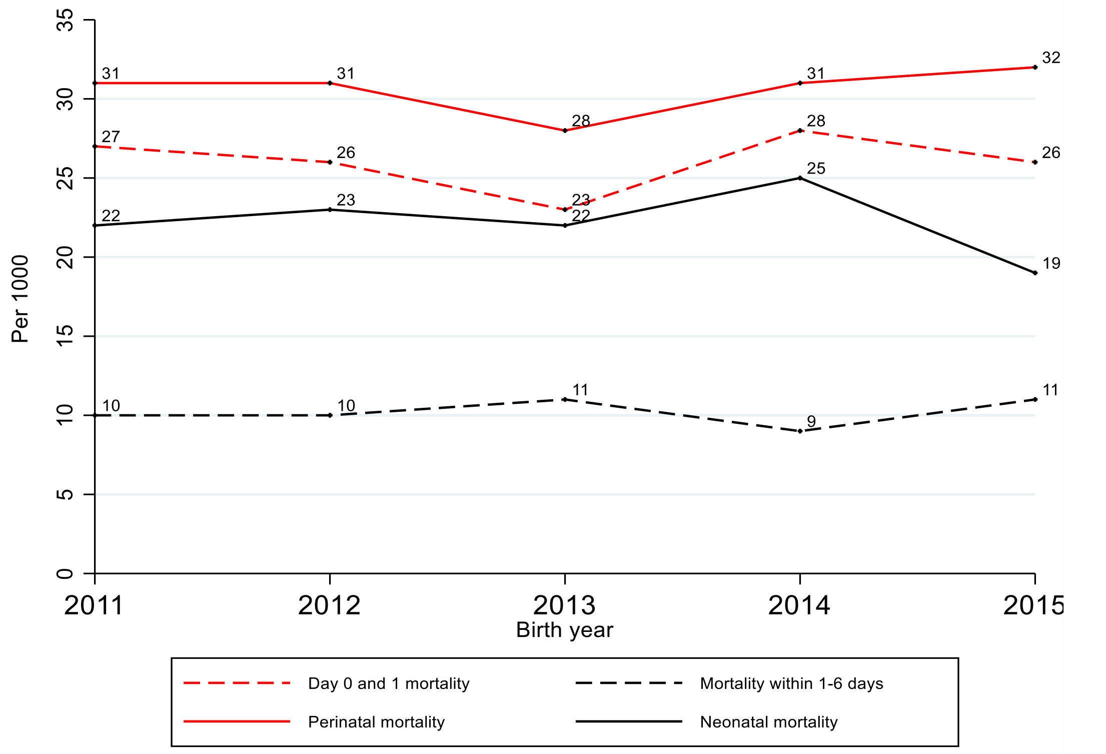

1. *Note:*
2. *Perinatal mortality was calculated as the number of births that resulted in stillbirths or died within 6 days after birth per the number of births (0-6 days + stillbirths).*
3. *Neonatal mortality was calculated as the number of newborns that died within 27 days immediately or during birth (0-27days)*
4. *Mortality within 1-6 days was calculated as death among new-borns who were alive after birthday*

II. Low birth weight annual estimates

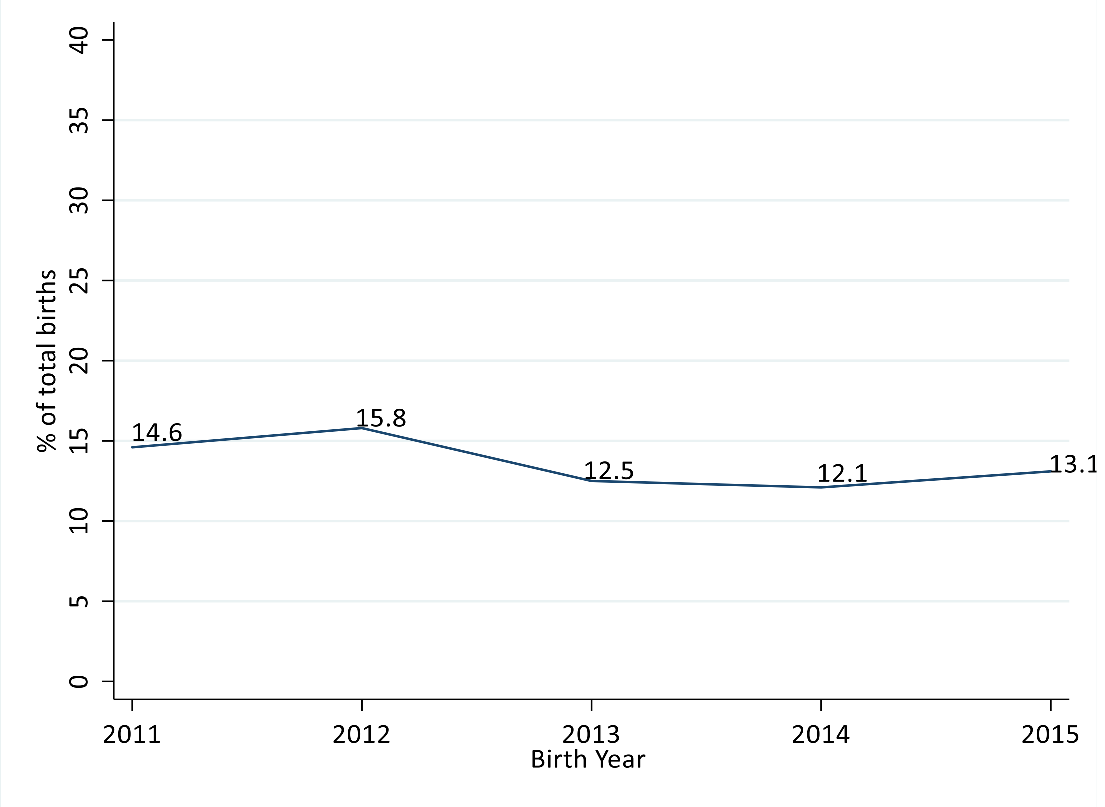

Figure 3: New-born mortality and LBW stratified by maternal socio-economic characteristics, place of residence and place of delivery

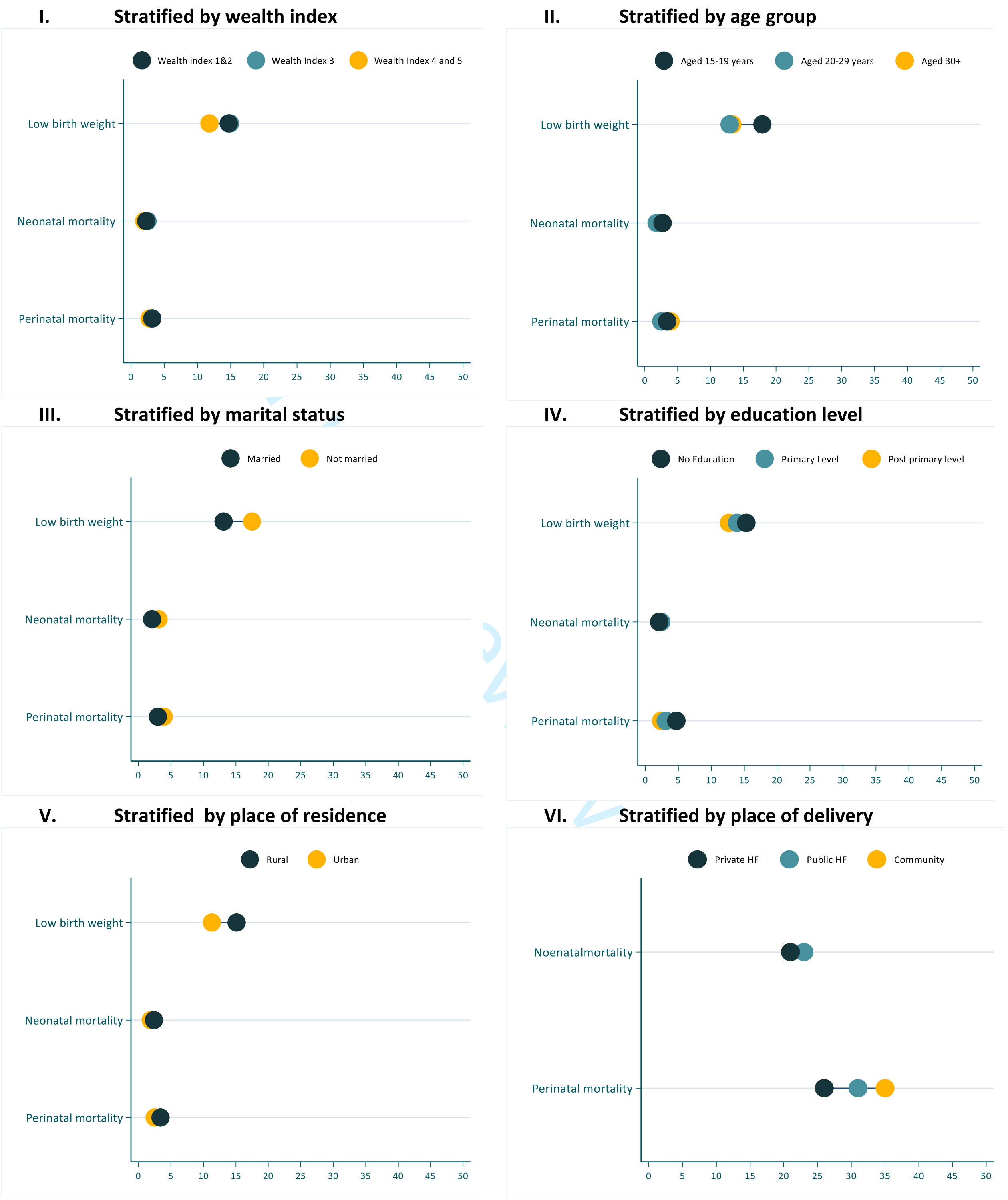

Figure 4: New-born mortality and LBW stratified by individual characteristics

I. Stratification by pregnancy loss previous experience

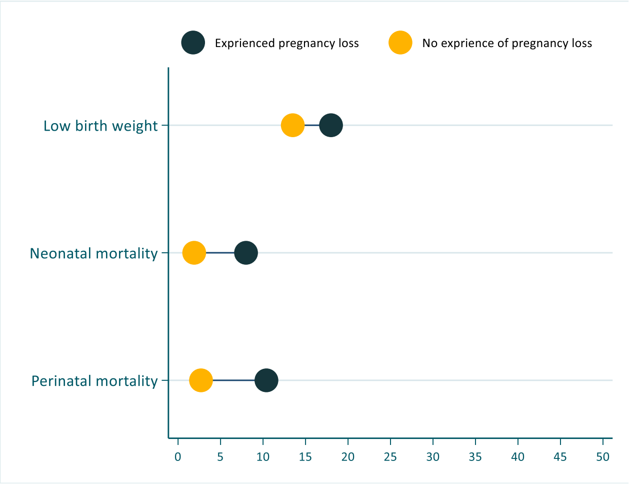

II. Stratification by birth category

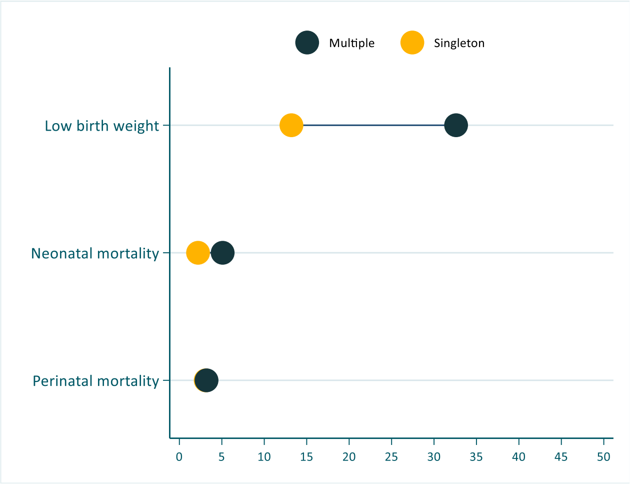

III. Stratification by sex

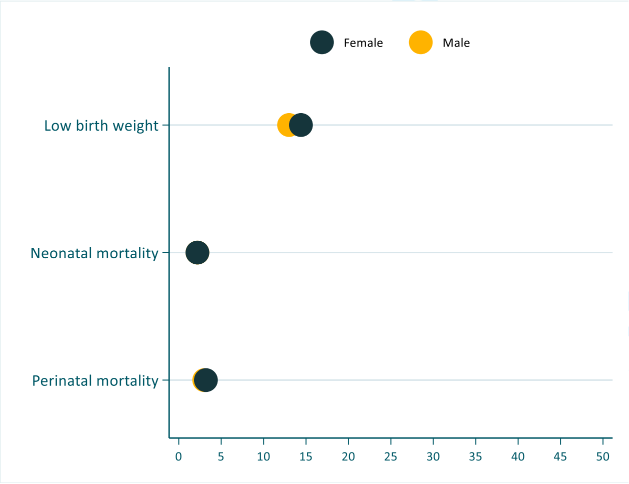

IV. Stratification by birth order

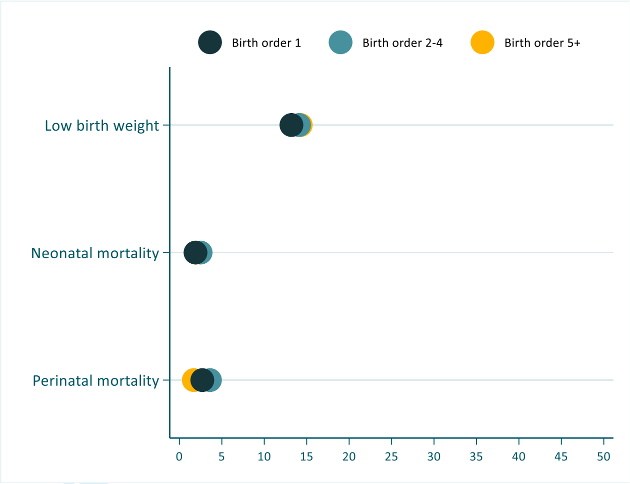

Supplement 1

Table 1: Multicollinearity assessment for perinatal and neonatal mortality variables in the event history dataset

Variable	VIF	SQRT VIF	Tolerance	R-Squared
Birth season in quarters				
2	1.53	1.23	0.6557	0.3443
3	1.54	1.24	0.6481	0.3519
4	1.56	1.25	0.6404	0.3596
Child as Low birth weight	1.43	1.19	0.7006	0.2994
Child low birth weight and delivered in private facility	1.38	1.18	0.7238	0.2762
Child low birth weight and delivered in community	1.4	1.19	0.7118	0.2882
Primary Education	5.04	2.25	0.1982	0.8018
Post Primary Education	5.41	2.33	0.1848	0.8152
Delivered in private health facilities	1.3	1.14	0.7712	0.2288
Delivered in community	1.25	1.12	0.7991	0.2009
Maternal age				
<20 years	1.3	1.14	0.7707	0.2293
30+ years	1.19	1.09	0.8435	0.1565
Birth order				
1	1.39	1.18	0.7208	0.2792
5+	1.06	1.03	0.9397	0.0603
Rural residence	2.23	1.49	0.4477	0.5523
Has a partner	1.19	1.09	0.8412	0.1588
Experienced pregnancy or child loss previously	1.04	1.02	0.9631	0.0369
Multiple birth	1.02	1.01	0.9833	0.0167
Child sex as female	1	1	0.997	0.003
Wealth index				
Index 3	1.26	1.12	0.794	0.206
Index 4 and 5	2.81	1.68	0.3557	0.6443
Mean VIF	1.78			

For peer review only

Table 2: Assessment of missing variable factors for birth weight and wealth index in the event history dataset

	(1) Birth weight missingness	std.Err	(2) Wealth index missingness	std.Err
**Time of death**				
Day 1 and above	1.00	-	-	-
Still birth and day 0	9.137***	(12.47)	-	-
**Place of birth**			-	-
Hospital	1.00	-	-	-
Community	3.343***	(20.14)	-	-
Private	2.680***	(16.17)	-	-
**Place of residence**			-	-
Urban	1.00	-	-	-
Rural	1.779***	(10.28)	0.459***	(-13.56)
**Age group**				
<20 years	1.145	(1.94)	1.226*	(2.44)
20-29	1.00	-	1.00	-
30+	0.947	(-1.05)	0.619***	(-7.41)
**Education level**				
None	1.00	-	1.00	-
Primary	0.759***	(-4.21)	0.985	(-0.11)
Post primary	0.568***	(-7.60)	1.250	(1.68)
**Marital status**				
Has no a partner	-	-	1.00	-
Has a partner	-	-	2.010***	(7.38)

Exponentiated coefficients; t statistics in parentheses * p<0.05, ** p<0.01, *** p<0.001

Table 3: Bivariate analysis comparing logistic and firth logit model using the 2011-2015 Iganga-Mayuge event history data

	Perinatal mortality				Late neonatal mortality			
	Logit		Firth logit		Logit		Firth logit	
	OR	P-value	OR	P-value	OR	P-value	OR	P-value
**Annual birth quarters**								
1	1.00	-	1.00	-	1.00	-	1.00	-
2	1.08	0.63	1.08	0.63	1.55	0.41	1.51	0.42
3	1.26	0.14	1.26	0.14	1.63	0.35	1.58	0.36
4	1.20	0.27	1.20	0.27	1.43	0.51	1.40	0.52
**Childbirth weight**								
<2.5 Kgs	2.52	<0.001	2.53	<0.001	2.37	0.05	2.45	0.04
25 Kgs+								
**Maternal education level**								
None	1.69	<0.001	1.70	<0.001	1.37	0.49	1.46	0.39
Primary+	1.00	-	1.00	-	1.00	-	1.00	-

For peer review only - http://bmjopen.bmj.com/site/about/guidelines.xhtml

For peer review only

	Perinatal mortality				Late neonatal mortality			
	Logit		Firth logit		Logit		Firth logit	
	OR	P-value	OR	P-value	OR	P-value	OR	P-value
**Place of delivery**								
Health facility	1.01	0.91	1.01	0.93	1.22	0.61	1.18	0.67
Community	1.00	-	1.00	-	1.00	-	1.00	-
**Maternal age (years)**								
<20	1.12	0.46	1.13	0.42	0.88	0.80	0.97	0.95
20—29	1.00	-	1.00	-	1.00	-	1.00	-
30+	1.48	<0.001	1.48	<0.001	1.73	0.12	1.74	0.11
**Childbirth order**								
1	0.80	0.07	0.80	0.07	0.70	0.32	0.71	0.33
2—4	1.00	-	1.00	-	1.00	-	1.00	-
5th+	0.46	0.06	0.49	0.07	2.20	0.20	2.52	0.10
**Place of residence**								
Urban	1.00	-	1.00	-	1.00	-	1.00	-
Rural	1.40	0.01	1.39	0.01	0.77	0.45	0.76	0.43
**Marital status**								
No partner	1.00	-	1.00	-	1.00	-	1.00	-
Has a partner	0.74	0.07	0.74	0.07	0.63	0.31	0.60	0.24
**Experienced neonatal or pregnancy loss previously**								
No								
Yes	4.18	0.00	4.20	<0.001	3.60	0.01	3.89	0.00
**Birth category**								
Singleton	1.00	-	1.00	-	1.00	-	1.00	-
Multiple	1.02	0.96	1.08	0.83	7.70	<0.001	8.31	0.00
**Child sex**								
Male	1.00	-	1.00	-	1.00	-	1.00	-
Female	1.06	0.68	1.06	0.68	0.57	0.12	0.58	0.12
**Household Wealth**								
Poor(er) (1-2)	1.10	0.43	1.10	0.43	0.73	0.39	0.75	0.41
Less poor (3-5)	1.00	-	1.00	-	1.00	-	1.00	-

Table 4: Indirect pathways of the association between LBW and perinatal mortality.

	Indirect effect (IE)	Se	P-value*	Total effect (TE)	Percent (IE/TE)
Maternal age of <20 years	0.03	0.12	0.84	0.11	24.98
Rural residence	0.11	0.06	0.06	0.43	25.21
Having a partner	0.08	0.05	0.14	-0.21	-37.95
Experience of previous pregnancy loss	0.39	0.19	0.04	1.77	22.19
Multiple birth	1.14	-	-	1.14	100.00

Note: Se – standard error; *- 0.05 level of significancy

For peer review only - http://bmjopen.bmj.com/site/about/guidelines.xhtml

For peer review only

Figure 1: Relationship between age and birth outcomes using for age categorization using event histories data

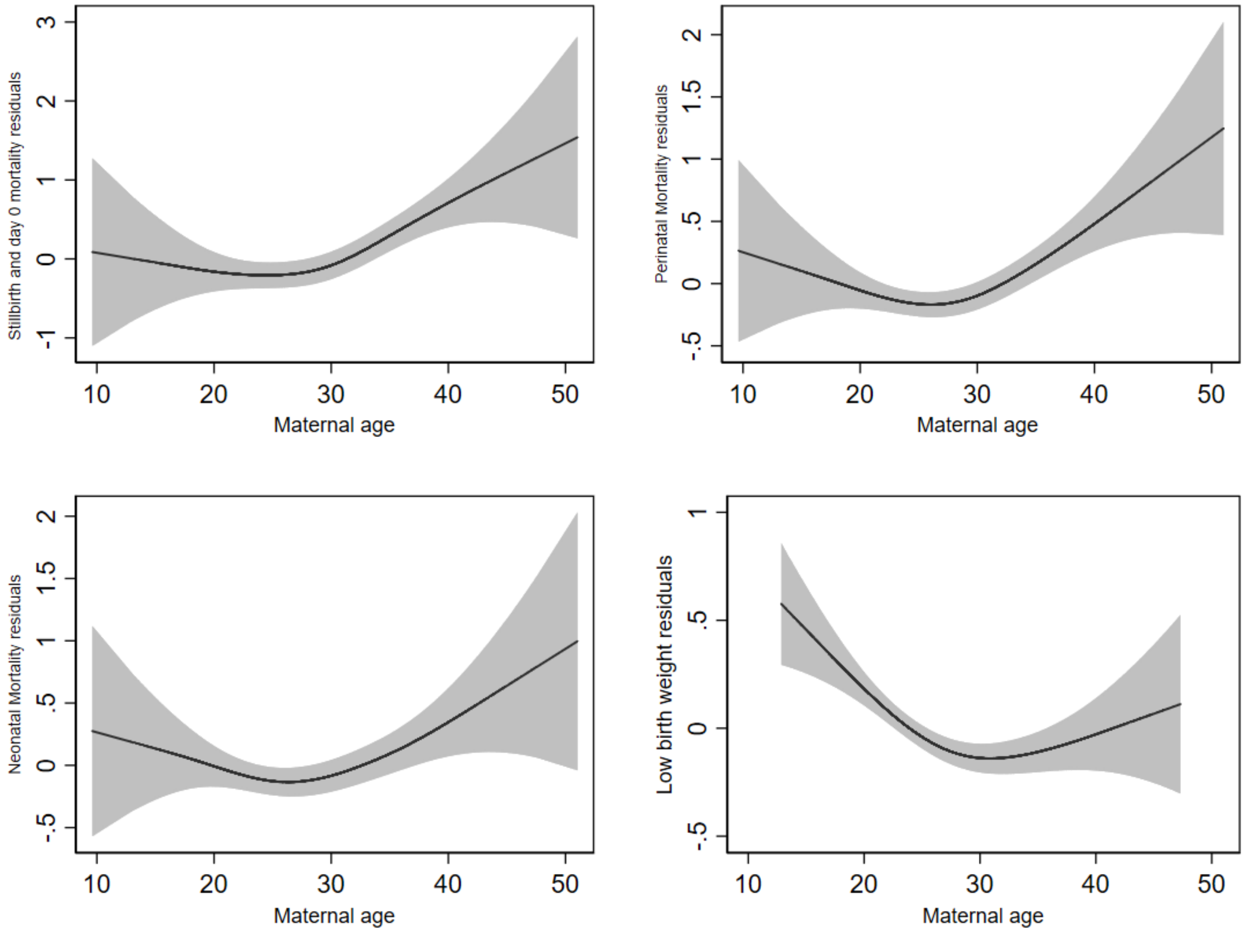

For peer review only - http://bmjopen.bmj.com/site/about/guidelines.xhtml

Figure 2: Comparing the LBW and household poor status for imputed and completed cases

I. LBW estimates for imputed and completed cases analysis

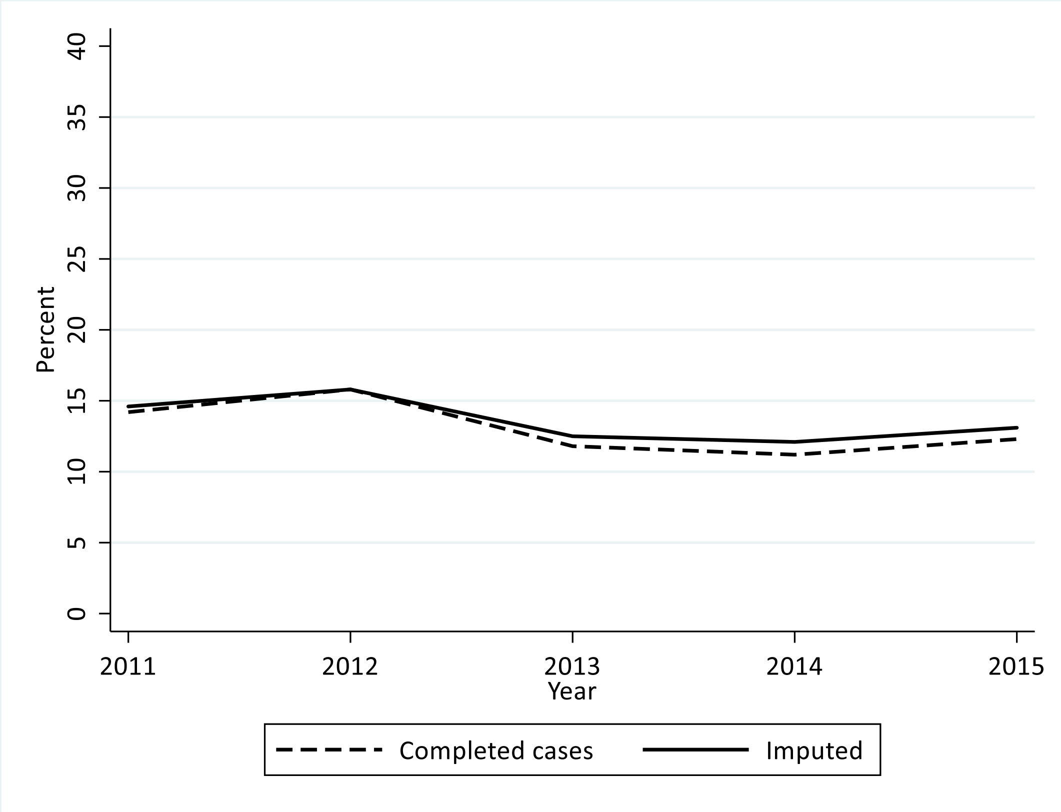

II. Household poor status (wealth index 1 and 2) estimates for imputed and completed cases analysis

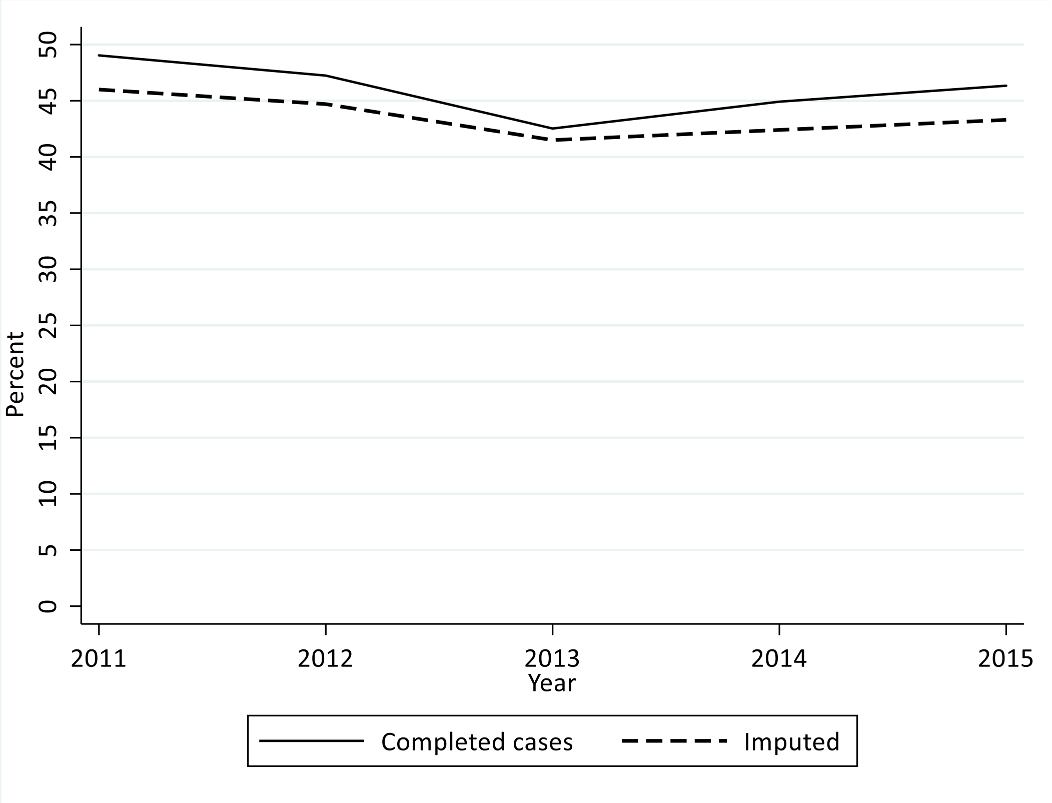

For peer review only - http://bmjopen.bmj.com/site/about/guidelines.xhtml

Figure 3: New-born mortality analysis pathways' framework

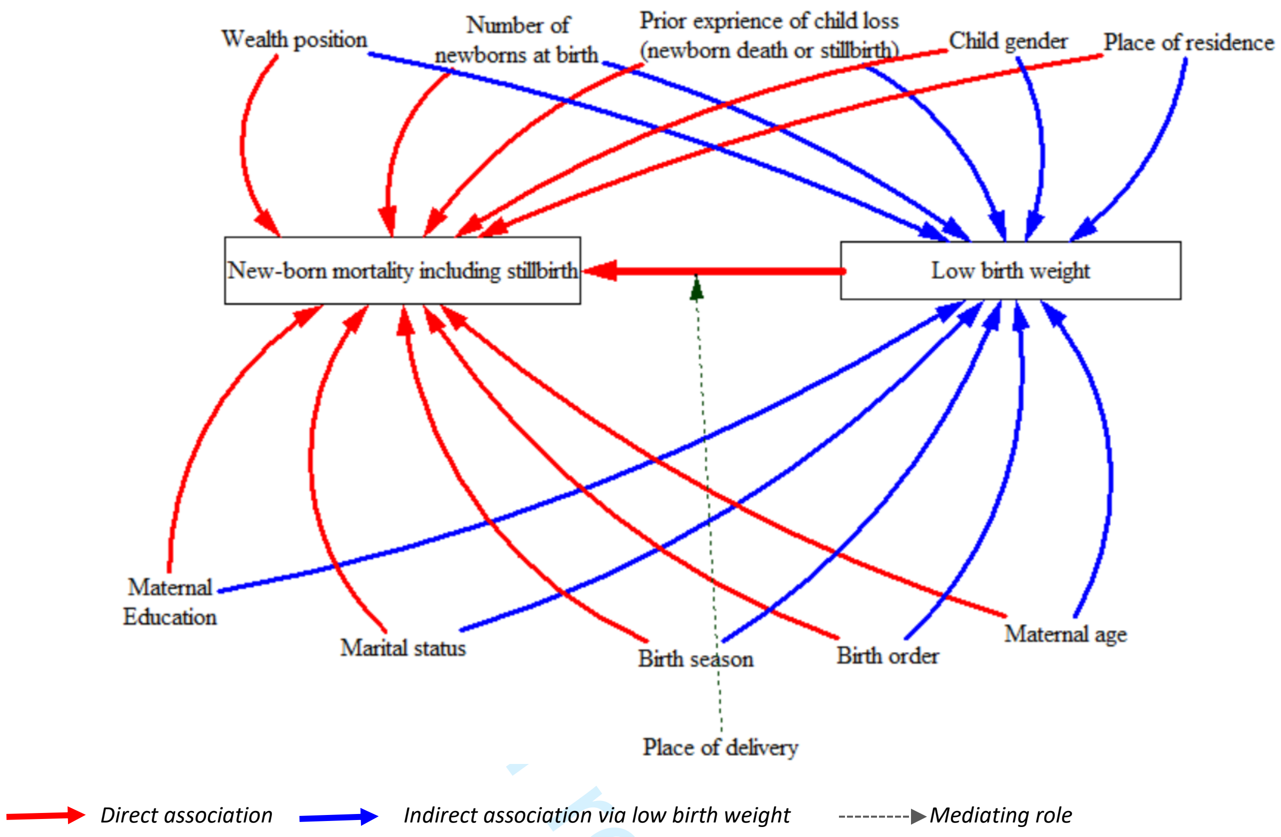

Sources: *Generated by the author based on available variables in the dataset and literature*

For peer review only - http://bmjopen.bmj.com/site/about/guidelines.xhtml

Supplement 2: Analysis of Mediation effect of LBW on newborn mortality

Let y_i represents the outcome of interest as a binary variable (i) that takes the probability of the presence of outcome's exposure as P and probability of absence of the outcome's exposure as 1-P. The bivariate regression model would be of the form as in Eq. 1

$$ln\left(\frac{P_j}{1-P_j}\right) = \alpha_i + \beta_i x_i \qquad (1)$$

Where *j* represents categories of perinatal mortality, late neonatal mortality, and LBW; x_i covariates of choice for each of the outcome and β_i their respective coefficients.

To assess the moderation effect of health facility delivery on the effect LBW on perinatal mortality (*pm*), an interaction term between the LBW and institutional deliver (*hf*) was introduced as indicated in Eq. 2.

$$ln\left(\frac{P_{pm}}{1-P_{pm}}\right) = \alpha_i + \beta_{lbw}x_{lbw} + \beta_{hf}x_{hf} + \beta_{lbw*hf}(x_{lbw} * x_{hf}) \qquad (2)$$

Subsequently, a multivariable regression model controlling for other factors added on eq. 2 was run (Eq. 3). In this model, I also assessed how the intersection between birth order and maternal age would affect perinatal mortality as shown in Eq. 3.

$$\begin{aligned} ln\left(\frac{P_{pm}}{1-P_{pm}}\right) = {} & \alpha_i + \beta_{lbw}x_{lbw} + \beta_{hf}x_{hf} + \beta_{lbw*hf}(x_{lbw} * x_{hf}) + \beta_{Age}x_{Age} + \beta_{educ}x_{educ} \\ & + \beta_{birth\ order}x_{birth\ order} + (\beta_{birth\ order*Age}(x_{birth\ order} * x_{Age}) \\ & + \beta_{multiple\ birth}x_{multiple\ birth} + \beta_{wealth}x_{wealth} \\ & + \beta_{nm\ expreinced\ preiviously}x_{nm\ expreinced\ preiviously} + \beta_{sex}x_{sex} \\ & + \beta_{birth\ quarter}x_{birth\ quarter} + \beta_{marital\ status}x_{marital\ \ status} \qquad (3) \end{aligned}$$

The independent variables that were included under the multivariate regression model for the late neonatal mortality (*nm*) are as indicated in Eq. 4

$$\begin{aligned} ln\left(\frac{P_{nm}}{1-P_{nm}}\right) = {} & \alpha_i + \beta_{lbw}x_{lbw} + \beta_{Age}x_{Age} + \beta_{educ}x_{educ} + \beta_{birth\ order}x_{birth\ order} \\ & + \beta_{multiple\ birth}x_{multiple\ birth} + \beta_{wealth}x_{wealth} \\ & + \beta_{nm\ expreinced\ preiviously}x_{nm\ expreinced\ preiviously} + \beta_{sex}x_{sex} \\ & + \beta_{birth\ quarter}x_{birth\ quarter} + \beta_{marital\ status}x_{marital\ \ status} \qquad (4) \end{aligned}$$

For peer review only

The mediating variable was selected if the predetermined endogenous – in this case, LBW was strongly associated with the main outcome variable in the multivariate model. The effect of selected covariates on LBW was determined using a multivariate regression model as indicated in Eq. 5

$$ln\left(\frac{P_{lbw}}{1-P_{lbw}}\right) = \alpha_i + \beta_{age}x_{age} + \beta_{educ}x_{educ} + \beta_{birth\ order}x_{birth\ order} + \beta_{multiple\ birth}x_{multiple\ birth} + \beta_{wealth}x_{wealth} + \beta_{nm\ expreinced\ preiviously}x_{nm\ expreinced\ preiviously} + \beta_{sex}x_{sex} + \beta_{birth\ quarter}x_{birth\ quarter} + \beta_{marital\ status}x_{marital\ status} \quad (5)$$

The indirect effect for instance for perinatal mortality with their corresponding standard errors for the factors that were significant in the LBW model were calculated as indicated in Eq. 6.

$$indirect\ \ effect_i = \beta_{lbw\ i} * \beta_{pm\ i} \quad (6)$$

The indirect effect's standard errors were calculated based on Goodman's approach of the product of coefficients [1] as indicated in Eq. 7.

$$\sigma_{\beta lbw\ i * \beta pm\ i} = \sqrt{\sigma^2_{lbw\ i} * \beta^2_{pm\ i} + \sigma^2_{pm\ i} * \beta^2_{lbw\ i} - \sigma^2_{lbw\ i} * \sigma^2_{pm\ i}} \quad (7)$$

The corresponding z or t -value were calculated as in Eq. 8;

$$z_i = \frac{indirect\ \ effect_i}{\sigma_{\beta lbw\ i * \beta pm\ i}} \quad (8)$$

Eq. 8 was used to calculate the P-values (P_i) for each indirect variable's coefficient as recommend by [2,3] in Eq. 9;

$$P_i = e^{-0.717138 * z_i - 0.415973 * {z_i}^2} \quad (9)$$

Reference

1 MacKinnon DP, Lockwood CM, Hoffman JM, *et al.* A comparison of methods to test mediation and other intervening variable effects. *Psychol Methods* 2002;**7**:83–104. doi:10.1037/1082-989X.7.1.83

2 Altman DG, Bland JM. Statistics notes: How to obtain the P value from a confidence interval. *BMJ* 2011;**343**:1–2. doi:10.1136/bmj.d2304

3 Altman DG, Bland JM. How to obtain the confidence interval from a P value. *BMJ* 2011;**343**:1–2. doi:10.1136/bmj.d2090

For peer review only

Supplement 3 Table 1: 2011-2015 Iganga-Mayuge HDSS event histories participants' descriptive statistics

	Complete case*		Imputed
	n=10,758		
	%ge	*missing*	*%ge*
**Institutional delivery**		**1.0**	
Public Health facility	68.7		68.7
Community	16.0		16.0
Private health facility	15.4		15.4
**Maternal education level**		**0.5**	
None	14.3		14.3
Primary	52.3		52.3
Post primary	33.4		33.4
**Maternal age group (years)**		**0.0**	
<20	13.7		-
20-29	53.6		-
30+	32.8		-
**Place of residence**		**0.0**	
Rural	64.0		-
Urban	36.0		-
**Marital status**		**9.1**	
Has a partner or married	86.2		86.2
Has no a partner	13.8		13.8
**Household wealth index**		**18.8**	
Index 1 and 2	46.0		43.7
Index 3	21.2		20.6
Index 4 and 5	32.8		35.7
**Previous experience of death**		**0.0**	
Experienced pregnancy loss	5.1		-
Never experienced pregnancy loss	94.9		-
**Childbirth weight**		**27.3**	
LBW (<2.5Kgs)	13.1		13.7
Normal Birth weight	86.9		86.3
**Childbirth order**		**1.2**	
First	45.0		45.0
2nd-4th	50.6		50.7
5th +	4.3		4.3
**Child sex**		**1.2**	
Female	50.2		50.2
Male	49.8		49.8
**Birth category**		**0.9**	
Multiple	2.4		2.4
Singleton	97.6		97.6
**Birth season in annual quarters**		**0.0**	

For peer review only

	Complete case*		Imputed
	n=10,758		
	%ge	*missing*	*%ge*
1	25.2		-
2	24.6		-
3	26.1		-
4	24.1		-
**Birth Year**		**0.0**	
2011	21.9		-
2012	21.6		-
2013	20.5		-
2014	17.9		-
2015	18.0		-
**New-born mortality**			
Deaths with 28 days (0-27 days)	2.2**		-
Deaths within 7 days (0-6 days)	1.9**		-
Stillbirths	1.19		-
Deaths within 7 days (0-6 days) +Stillbirths	3.1		-
Late neonatal mortality (7-27 days)	0.32***		

Note: ** the denominator excludes stillbirths (128), ***the denominator excludes those who died in the first seven days of live + stillbirths

For peer review only

STROBE Statement—Checklist of items that should be included in reports of ***cross-sectional studies***

	Item No	Recommendation	Reported on page #
**Title and abstract**	1	(*a*) Indicate the study's design with a commonly used term in the title or the abstract	1
		(*b*) Provide in the abstract an informative and balanced summary of what was done and what was found	2/3
**Introduction**			
Background/rationale	2	Explain the scientific background and rationale for the investigation being reported	3-5
Objectives	3	State specific objectives, including any prespecified hypotheses	5-6
**Methods**			
Study design	4	Present key elements of study design early in the paper	6/7
Setting	5	Describe the setting, locations, and relevant dates, including periods of recruitment, exposure, follow-up, and data collection	6/7
Participants	6	(*a*) Give the eligibility criteria, and the sources and methods of selection of participants	6/7
Variables	7	Clearly define all outcomes, exposures, predictors, potential confounders, and effect modifiers. Give diagnostic criteria, if applicable	7/8
Data sources/ measurement	8*	For each variable of interest, give sources of data and details of methods of assessment (measurement). Describe comparability of assessment methods if there is more than one group	7/8
Bias	9	Describe any efforts to address potential sources of bias	9/10
Study size	10	Explain how the study size was arrived at	NA
Quantitative variables	11	Explain how quantitative variables were handled in the analyses. If applicable, describe which groupings were chosen and why	7/8
Statistical methods	12	(*a*) Describe all statistical methods, including those used to control for confounding	10
		(*b*) Describe any methods used to examine subgroups and interactions	10
		(*c*) Explain how missing data were addressed	7
		(*d*) If applicable, describe analytical methods taking account of sampling strategy	na
		(*e*) Describe any sensitivity analyses	7/8
**Results**			
Participants	13*	(a) Report numbers of individuals at each stage of study—eg numbers potentially eligible, examined for eligibility, confirmed eligible, included in the study, completing follow-up, and analysed	11
		(b) Give reasons for non-participation at each stage	Na
		(c) Consider use of a flow diagram	Na

For peer review only - http://bmjopen.bmj.com/site/about/guidelines.xhtml

Descriptive data	14*	(a) Give characteristics of study participants (eg demographic, clinical, social) and information on exposures and potential confounders	11
		(b) Indicate number of participants with missing data for each variable of interest	11
Outcome data	15*	Report numbers of outcome events or summary measures	11
Main results	16	(*a*) Give unadjusted estimates and, if applicable, confounder-adjusted estimates and their precision (eg, 95% confidence interval). Make clear which confounders were adjusted for and why they were included	12-13
		(*b*) Report category boundaries when continuous variables were categorized	Na
		(*c*) If relevant, consider translating estimates of relative risk into absolute risk for a meaningful time period	na
Other analyses	17	Report other analyses done—eg analyses of subgroups and interactions, and sensitivity analyses	12
**Discussion**			
Key results	18	Summarise key results with reference to study objectives	13
Limitations	19	Discuss limitations of the study, taking into account sources of potential bias or imprecision. Discuss both direction and magnitude of any potential bias	19
Interpretation	20	Give a cautious overall interpretation of results considering objectives, limitations, multiplicity of analyses, results from similar studies, and other relevant evidence	13-18
Generalisability	21	Discuss the generalisability (external validity) of the study results	13-18
**Other information**			
Funding	22	Give the source of funding and the role of the funders for the present study and, if applicable, for the original study on which the present article is based	21

For peer review only
